# Somitogenesis Clock-Wave Initiation Requires Differential Decay and Multiple Binding Sites for Clock Protein

**DOI:** 10.1371/journal.pcbi.1000728

**Published:** 2010-04-01

**Authors:** Mark Campanelli, Tomáš Gedeon

**Affiliations:** 1 Department of Mathematics and Computer Science, Southwest Minnesota State University, Marshall, Minnesota, United States of America; 2 Department of Mathematical Sciences and Center for Computational Biology, Montana State University, Bozeman, Montana, United States of America; University of California, San Diego, United States of America

## Abstract

Somitogenesis is a process common to all vertebrate embryos in which repeated blocks of cells arise from the presomitic mesoderm (PSM) to lay a foundational pattern for trunk and tail development. Somites form in the wake of passing waves of periodic gene expression that originate in the tailbud and sweep posteriorly across the PSM. Previous work has suggested that the waves result from a spatiotemporally graded control protein that affects the oscillation rate of clock-gene expression. With a minimally constructed mathematical model, we study the contribution of two control mechanisms to the initial formation of this gene-expression wave. We test four biologically motivated model scenarios with either one or two clock protein transcription binding sites, and with or without differential decay rates for clock protein monomers and dimers. We examine the sensitivity of wave formation with respect to multiple model parameters and robustness to heterogeneity in cell population. We find that only a model with both multiple binding sites and differential decay rates is able to reproduce experimentally observed waveforms. Our results show that the experimentally observed characteristics of somitogenesis wave initiation constrain the underlying genetic control mechanisms.

## Author Summary

The vertebral column is a characteristic structure of all vertebrates. Individual vertebrae, together with ribs and attached muscles, develop from repeated embryonic structures called somites. The somite pattern forms in the embryo during somitogenesis. We know that this process uses periodic gene expression (a biomolecular “clock”) to generate the pattern, but we do not know precisely how this expression is controlled within the cell and coordinated across multiple cells. We propose a mathematical model that incorporates experimentally confirmed features of somitogenesis. We then test four different mechanisms that may control the clock and ask if the comparison between model simulations and experimental observation can select the best model and thus suggest how the clock is controlled. We find that the model scenario with both multiple DNA binding sites and differential protein decay rates is best able to reproduce experimental observations. Because these findings can be tested experimentally, our results should help guide future experiments.

## Introduction

Somitogenesis is the process by which vertebrate embryos develop somites, which are transient, repeated blocks of cells arising from the presomitic mesoderm (PSM) that differentiate further into vertebrae, ribs, musculature, and dorsal dermis. The tailbud is a proliferative zone at the posterior end of the embryo where immature cells are continually added to the posterior-most PSM. As the tailbud grows away posteriorly, the oldest cells in the anterior PSM segment in groups to form lateral pairs of somites along the midline. The process stops when the anterior formation of somites has progressed posteriorly across the entire PSM, reaching the arresting growth in the tailbud [1]–[4].

Somitogenesis is an impressively robust mechanism of pattern formation in developmental biology that has received much experimental and theoretical attention. In 1976, based on theoretical considerations, Cooke and Zeeman [5] postulated that somitogenesis proceeds by a “Clock and Wavefront” mechanism. In this model, the susceptibility of cells in the PSM to form somites oscillates between susceptible and insusceptible (the *clock*), while a determination *wavefront* sweeps posteriorly across the PSM. The passing wavefront triggers cells to form somites, but does so only when cells are susceptible, i.e., when their clocks are in the correct phase of oscillation. Since adjacent cells are in phase, cohorts of cells are recruited in succession to form somites. Initially, the clock was thought to be closely linked to the cell cycle [6]. In 1997, Palmeirim *et al.* [7] discovered a gene with oscillatory expression in the PSM of the chick embryo, providing an alternative candidate for the clock. Experimental work has since identified multiple oscillatory genes in each of several model organisms, including mouse [8] and zebrafish [2].

In all of these organisms, the oscillatory gene expression in individual cells is coordinated throughout the PSM in order to produce spatiotemporal waves of mRNA and protein expression, which we call the *clock-wave*. Synchronized, periodic expression is observed in the tailbud with a frequency that matches the anterior formation of somites [1],[2]. Broad waves of expression repeatedly initiate in the posterior-most PSM and narrow while traveling anteriorly [1]–[3],[7]. The waves slow considerably as they reach the region of forming somites. Successive waves arriving at the anterior-most PSM help sequentially establish stable bands of high-low gene expression in several additional genes, indicating nascent somite boundaries and polarity [1]–[3].

Separate experiments have identified biochemical candidates for the wavefront [9],[10]. These bio-molecules exhibit graded concentration profiles across the PSM that shift posteriorly in synchrony with tailbud growth. A changing gradient level triggers mesodermal cell differentiation and somite formation [1],[2],[11],[12]. We call this the *gradient-wavefront*.

The precise mechanism in which the clock-wave interacts with the gradient-wavefront, as well as their possible interactions with intercellular signaling mechanisms, remains unknown [1],[2],[12],[13]. Many mathematical models of the dynamics of somitogenesis have been proposed, with reviews and comparisons of several prominent models available in the literature [12]–[18].

Zebrafish is a standard model organism in the study of somitogenesis, and we now describe in more detail the mathematical modeling work in zebrafish that is most closely related to our present work. Lewis [19] has studied a two-cell model of the clock, where the oscillations arise from delayed, intracellular negative feedback of a protein dimer on its own mRNA production. In two different versions of the model, the clock consisted of one or two genes (*her1* and/or *her7*), and when both were included they interacted by protein heterodimerization. To synchronize gene expression between two neighboring cells, Lewis extended similar mammalian models [19]–[21] by introducing delayed, intercellular positive feedback via DeltaC/Notch protein signaling [22]. In 2006, Horikawa *et al.* [23] extended Lewis's model to a lateral line of synchronized cells in the PSM. Neither Lewis nor Horikawa's group addressed the posterior-to-anterior slowing of the oscillation rate that leads to formation of the clock-wave. However, Giudicelli *et al.* [24] experimentally quantified the slowing of oscillations in PSM cells, and measured model parameters such as mRNA production and transport delays and decay rates. Özbudak and Lewis [25] used this information to refine Lewis's two cell model and concluded that DeltaC/Notch signaling is used for coordinating oscillations between cells in the zebrafish PSM, but not for generating oscillations or forming somite boundaries.

Concurrently to the above work, a protein (Her13.2) was discovered in zebrafish that interacts with at least one of the clock-gene proteins [26],[27] and controls the rate of oscillatory expression in individual PSM cells, thereby inducing the formation of the clock-wave [26]. This protein is expressed in a graded fashion along the anteroposterior (AP) axis of the PSM. Based on this information, Cinquin [28] proposed a multicellular model for zebrafish somitogenesis that requires heterodimerization of two clock proteins (Her1 and Her7). In this model, formation of this heterodimer competed with the formation of other dimers, including heterodimers of each of the clock proteins with Her13.2. This competitive dimerization, combined with different levels of repression by the various dimers, produced waves of gene expression.

Her13.2 acts downstream of a morphogen gradient FGF, which is the presumed gradient-wavefront that controls somite formation in the anterior-most cells of the PSM [1],[2],[9],[10],[12],[26]. This suggests distinguishing between two distinct phases of somitogenesis; the first is the generation of a clock-wave in the PSM that narrows and slows as it propagates anteriorly, while the second is the commitment of cells in different phases of oscillation to different developmental pathways and somite formation.

In this paper we develop a biologically informed, yet minimally constructed, mathematical model that generates the initial narrowing and slowing of the clock-wave in the posterior PSM. Our model incorporates the delayed, intracellular negative feedback model of Lewis [19] for the clock and was motivated by the results of Cinquin [28], in which competitive dimerization of clock proteins with a graded control protein contributes to the slowdown of clock oscillations. Our multicellular model retains much of the simplicity of Lewis's deterministic, single clock-gene model with intercellular coupling, incorporating a minimum of additional biological components to generate the experimentally observed posterior clock-wave in zebrafish. Our main goal is to determine if this experimentally observed aggregate behavior of the clock-wave is sufficient to constrain the genetic control mechanisms responsible for the oscillatory gene expression of the clock.

We consider two different genetic control mechanisms, giving four different model scenarios with either one or two binding sites for the self-repressing clock protein homodimer, and where either only monomers of clock protein decay or where both monomers and dimers decay linearly with the same rate constant. The differential decay of monomers and dimers is an example of *cooperative stability*, which was found to have a significant impact on behavior of a bistable switch and the repressilator in [29]. We parametrize the model to the extent possible with experimentally determined parameters from zebrafish [19],[24],[25], but in each model scenario there are a number of parameters with unknown values. We uniformly sampled 40,000 combinations of the unknown parameter values from a biologically realistic range and tested if each model scenario was able to reproduce the experimental data at each sampled parameter combination. Two main experimental observations that the model must match are tailbud clock period of 30 minutes to within 10%, and sufficient decrease in the oscillation rate along the axial PSM in order to generate the observed clock-wave. We find that only the model scenario that combines two binding sites for the clock protein repressor and different decay rates of the clock protein monomers and dimers is able to accurately reproduce both experimental observations.

Sensitivity of clock-wave formation to each estimated parameter is investigated by analyzing the successful combinations of parameters. We find sensitivity with respect to clock mRNA transcriptional delay (in agreement with [19]), clock protein homodimer binding affinity to DNA, its binding cooperativity and protein dimerization constants.

To further confirm model validity, we test the optimal model's robustness to heterogeneity in the cell population. For the best choice of estimated parameters, random perturbation in each cell of 22 parameters around their nominal values produces a heterogeneous population of cells. We selected size of our perturbations so that, on average, the majority (99.7%) of parameters lie within 1% or 2.5% of their nominal values. We test two spatial arrangements of heterogeneous cells: a line of fifty cells along the anterior-posterior axis and 250 cells arranged in five parallel rows along the AP axis. We find that oscillation and clock-wave formation in the PSM is robust to cell heterogeneity at these levels, although we observed a disorganization reminiscent of the salt-and-pepper patterns seen in many DeltaC/Notch knockout/knockdown experiments (e.g., Figure 3l in [22]) at the 2.5% level of heterogeneity.

Based upon our mathematical model, we conclude that the experimentally observed behavior of the clock-wave significantly constrains the genetic control mechanisms responsible for the clock behavior. The necessity of multiple binding sites for the self-repressive clock protein homodimer verifies an existing hypothesis for the genetic control mechanism of the clock [30]–[32]. Furthermore, very recently and after our paper had been submitted, Brend and Holley [30] experimentally identified two active dimer binding sites for the *her1* clock-gene in zebrafish. This result is highly encouraging for our modeling work, even though we concentrate on the clock protein *her7*. The necessity of differential decay rates for clock protein monomer and dimer represents further confirmation that the molecular dynamics can be significantly affected not only by the nonlinearities in the production of molecular species, but also the nonlinearities in the decay process [29]. The hypothesized nonlinear decay mechanism may be an important alternative and/or complement to rate-limited protein decay mechanisms studied in [33]–[35] and warrants experimental investigation.

## Results

Our mathematical model of the clock-wave in the posterior PSM had three components: 1) a clock-gene with oscillatory expression in each cell (clock), 2) a spatiotemporally graded control protein that controlled the clock's oscillation rate (control protein), and 3) a signaling gene whose protein signal coupled oscillations between cells (coupling signal). These components are present in the standard model organisms, including zebrafish, chick, and mouse [1]. The mathematical model is given by a system of delay differential equations and is described in detail in the Models section.

We applied our model to zebrafish, which has several basic helix-loop-helix (bHLH) clock-genes with oscillatory expression in the PSM, including *her1*, *her7*, *her11*, *her12*, and *her15* [2]. Two prominent bHLH clock genes, *her1* and *her7*, have received considerable experimental attention, and these genes' expression is synchronized throughout the PSM [2],[36], yet their respective roles in forming the clock-wave are not completely clear [2],[32],[37]. *her7* was chosen as the single clock gene in our model because the posterior clock-wave still forms during Her1 protein knockdown, even though waves fail to propagate anteriorly [32],[37].

In zebrafish, expression of the bHLH protein Her13.2 is highest in the tailbud and decreases anteriorly [26],[27]. Her13.2 protein was chosen to represent the externally prescribed control protein in the model because it likely heterodimerizes with other bHLH proteins, in particular, Her1 and Her7 [26]. Because Her13.2 proteins have a truncated amino acid sequence normally used for DNA binding [26], we assumed that neither Her13.2 homodimers nor heterodimers with Her7 can repress *her7* mRNA transcription. Thus, Her13.2 influenced Her7 self-repression only through competitive dimerization, as seen in related bHLH networks [38]. Dimerization reactions were assumed to be very fast relative to other production and decay processes.

*deltaC* was chosen as the primary coupling-signal gene because its expression in the PSM is oscillatory and synchronized with *her7* expression [39]. DeltaC ligands presented through a cell's membrane activate Notch proteins in adjacent cells' membranes, triggering a cascade that up-regulates clock-gene expression, including *her7* [39],[40]. Following the model in [19], *deltaC* expression was presumed to be inhibited by Her7 homodimer, which allows oscillatory expression of the clock-gene to drive synchronized oscillatory expression of the coupling-signal gene.

Experimental data for zebrafish provided the following constraints on the clock-wave behavior:

- The oscillation period in the tailbud is 30 minutes at 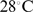, which is the same time it takes for each somite to form in the anterior PSM [2].
- The oscillation rate in the more anterior PSM slows sufficiently to generate a clock-wave with two to three traveling bands of gene expression, which emanate from the tailbud and narrow as they reach the anterior-most PSM [2],[24].

These two constraints were used to examine the effect of two control mechanisms, which have been implicated in other systems, on the proper formation of the clock-wave:

1. The number and cooperativity of binding sites for the self-repressive clock protein [41],[42].
2. Differential decay rates of the repressor dimers and monomers [29].

Experimental evidence exists in zebrafish, chick, and mouse both for protein dimerization and for multiple *cis* regulatory sites for clock-genes [28], [30]–[32],[38].

### Parameter Estimation and Model Selection

We considered four model scenarios that differed in the clock-gene control and protein decay mechanisms. In scenario I, we assumed a single binding site for the self-repressing clock-protein homodimer and that only clock-protein monomers decay. In scenario II, we still considered a single binding site, but instead assumed that clock-protein monomers, homodimers, and heterodimers with the control protein all decay with the same rate constant. In scenario III, we assumed two binding sites for the self-repressing homodimer and monomer-only decay. Lastly, in scenario IV, we assumed two binding sites and decay of all forms of the clock protein. Table 1 gives the choice of model parameters corresponding to each scenario.

**Table 1:** Model Scenario Specifying Parameters.

Scenario	Parameter	Description	Value	Units	Source
I	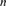	DNA binding sites	1	—	[31]
	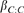 , 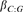	dimer decay constants	0	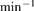	[29]
II	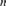	DNA binding sites	1	—	[31]
	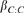 , 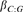	dimer decay constants	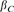	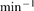	[29]
III	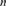	DNA binding sites	2	—	[31]
	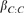 , 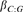	dimer decay constants	0	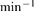	[29]
IV	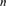	DNA binding sites	2	—	[31]
	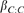 , 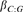	dimer decay constants	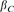	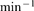	[29]

The parameters specifying each model scenario. The last column indicates a source for the value of the parameter. 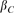 is the decay constant for clock-protein monomer, which was estimated from a biologically realistic range. More details on parameter selection appear in Text S1.

Through numerical simulation of the mathematical model we assessed the ability of the above four model scenarios to:

- Produce synchronized periodic expression of the clock and coupling-signal genes in the tailbud within 10% of the experimentally observed value (30 minutes).
- Produce sufficient decrease in the oscillation rate between the tailbud (high level of total control protein Her13.2) and the more anterior PSM (low level of total control protein Her13.2).
- Produce a realistic posterior clock-wave in a simulated anterior-to-posterior line of 50 cells with two properly spaced, posterior-most expression bands of the clock and coupling-signal mRNA.
- Exhibit robustness of clock-wave formation with respect to heterogeneity in the parameters across the cell population.

Wherever possible, we used experimentally determined parameter values in the model. However, for ten parameters, including minimal (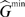) and maximal (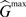) total control protein levels, dimerization dissociation constants, clock mRNA production delay, clock monomer decay rate, and clock homodimer binding affinities, only a feasible range of values was known. Tables 2 and 3 summarize the values and ranges of the model parameters and Text S1 includes details on the parameter selection process. We searched this space of parameters for those sets that reproduce experimental clock-wave. A parameter set was considered to produce a valid fit to experimental data if the corresponding model simulation satisfied criteria (a)–(d).

**Table 2:** Estimated Model Parameters.

Parameter	Description	Range	Units	Source
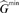	minimum total control protein (in PSM)	0–2500	copy number	—
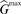	maximum total control protein (in tailbud)	0–2500	copy number	—
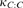 , 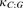 , 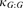	dimer dissociation constants	10–1000	copy number	[29]
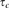	clock mRNA production delay	2.3–8.1	min	[24]
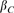	clock-protein monomer decay constant	0.2–0.5	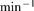	[47]
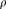	RNAP-II binding affinity constant	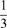 –3	—	—
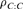	clock homodimer binding affinity	0.01–1	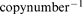	[19], [47]
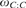	clock homodimer binding cooperativity	1–100	—	[47]

The parameters whose values were estimated from a range. The last column indicates a source for the range of the parameter. More details on parameter selection appear in Text S1.

**Table 3:** Fixed Model Parameters.

Parameter	Description	Values	Units	Source
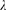	somite diameter	5	cells/somite	[2]
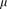	somite formation rate	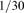 †	somites/min	[2]
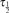	half-life of control protein	60	min	—
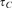	clock protein translation delay	1.7	min	[19]
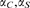	protein production constants	4.5	protein copies/mRNA/min	[19]
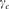 , 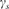	mRNA production constants	33	mRNA copies/min	[19]
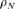	activator binding affinity	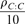	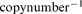	[25]
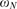	activator cooperativity	25	—	[25]
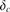	clock mRNA decay constant	0.206	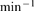	[25]
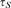	signal protein translation delay	20	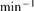	[19]
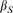	signal protein decay constant	0.23	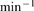	[19]
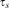	signal mRNA production delay	12.4	min	[25]
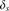	signal mRNA decay constant	0.273	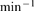	[25]

**†:** This is a default value, see Text S1 for details.

The values of parameters that were fixed in all simulations. The last column indicates a source for the value of the parameter. More details on parameter selection appear in Text S1.

The following important observation allowed parameter estimation to proceed in two stages: formation of a realistic clock-wave in a large simulation of fifty cells along the AP axis of the embryo depends upon the key value of 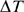, defined as the maximum change in clock oscillation period observed over a range 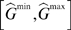 of total control protein 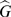. The parameters 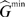, 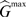, and 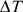 were estimated by simulation of a smaller simulation of two identical, coupled cells by increasing 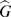 in steps of ten from 0 to 2500 copies per nucleus and recording the period of the oscillation at each step. Parameter combinations giving 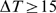 minutes were observed to generate a biologically realistic posterior clock-wave in the large simulation of fifty cells (also see [24]).

Therefore, in the first stage, we took a random sample of size 40,000 from a joint distribution of the remaining eight estimated parameters (Table 2, see Model Simulation and Selection in the Models section for more details.) For each parameter set we simulated two identical, coupled cells in each of the four model scenarios. By stepping through the values of 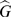 from zero to 2500, we determined if there were values 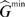 and 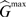 for which a given model scenario satisfied criteria (a) and (b) with 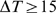 minutes. In the second stage, an AP line of fifty coupled cells with a spatiotemporally graded control protein was simulated to verify that the selected parameter set from stage one indeed produced a realistic clock-wave in the absence (c) and presence (d) of cell heterogeneity. Details of the simulation procedure are described in the Models section.

**Figure 1:**
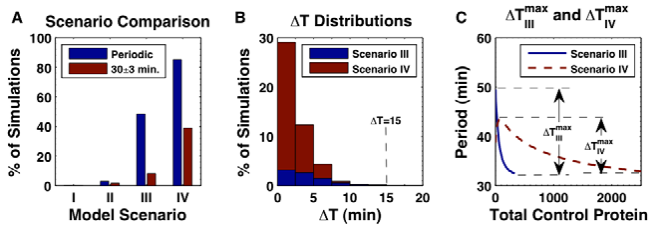
Model selection. (A) Blue bars: the percentage of periodic solutions in 40,000 simulations for some level of total control protein 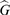 in the range 0–2500 copies/nucleus. Red bars: the percentage of periodic solutions in 40,000 simulations also exhibiting the tailbud period of 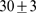 min. (B) Stacked distributions of 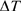 for model scenarios III and IV for those solutions exhibiting the tailbud period. In scenario III, solutions exist with 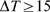 minutes, a key requirement for proper clock-wave formation in zebrafish. Raw data for this graph can be found in Text S1. (C) Period as a function of total control protein level for the optimal parameter selections for model scenarios III and IV, which produce 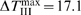 min and 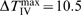 min, respectively. The curve for model scenario III stops at 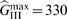 copies/nucleus, after which the amplitude of the periodic solution drops below 5 copies/nucleus, peak-to-peak. Periodic solutions cease altogether at 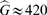. Note that 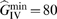 because the period is a non-monotone function of the total control protein level, with a maximum at 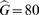.

For model scenarios I–IV, the first two lines of Table 4 list: 1) the number of parameter sets out of 40,000 total selections that produced periodic solutions in two coupled cells for some level of 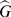 in 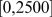, and 2) the number of parameter sets for which the periodic solution also exhibited a period of 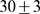 minutes for some level of 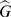. Figure 1A presents the same data using percentages rather than raw counts. The last two lines of Table 4 list the number of parameter sets that support periodic solutions with period 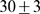 minutes for some level of 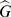 while also producing the indicated differences in period 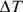 over some interval 
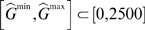
.

**Table 4:** Model Validation Results; Stage 1.

Solution Criteria\Scenario	I	II	III	IV
Periodic	23	1201	19361	34108
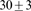 min.	0	627	3247	15509
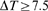 min.	0	0	346	131
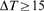 min.	0	0	8	0

Number of parameter sets (out of 40,000) in which model simulation produced a periodic orbit (first row) and a periodic orbit with period 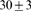 minutes (second row) at some fixed level of total control protein taken from a range. The number of parameter sets that further produced a period change of 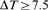 minutes (third row) and of 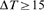 minutes (fourth row) are also indicated. Columns correspond to different model scenarios.

The vast majority of solutions that exhibited sustained oscillations with a period of 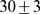 minutes, and thus satisfied criterion (a) above, were for scenarios III and IV with two clock protein binding sites. For scenarios I and II, with a single clock protein binding site, the largest 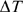 was 3.4 minutes, and so neither scenario satisfied criterion (b) above. For scenarios III and IV, Figure 1B shows the distribution of 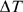 for those simulations that produced a period of 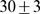 minutes. The important observation is that even though scenario IV produced the required period of oscillation 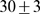 minutes for almost 40% of parameter sets (as opposed to 8% in scenario III, see Figure 1A), the maximum period change 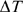 for scenario IV was 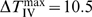 minutes. This was smaller than the 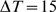 minutes necessary for realistic clock-wave formation. In scenario IV, less than 1% (131 out of 15509) of the parameter sets that produced a period of 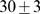 also produced 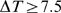. In contrast, for scenario III, 10.6% (346 out of 3247) of the parameter sets that produced a period of 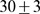 minutes also produced 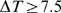. Eight out of 3247 parameter sets in scenario III produced 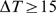, and the maximum period change was 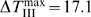 minutes, see Table 4. We remark that since 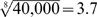 the choice of 40,000 parameter sets in 8 dimensional space, if spaced in a regular grid, only gives 3 to 4 different values for each parameter, so that when the parameter sets are chosen randomly each set represents a significant volume of the parameter space. Viewed in this light our success rate of parameter sets that produce 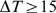 is not disappointing. Figure 1C shows the oscillation period as a function of the total control protein in scenarios III and IV for the parameter sets that produced 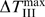 and 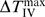, respectively. The parameter set selections that produced these optimal 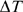 values are given in Table 5. See Text S1 for a complete tabulation of results.

**Table 5:** Model Validation Results: Optimal Parameter Sets.

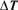 \Parameter	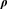	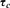	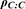	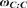	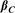	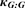	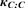	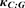	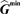	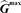
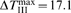	1.09	4.23	0.845	47.6	0.393	94.4	133	19.3	0	330
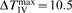	0.499	5.93	0.578	14.8	0.210	982	738	12.4	80	2500

Optimal parameter sets for model scenarios III and IV, which produced the largest change in oscillation period, 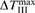 and 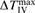, respectively.

Since no parameters for scenario I produced oscillations with the required period of 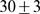 minutes, we concluded that a single binding site with differential protein decay is not capable of producing the experimentally observed oscillations in the zebrafish tailbud. Scenarios II and IV, with equal monomer and dimer clock protein decay rates, did not produce sufficient 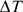 over the given range of total control protein 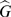. Only scenario III, combining two binding sites and monomer-only clock protein decay, admitted a significant number of parameter sets that produced 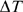 large enough to generate a biologically realistic clock-wave.

**Figure 2:**
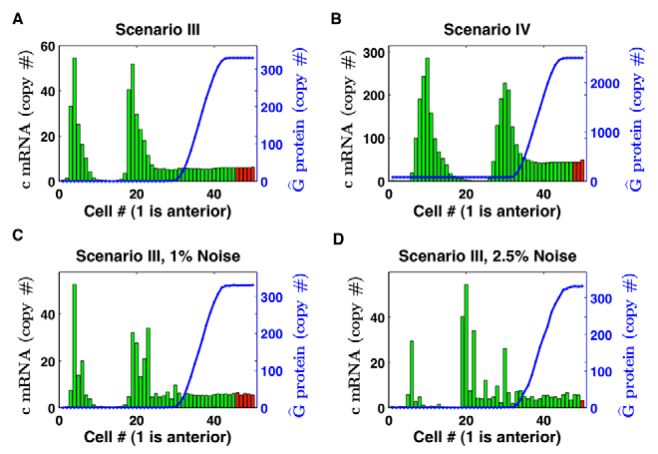
Simulated clock-waves. Clock-waves in 50 cells for parameter set selections giving (A) 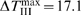 min, and (B) 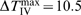 min. Model scenario III produces more tightly spaced peaks in the posterior PSM than scenario IV, compare Videos S1 and S2. (C) Robustness of clock-wave formation to normally distributed perturbation with 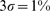 added independently to all positive parameters in all cells, with a non-negativity constraint; (D) same as in (C), but with 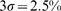 of the nominal parameter values used in (A), compare Videos S3 and S4. Red cells are in the tailbud and green cells have exited the tailbud into the PSM. The scenario III solutions (A, C, and D) and the scenario IV solution (B) occur 289 and 305 minutes, respectively, after the first cell has exited the tailbud.

The second validation stage verified proper clock-wave generation across a growing AP line of fifty coupled cells in the axial PSM. In these simulations, a sigmoidal spatiotemporal gradient of the total control protein was prescribed across the cells in the PSM, decreasing from 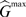 in the tailbud to 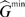 anteriorly. Figure 2A–B compares the simulated mRNA clock-waves for model scenarios III and IV using the parameter sets that produced 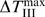 and 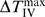, respectively. Note that in Figure 2A, the spacing of the posterior-most bands of clock-gene mRNA expression is 14–15 cells (which narrows to 13 cells toward the anterior PSM), comparable to the mean value of approximately 10.5 cells measured experimentally for *her1* in zebrafish (see Figure 3 in [43]), whose expression is synchronized with *her7* [2]. In contrast, the spacing in Figure 2B is about 20 cells, which is considerably larger than the experimentally observed spacing. Videos S1 and S2 show movies of the simulations for scenarios III and IV, respectively.

### A Mechanism for Oscillation Rate Tuning

**Figure 3:**
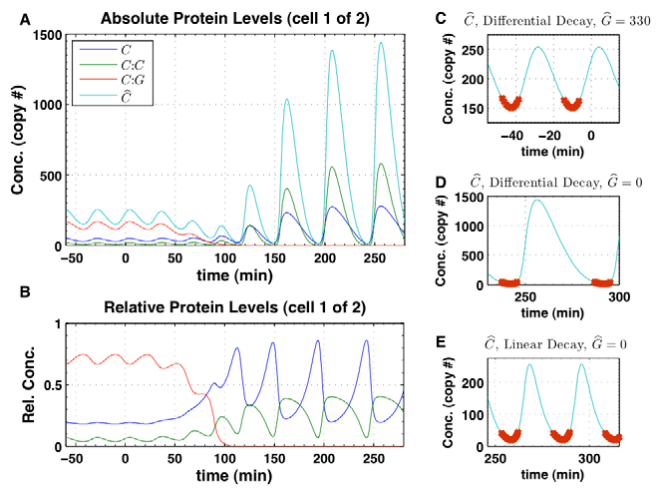
Oscillation rate tuning. The mechanism of oscillation rate tuning by the decreasing level of total control protein between the tailbud and intermediate PSM. Clock protein levels are shown for the first of two coupled cells for the parameter set selection giving 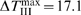 min with differential decay (A–D), and with differential decay changed to linear decay (E). (A) Absolute levels of clock monomer, homodimer, heterodimer with control protein, and total clock protein, and (B) levels of monomer and dimers relative to the total clock protein level. Total control protein decays from 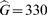 to 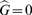 starting at time 0. (C–E) Close-ups of the oscillation in (C) the tailbud and (D) the intermediate PSM for differential decay, and (E) the oscillation in the intermediate PSM for linear decay. The red part of the curve denotes the time when significant production of clock mRNA takes place (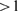 copy/min) as determined from the mRNA production curves in Figure 6 in Text S1 and delayed by a production delay 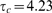 minutes.

The differential decay of monomers and dimers (cooperative stability) and two binding sites for the repressor dimers combined to produce a significant change in oscillation rate between the tailbud and the intermediate PSM. The cooperative stability effect was similar to that discussed in [29]: since the proportion of dimers to monomers increases with the total concentration of protein, the marginal decay rate (i.e., decay per unit of total protein) decreases with total concentration. This effect can be seen in Figure 5 of Text S1 where we compare the linear decay rate and the differential decay rate as a function of total protein concentration, and in Figure 3B where we graph the relative quantities of monomer and dimers during oscillations.

The two binding sites primarily affected the production of the clock mRNA, because they increased the effective Hill coefficient of the nonlinearity. Figure 6 in Text S1 compares the nonlinear production curve of clock mRNA as a function of total clock protein level. The production curves for scenarios III and IV (two binding sites) were shifted toward low levels of total clock protein as compared to production curves for scenarios I and II (single binding site). Note that significant production of clock mRNA occured only in a limited part of the oscillation cycle of two coupled cells (red part of the curve in Figure 3D) for the lowest levels of total clock protein. Since it took a longer time for the total clock protein to decay to this low value, the shifted production curve also enhanced the length of the period. These two effects combined to cause a slow decay of the total clock protein from its peak, compare Figure 3D to Figure 3E where we replaced differential decay by linear decay of total clock protein.

So far we have discussed how cooperative stability increased the period of the oscillation in the PSM where total control protein 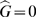. However, the key to clock-wave formation across many cells is the change in oscillation rate between the tailbud, where 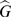 is high, and the intermediate PSM, where 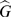 is low (cf., the value of 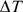 computed in the two-cell simulations in stage one of model selection). For two coupled cells, Figure 3A shows the absolute levels of clock monomer, homodimer, heterodimer with control protein, and total clock protein as the level of total control protein was decreased dynamically from 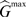 to 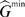. Figure 3B shows these monomer and dimer levels relative to the total clock protein level. In the tailbud, a significant proportion (about 75%) of clock protein was bound in the heterodimer and as a result of this buffering, the oscillations were small in amplitude and more symmetric, see Figure 3C. After the level of total control protein 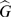 dropped, the oscillator was released from the buffering, the mRNA production curve shifted toward smaller values of total clock protein (Figure 6 in Text S1), and both the amplitude and the period of the oscillation rapidly increased. The transition from high 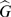 to low 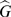 caused a transition from gentle, faster oscillations to slower, burst-like oscillations.

The results in Figure 3 were from a simulation of two coupled cells. We examined the effect of the coupling signal on the change in oscillation rate by repeating this simulation for a single cell and found negligible differences in oscillation rates. Therefore, we graphed the production curves in Figure 6 in Text S1 for the mean value of coupling signal (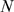) in the respective regions (tailbud or PSM).

### Sensitivity of the Clock-Wave

We examined the sensitivity of eight estimated parameters in each model scenario. We first selected nested collections 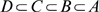 of the 40,000 random parameter sets by imposing increasingly stringent requirements on the corresponding solution: 1) (collection A) parameter sets for which the solution was periodic for some level of total control protein in the range 0–2500 copies per nucleus, 2) (collection B) parameter sets for which the solution satisfied 1) and had a period of 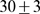 minutes for some level of total control protein in the range 0–2500 copies per nucleus, 3) (collection C) parameter sets for which the solution satisfied 1) and 2) and had a period change 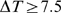 minutes over a range of total control protein, and 4) (collection D) parameter sets for which the solution satisfied 1) and 2) and had a period change 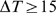 minutes over a range of total control protein. A period change of at least 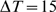 minutes is sufficient for generating a biologically realistic posterior clock-wave for zebrafish. Inclusion of collection 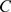 (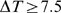) allowed direct comparison between scenarios III and IV.

**Figure 4:**
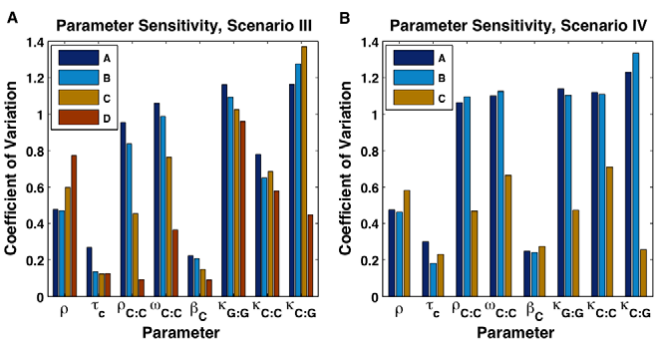
Parameter sensitivities for model scenarios III and IV. Bars indicate the coefficient of variation (C.V.) of 8 individual parameters from collections A–D of parameter sets out of 40,000 total parameter sets whose simulated solutions for (A) scenario III and (B) scenario IV satisfy the corresponding selection criteria described in the text. Note that the number of parameter sets used to compute the C.V.'s decreases in each collection from A to D.

Figure 4 shows the coefficient of variation (C.V.) of each of the eight parameters in collections 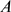
–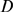. Text S1 contains histograms showing projections of each collection onto the individual parameters for scenarios III and IV. The C.V.'s for each parameter for each collection were computed from the corresponding distributions.

Small values of C.V. show that the parameter value is tightly determined by the particular requirement 1)–4) and hence the wave formation is sensitive to this parameter. A decreasing C.V. value from left to right signifies increasing sensitivity as a function of more stringent requirements. As expected from the model selection discussion, the largest selective pressure on the parameter sets was imposed by the requirement for 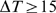. Inspection of the C.V. data (Figure 4) and the corresponding histograms (Text S1) suggested the following. In both scenarios III and IV, attaining the proper period of oscillation (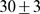) was most sensitive to the clock mRNA production delay 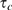. Furthermore, attaining sufficiently large 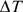 for clock-wave formation was most sensitive to the clock homodimer binding affinity 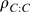 and cooperativity 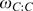, the clock monomer decay rate 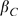, and the dimer dissociation constants 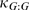, 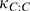, and 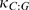. While both scenarios showed sensitivity of the dimer dissociation constants to increasing 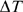, scenario IV showed additional sensitivity to the heterodimer dissociation constant 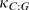. Finally, we note that the parameter sets that belong to collection 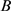, but not to collection 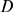 support oscillation with period of about 30 minutes, but do not produce sufficient 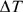 which would lead to successful clock-wave formation. This suggest that there may be mutants where a change in certain parameter values will produce uniform oscillation throughout PSM and thus the clock wave initiation will fail. If the estimated parameters in this study can be experimentally measured, then our dataset can be mined for related parameter sets for which little or no change in oscillation rate occurs with changing levels of total control protein.

### Robustness to Cell Heterogeneity

We examined if the optimal scenario III solution was robust to cell heterogeneity.

#### Robustness of oscillations and clock-wave formation

The robustness of clock-wave formation to heterogeneity in the cell population was examined by randomly perturbing parameter values in each cell. Figure 2C–D represents model scenario III from Figure 2A, but with two different levels of normally distributed noise with standard deviation 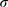 and with a non-negativity constraint added to all positive parameters in each cell.

We selected size of our perturbations so that, on average, the majority (99.7%) of parameters lie within 1% or 2.5% of their nominal values. Since the nominal values are the means of the parameter distributions, these choices correspond to 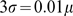 and 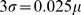 which can be expressed in terms of C.V. as 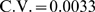 and 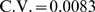 for each of the 22 parameters. Comparison to histograms in Figure 4 shows that 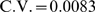 is about one tenth of the size of maximal perturbation that allows formation of the proper clock wave in homogenous population of cells. Our final comment concerns the size of the parameter space. In 22 dimensional space, the diagonal in a hypercube with each side of size 1 has diagonal of length 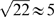. Thus small perturbation in each dimension leads to significant total distance between 22 dimensional parameter sets and large heterogeneity in cell populations. In this heterogenous population clock-wave still formed, see Figure 2C/Video S3 and Figure 2D/Video S4, respectively.

The oscillatory expression of each individual cell persisted for perturbations larger than 2.5%, but the cells in the tailbud drifted increasingly out of synchrony and the clock-wave showed increased disorganization. At 2.5% noise, and after 500 minutes of solution settling time from zero initial history, the period of at least one of the fifty cells in the tailbud differed relative to the population's mean period by more than 1%. This suggests a critical role of synchronization of cells in the tailbud.

**Figure 5:**
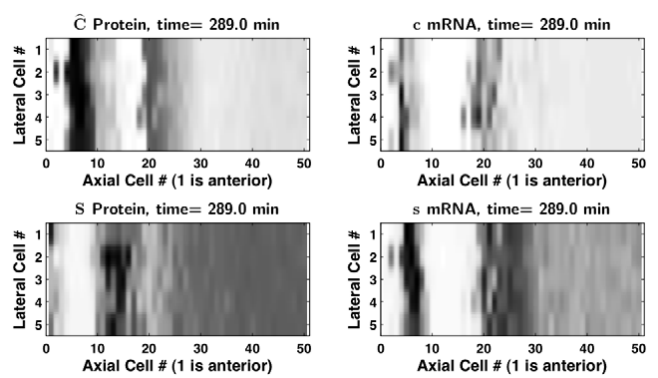
Simulation of a 2D array of cells with low cell heterogeneity. Clock-wave in a rectangular array of 50 axial by 5 lateral cells, for the parameter set selection giving 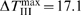 min, with an independent, normally distributed perturbation of all positive parameters in all cells, with 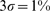 of the nominal parameter values and a non-negativity constraint. Darker grey indicates a higher expression level. Interior, edge, and corner cells are coupled to their eight, five, and three adjacent nearest neighbors, respectively. Model scenario III, with sufficiently large 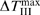, produces more tightly spaced peaks in the posterior PSM than scenario IV. Clock-wave formation is robust to the presence of parameter noise. Also see Video S5.

To test if the effect of Notch synchronization signaling is significantly different in a two-dimensional array of cells when each cell is surrounded by more than two neighboring cells, we simulated five parallel lines of 50 heterogenous cells on a rectangular grid and with nearest-neighbor intercellular coupling (cells that touch at corners were considered to be neighbors). Figure 5 and Video S5 show that at a 1% noise level the cells in the tailbud stayed reasonably synchronized and the clock-wave formed robustly.

**Figure 6:**
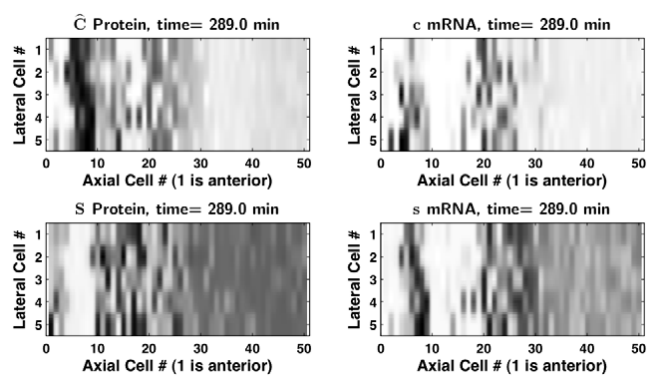
Simulation of a 2D array of cells with high cell heterogeneity. Clock-wave in a rectangular array of 50 axial by 5 lateral cells, for the parameter set selection giving 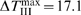 min, with an independent, normally distributed perturbation of all positive parameters in all cells, with 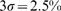 of the nominal parameter values and a non-negativity constraint. Darker grey indicates a higher expression level. Interior, edge, and corner cells are coupled to their eight, five, and three adjacent nearest neighbors, respectively. Also see Video S6.

At 2.5% noise, and after 500 minutes of solution setting time from zero initial conditions, the period of at least one of the 250 cells in the tailbud differed relative to the population's mean period by more than 1%. Although there was disorganization reminiscent of the salt-and-pepper patterns seen in many DeltaC/Notch knockout/knockdown experiments (e.g., Figure 3l in [22]), the formation of a clock-wave was still noticeable (Figure 6 and Video S6). We concluded that the greater Notch signaling from additional cell neighbors did not lead to significantly stronger synchronization.

### Simulation of Zebrafish Experiments

We examined if the optimal scenario III solution was able to reproduce several experiments reported in zebrafish.

#### Her1 only knockdown

The present model, with a single clock-gene, reproduced the posterior clock-wave formation observed in Her1 morpholino knockdown zebrafish, see Figure 2A and Video S1. In these experiments, the remaining clock-genes (including *her7*) apparently maintain formation of the posterior-most expression band of the clock-wave (Figure 4K in [32]). In contrast to the computational result shown in Figure 5A and Video S4 in [28], our model does form a posterior band of expression instead of a much broader residual oscillation. Because our model aimed to describe clock-wave initiation in the posterior-most PSM, it was not expected to reproduce the failure of the clock-wave to propagate into the anterior PSM.

#### Her1+Her7 combined knockdown

**Figure 7:**
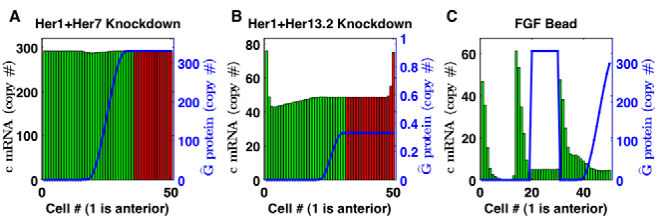
Simulated zebrafish experiments. Simulated zebrafish experiments for 50 cells for the parameter set selection giving 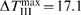 min. Red cells are in the tailbud and green cells have exited the tailbud into the PSM. (A) Replication of Her1 and Her7 combined morpholino knockdown. There is a 99.9% reduction in the Her7 clock protein production rate. Expression does not oscillate and is decreased slightly in the transition region of the total control protein (Her13.2). Also see Video S7. (B) Replication of Her1 and Her13.2 combined morpholino knockdown. There is a 99.9% reduction in the Her13.2 total control protein levels. Expression oscillates in a nearly spatially uniform manner. Also see Video S8. (C) Replication of an FGF bead grafting experiment. The presence of the bead causes ten cells to maintain an ectopically high level of Her13.2 total control protein, which lowers the amplitude of oscillation in this region after cells have entered the PSM. Also see Video S9. Solutions A, B, and C occur 205, 307, and 350 minutes, respectively, after the first cell has exited the tailbud.

Figure 7A shows a simulated Her1 and Her7 combined knockdown experiment reported by Oates and Ho [37]. Reducing clock protein production to 0.1% of its original value (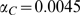) abolished the clock-wave, generating a steady distribution of *her7* mRNA that qualitatively agreed with the experiment in [37] (see also Video S7 and compare to Figure 9O in [37]). This should be compared to the computational result shown in Figure 5C and Video S6 in [28], in which steady expression of clock-genes in the tailbud transitioned into an oscillatory pattern of cells in the PSM that followed the posterior movement of the *her13.2* gradient.

#### Her1+Her13.2 combined knockdown

Because our model did not incorporate *her1*, we simulated the combined Her1/Her13.2 knockdown experiment reported by Sieger *et al.* (see Figure 2J and Figure 4 in [27]) by setting the total control protein level to 0.1% of its normal level (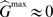). This experiment produced a uniformly oscillating expression of *her7* mRNA across the posterior PSM, see Figure 7B and Video S8. Note that the boundary effects are caused by cells at the boundary being exposed to half the Notch signaling, since they are only coupled to a single neighbor. Sieger *et al.* [27] reports very early breakdown of the oscillations during somitogenesis. The interpretation of the apparent disagreement between our results and those in [27] is potentially ambiguous. While our simulated combined knockdown phenotype is more severe than the Her1-only knockdown, our minimalized model did not incorporate *her1*, and thus cannot account for Her1 redundancies and/or interactions.

#### FGF bead grafting

As a further comparison to the simulation results of Cinquin (see Figure 6 and Video S7 in [28]), we simulated the bead grafting experiment of Sawada *et al.* (see Figure 3J–M in [44]). We note that the expression of *her1*, and not *her7*, was examined in this experiement. However, *her7* expression is normally synchronized with *her1* expression along the PSM. In this experiment, the intermediate and anterior-most bands of *her1* mRNA expression were widened and shifted anteriorly by the presence of a bead soaked with FGF, which drives ectopic expression of Her13.2.

Following [28], we assumed that the bead maintained a maximum (saturated) expression of total control protein 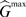 across ten cells, and that the effect was localized to only those cells in direct contact with the bead. As compared to [28], our model exhibited a greater difference in expression amplitude as the level of total control protein 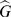 abrubtly changed from 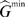 to 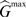 at the bead location, see Figure 7C and Video S9. In Figure K in [44], the intermediate band of *her1* mRNA expression was disrupted at the location of the bead, possibly suggesting a lower expression level there. Our model predicts lower expression of *her7* mRNA, which is usually synchronized with *her1*. Furthermore, our simulated experiment produced a phase shift in the bands of expression as they traveled through the bead area, which agrees with the experimental observations and simulations of Cinquin [28].

## Discussion

We have presented a biologically informed, yet minimally constructed, mathematical model for initiating waves of gene expression in the posterior PSM. Proper and robust spatiotemporal control of the oscillation rate is a key demand on any model that aims to reproduce a biologically realistic clock-wave. By careful model construction and estimation of the relevant model parameters, we identified a combinatorial control mechanism for controlling the oscillation rate of gene expression in PSM cells. As suggested by earlier studies [30],[31],[32], the present work indicates the necessity of more than one binding site for clock protein homodimer self-repression. The present work has further identified that competitive dimerization between the clock protein homodimer and a heterodimer with a spatiotemporally graded control protein can sufficiently slow oscillations and initiate the observed waves of expression in zebrafish, as long as clock protein monomers decay preferentially to dimers. This nonlinear decay mechanism represents an alternative and/or complement to rate-limited decay mechanisms for protein (e.g., Michaelis-Menten kinetics) [33]–[35], and warrants experimental investigation. Our results suggest that there should be an experimentally observable difference in decay rates between clock protein monomers and dimers. This difference would manifest itself in sub-linear dependence of decay on the total protein concentration, see Figure 5 of Text S1.

### Comparison to Existing Models

The modeling and experimental work of Lewis and coworkers in zebrafish [19],[24],[25] was a major foundation for the present work. Compared to their coupled two-cell model, our multicellular model adds explicit tracking of monomer and dimer forms of protein, differential protein decay, and multiple transcription binding sites modeled using the approach of Shea and Ackers [45]. Multiple, active transcription binding sites for the clock-gene *her1* have recently been reported in zebrafish [30]. Our model supports this finding, but also suggests the importance of the differential decay of clock protein monomer and dimer. In spite of the added complexity in our model, a fast dimerization assumption allows it to retain much of the simplicity of Lewis's original deterministic, single clock-gene model [19].

The idea of competitive dimerization of a control protein with clock protein was first introduced by Cinquin [28]. A major difference between our model and Cinquin's model is our inclusion of only a single clock-gene (*her7*). Whereas Cinquin's model suggests the importance of a Her1-Her7 clock protein heterodimer to clock-wave formation in zebrafish, our model reproduces the initiation of the posterior clock-wave with a single clock-gene. Furthermore, in our model the control protein (Her13.2) never acts as a repressor, either as a homodimer or as a heterodimer. However, our parameter sensitivity analysis shows that competitive heterodimerization of clock protein with control protein (Her7-Her13.2) is fundamentally important to the rate tuning mechanism of the model. While the the decay rates for protein monomer and dimer are very similar to each other in Cinquin's model [28], we show that the difference between these rates is largely responsible for tunability of the oscillations.

Buchler *et al.* [29] termed preferential decay of monomers to dimers as “cooperative stability”, and found that it increased the robustness of both a bistable switch and a synthetic oscillator via enlarged parameter regions. More recent work by Wong *et. al* [35] showed that rate-limited protein decay could also enlarge the viable parameter space for an oscillatory genetic circuit. A similar rate-limited protein decay mechanism was identified as potentially playing a positive role in the somitogenesis oscillator in mouse modeled by Zeiser *et al.* [34]. In relation to these results, we see that the effect of differential decay through cooperative stability of dimers is more intricate in our model of the somitogenesis oscillator. While the differential decay *reduces* the parameter region for sustained oscillations as compared to linear decay, it increases the rate-tuning of the oscillator with a changing level of control protein (larger 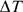), which is crucial to proper clock-wave formation. We note that we only examined the two most extreme cases of differential decay of monomers and dimers, which is almost certainly not what happens *in vivo*. Experimental data on dimer dissociation constants, binding affinities, decay constants, and the quantitative shape and magnitude of the control protein gradient would be particularly useful in further validation, refinement, and application of the presented model.

Finally, although Delta/Notch coupling was not the focus of the present study and no Delta/Notch parameters were estimated during the parameter selection, our robustness studies showed that the synchronization of heterogenous cells in the tailbud is crucial for the proper formation of the clock-wave. While in this paper we assumed relatively weak coupling and mainly explored the interaction between the control protein and the clock protein, a stronger effect of the signaling protein on clock mRNA production could add complexity to this interaction. Both the amplitude and timing of the Notch signal may be important. Because the decreasing level of total control protein along the PSM shifts the clock mRNA production curve, the relative influence of Notch signal on clock mRNA production also changes. It was noted in [19] that increasing the Notch delay can cause two coupled cells oscillating in synchrony to anti-synchronize. While in the present study the Notch delay is fixed, the underlying oscillation rate is changing as a function of the total control protein 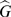. This change in relative timing presents another potential mechanism for Notch coupling to act differently along the AP axis of the PSM.

### Outlook

In the last decade, our understanding of somitogenesis benefited from great experimental advances which identified, in multiple organisms, candidates for both clock- and signaling-genes and various candidates for graded morphogens (control proteins) that may interact with these genes. However, there is still a vigorous discussion about which of the genes are driving the clock and which are driven by the clock, the role of multiple clock genes, how and which morphogens interact with the clock, and how the somite boundaries ultimately form. What can mathematical modeling bring to the table in face of such uncertainty and complexity?

One approach has been to radically simplify the underlying biology and concentrate on just the observed phenomena. As an example, one can model the clock as a phase oscillator and the wavefront as a prescribed decrease in oscillation frequency and see if a viable clock-wave is generated, see [46] for example. Results of these models highlight the essential features necessary for the clock-wave: slowing of the oscillation as the cell matures in the PSM and coordination of oscillations in cohorts of cells with the same fate. These models, however, do not draw conclusions about the biological mechanisms underlying the clock formation.

Our results suggest that a mathematical model can incorporate the existing (incomplete) understanding of biology and still suggest concrete, experimentally refutable hypotheses about the biological mechanisms of somitogenesis clock-wave generation. Although our mathematical model was validated using zebrafish data, our model is readily adaptable to other organisms and we believe that its minimal construction makes it a good candidate for further investigation of the key biological questions.

## Models

Our mathematical model of the clock-wave in the posterior PSM has three components: 1) an oscillating clock-gene in each cell (clock), 2) a spatiotemporally graded control protein that controls the clock's oscillation rate (control protein), and 3) a coupling-signal gene whose protein signal couples oscillations between cells (coupling signal). These components are present in the standard model organisms, including zebrafish, chick, and mouse [1]. We describe these three components in turn.

### 

#### Clock

Motivated by zebrafish models [19],[24],[25], we track both mRNA and protein levels of a single clock-gene. The clock protein can form a homodimer that represses its mRNA production after a delay. This system is capable of autonomous, sustained oscillatory gene expression [20]. The relative amounts of clock protein monomers, homodimers, and heterodimers with the control protein are explicitly tracked, allowing different decay rates for each [29]. Control of clock mRNA transcription is modeled using the approach of Shea and Ackers [45],[47],[48]. This modeling formalism is a significant simplification of the eukaryotic transcription process [49], but represents an initial step towards biological realism as compared to many existing models [28],[31],[47].

#### Control protein

Motivated by [26]–[28], we suppose that the graded control protein interacts with the clock protein by heterodimerization. In contrast to the model of Cinquin [28], heterodimers do not repress production of the clock protein, and homodimerization of the control protein is allowed. It is assumed that neither control protein homodimers nor heterodimers with clock protein can repress clock-gene transcription because the control protein in zebrafish (Her13.2) has a truncated amino acid sequence normally used for DNA binding [26]. The control protein level is prescribed with a maximum value in the tailbud that deceases anteriorly in the PSM. The graded level of control protein, combined with competitive dimerization between control and clock proteins, results in slowing oscillation rates in successively anterior cells and the formation of the clock-wave.

#### Coupling signal

Motivated by the intercellular Delta/Notch signaling pathway in zebrafish [2],[19],[22], [23],[25],[50],[51], we assume that the mRNA of the coupling-signal gene is repressed by the clock protein homodimer and that the clock mRNA is activated by the signaling protein from adjacent cells. Because Notch signaling is non-diffusive and contact-dependent, the effect of the signaling protein is confined to nearest neighbors. Following [25], we assume that the effect of the coupling signal on the clock is an order of magnitude weaker than the clock's self-repression. While likely true for zebrafish [22],[23],[25],[50],[51], this may not be a valid assumption in chick or mouse [1],[8],[51],[52].

### Mathematical Model

#### PSM growth

For the 1D model, we consider a line of 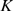 total cells along the anterior-posterior (AP) axis that are assumed to enter the posterior-most PSM from the tailbud at regularly spaced time intervals. 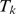 denotes the time of entry of the 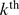 cell into the PSM, and is given by the linear relationship
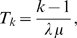
(1)where the constant 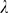 is the number of cells per AP somite length and the constant 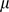 is the somite formation rate in somites per minute, which is equal to the oscillation frequency in the tailbud [1],[2]. The steady growth and oscillation assumptions are a reasonable approximation over a significant portion of developmental time [4],[53].

#### The control protein


**Figure 8:**
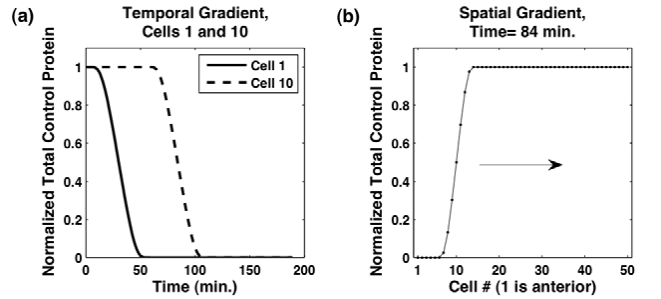
Spatiotemporal control protein gradient. The spatiotemporal gradient of the total control protein is prescribed using the normalized sigmoidal function 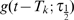 where 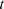 is time, 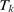 is the time that cell 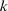 exits the tailbud, and 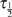 is the half-life. Note that 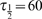 minutes was used in all simulations. (A) Temporal gradient as a function of time in cell one, 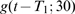, and cell ten, 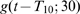. Cell ten, which exits from tailbud later than the cell one, maintains the maximum level of total control protein for a longer time. (B) Spatial gradient as a function of cell position given by 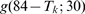. As tailbud growth adds cells to the PSM, the tailbud-PSM boundary moves right, and the spatial gradient profile follows this moving boundary.

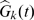
 denotes the amount of total control protein in the nuclear compartment of the 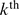 cell at time 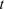. Before cell 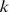 enters the posterior PSM from the tailbud (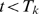), 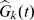 is assumed to be a maximal constant specified by 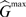. After entering the PSM (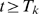), the total control protein is assumed to decrease monotonically to 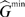 with half-life 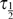. Altogether, 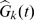 is prescribed by
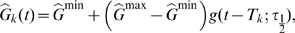
(2)where 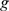 is a normalized sigmoidal function. The resulting spatial profile agrees qualitatively with those computed in [54], see Figure 8. (Text S1 has additional details on the selection of the function 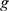.)

#### The intracellular clock

For each cell 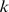 we track the amount of total clock protein, 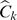, and its mRNA, 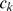, in units of copy number per nuclear and cytosolic compartment, respectively [19]. The total clock protein 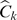 exists in three possible forms: monomer 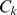, homodimer 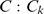, or heterodimer with the control protein 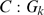. Likewise, the total control protein 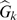 is distributed as monomer 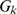, homodimer 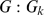, or heterodimer 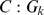. The production rate of the clock protein and mRNA in 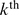 cell is given by two delay differential equations
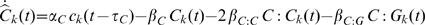
(3)

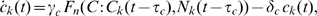
(4)where, following [19],
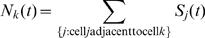
(5)is the total signal from neighboring cells. The function 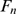 represents the combined effect of the repressor 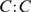 and activator 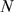 on mRNA production, where 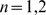 is the number of binding sites for the repressor 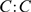. 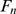 and the dynamics of 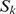 are described below. Greek letters represent positive model parameters: 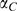 is the production constant for protein (as monomer) from mRNA occurring with delay 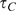. 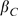, 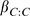, and 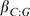 are the decay constants for protein in monomer, homodimer, and heterodimer forms, respectively. 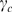 affects the mRNA production rate, which occurs with delay 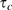. Lastly, 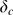 is the mRNA decay constant.

#### Competitive dimerization

The control protein interacts with the clock protein by competitive dimerization, which is assumed to happen on a much faster time scale than the production and decay of protein and mRNA [29],[48]. The conservation laws 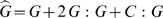 and 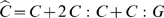 hold at any time in a given cell. Using mass action kinetics and letting 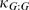, 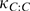, and 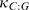 be the dissociation constants of the respective dimers, these equations may be rewritten in terms of total protein and monomer (see Text S1 for details):
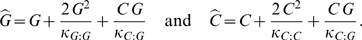
(6)


#### Intercellular signaling

The dynamics of the coordinating signal protein, 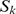, and mRNA, 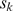, in the 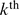 cell are given by equations similar to (3)–(4):
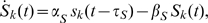
(7)

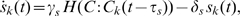
(8)with Greek letters representing the analogous parameters. 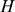 is a function (described below) representing mRNA production in the presence of the repressor 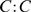. This arrangement was proposed in [19] as a mechanism for synchronizing expression of the clock and coordinating-signal genes, as observed in zebrafish.

#### Gene regulation by transcription factors

The 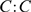 and 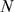 transcription factors are assumed to regulate mRNA production through *cis* binding at a given gene. The binding of these transcription factors to DNA is assumed to be in chemical equilibrium, and the approach of Shea and Ackers [45],[47],[48] is used to derive functions 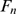 in equation (4) and 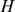 in (8). These functions model the probability that the RNA polymerase II (RNAP-II) transcription complex is assembled on a gene's promoter. Specifically,
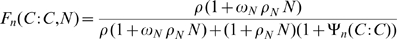
(9)describes the probability that the promoter is in either of two states in which clock mRNA transcription occurs. The unitless parameter 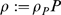 is defined as the product of the binding affinity 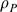 for RNAP-II complex and the copy number 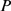 of the complex. 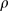 incorporates the assembly of the RNAP-II complex, a process which, in eukaryotes, can involve several intermediate steps (see Figure 6–16 in [55]). 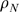 is the promoter binding affinity of the protein 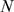 and 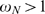 is the cooperativity between the activator 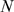 and the RNAP-II complex.

The denominator in (9) represents all possible promoter states. The simplest assumption is that the activator 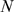 and the repressor 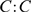 bind independently, allowing the factorization 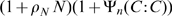. This product represents all the states in which RNAP-II complex is not bound to the promoter. The function 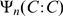 represents the self-repression of the clock mRNA by clock protein dimer, 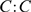, given 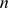 binding sites. For model scenarios with one binding site,
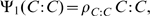
(10)where 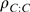 is the binding affinity for the 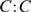 dimer. For model scenarios with two binding sites, with each site assumed equally likely to be bound by 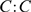 dimer,
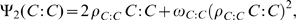
(11)where 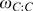 is the cooperativity between two simultaneously bound 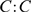 dimers. A more detailed derivation of 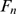 appears in Text S1.

Assuming no activation and one binding site, a similar argument leads to the following form of the function 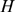 in (8):
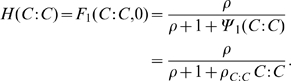
(12)Note that, because of the lack of experimental data, the same repressor binding affinity of clock homodimers, 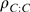, is assumed in both 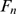 and 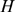, even though these functions represent mRNA production from different genes.

#### Parameter values

Our model has 28 total parameters. Parameters in Table 1 depend upon the model scenario under consideration. Parameters in Table 3 are fixed in all simulations and, with the exception of 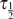, their values are determined experimentally. Table 2 lists ten parameters whose values were estimated from a biologically realistic range (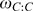 does not apply to model scenarios I and II). From these ranges we searched for parameter values conducive to clock-wave formation, as described next. The last column of each of these tables references the source for each value/range. Parameter value and range selection is described in greater detail in Text S1.

### Model Simulation and Selection

As described in the Results section, model selection occurred in two stages. In each stage, the model was simulated numerically using one of Matlab's delay differential equation solvers (dde23 or ddesd [56]). Because the system of differential equations contained algebraic constraints, each evaluation of the right hand side of the system of delay differential equations by the solver required that the nonlinear algebraic system (6) be solved for the monomer copy numbers 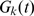 and 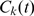 in terms of the prescribed value of 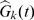 and the state variable 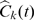. As an alternative to Newton's method, a simple iterative technique for solution of this nonlinear algebraic system was developed (see Text S1 for details). Computation of the dimer copy numbers 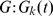, 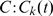, and 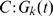 followed from the corresponding fast equilibrium equations. A matlab class (Params.m) was developed to handle the various model configurations and parameter perturbations, ensuring accuracy and reproduction of results. See Text S2 for listings of the Matlab codes employed.

#### Stage one simulations

Stage one simulations were run for two coupled cells using matlab's delay differential equation solver dde23 [56]. In this stage, 40,000 random samples were selected from a uniform joint distribution of the following eight parameters: 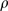, 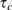, 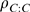, 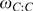, 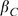, 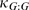, 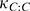, and 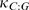, whose ranges are given in Table 2. We selected parameters 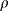, 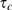, and 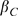 uniformly from their ranges. Because the ranges for 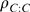, 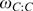, 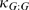, 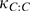, and 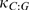 are characterized through a range of powers of 10, these parameters were selected uniformly in the power. As an example, because the range of 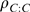 was 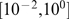, we would make a uniform selection 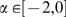 and then set 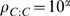. For each selection of parameters, the four model scenarios were simulated by selecting the appropriate values for parameters 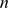, 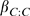, and 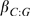 (see Table 1). For each scenario and parameter selection tested, the level of total control protein 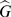 was initially set to zero, and zero initial history functions were used for the state variables 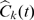, 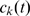, 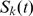, and 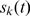 in the system (3)–(4), (7)–(8), 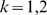. Solutions were allowed to settle to steady-state behavior for at least 250 minutes, and integrations were continued for additional time until a settled solution was detected. The level of total control protein was then incrementally increased in steps of ten until the maximum value of 2500 was reached, with each solution at the next 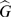 level allowed to settle to steady state behavior for at least 250 minutes (integrating longer if necessary) and using the preceding solution as the history function (for computational efficiency).

If for some level of 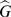 in 0–2500 the steady state behavior was periodic, the parameter set was put into collection 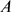. If in addition, for some level of 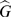, the period was 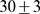 minutes, the parameter set was put into collection 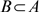. For the parameter sets in 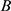, we then found the values of 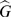 in 0–2500 that produced the largest and smallest period, the difference being 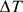. Typically, but not always, the smaller value 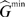 corresponds with the longest period and the larger value, 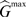 corresponds with the shortest period. The parameter sets in 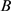 that produced 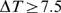 were selected for collection 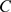, and those that produced 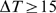 were selected for collection 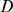. Only scenario III produced a non-empty collection 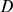. Although there is, a priori, no guarantee that the shortest period will occur at 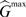 and that this period will fall in the 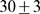 minute range, for all parameter sets in 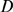, as well as for the best (i.e. the longest 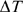) parameter set for scenario IV in collection C, both of these statements are true.

For additional efficiency, solutions for each parameter selection were computed in parallel using matlab's parallel for loop construct parfor, and Newton's method was used to solve the nonlinear algebraic system (6). Statistical analysis of solutions (in particular, computation of the 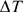 distribution for each model scenario) was also done using matlab. To minimize possible stochastic effects of small copy number not considered by our deterministic model, we accepted only those periodic solutions with sufficiently large amplitudes in each state variable (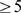 copies, peak-to-peak).

#### Stage two simulations

Stage two simulations verified clock-wave formation of the model scenarios selected/rejected in stage one. 1D simulations consisted of a line of fifty coupled cells representing the AP axis of the PSM and tailbud, while 2D simulations consisted of five parallel lines of fifty cells in a rectangular array. Cells were coupled to their nearest neighbors, which included diagonal cells that meet only at a corner point in 2D simulations. Zero initial history functions were used for the state variables 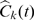, 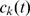, 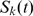, and 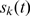 in the system (3)–(4), (7)–(8), and solutions were allowed to settle to steady-state behavior for 500 minutes with the maximum level of total control protein (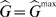). All cells were treated as being in the tailbud during the settling period. After the settling period, cells entered the PSM from the tailbud according to (1), and the level of 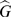 in each cell in the PSM decreased according to (2). In 2D simulations, laterally adjacent cells entered the PSM together and experience the same total control protein gradient as the 1D line of cells.

By visually inspecting 1D and 2D simulations with matlab's dde23 [56], the parameter selections for each model scenario that produced the maximum 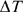 were examined for proper clock-wave generation, verifying that the model III was the only one producing a proper clock-wave. The robustness of model III to a heterogeneous cell population was then examined by perturbing all positive parameters in each cell from the nominal values. The noise added to each parameter was normally distributed (with a non-negativity constraint) so that, on average, 99.7% of the values were within 1% or 2.5% of the nominal values. Because of algorithm efficiency issues in systems with many delays, sdesd was used instead of ode23 in simulations of heterogeneous cell populations. The consistency of output from ode23 and sdesd was also verified using homogeneous cell populations.

## Supporting Information

Text S1 Supporting text explains details of the mathematical model and parameter selection
(0.43 MB PDF)

Text S2 Computer Matlab code used in simulations. The code is available upon request from the authors.
(0.50 MB PDF)

Video S1 Simulated clock-wave in fifty homogeneous cells for model III parameter selections as in paper Figure 2a. Red bars represent cells in the tailbud. Green bars represent cells that have entered the PSM. All cells have identical parameters.
(10.11 MB MOV)

Video S2 Simulated clock-wave in fifty homogeneous cells for model IV parameter selections giving as in paper Figure 2b. Red bars represent cells in the tailbud. Green bars represent cells that have entered the PSM. All cells have identical parameters.
(10.64 MB MOV)

Video S3 Simulated clock-wave in fifty heterogeneous cells for model III parameter selection as in paper Figure 2c. Red bars represent cells in the tailbud. Green bars represent cells that have entered the PSM. Normally distributed noise was applied independently to parameters in all cells, with a positivity constraint and so that 99.7% of the values are within 1% of the nominal values.
(10.45 MB MOV)

Video S4 Simulated clock-wave in fifty heterogeneous cells for model III parameter selection as in paper Figure 2d. Red bars represent cells in the tailbud. Green bars represent cells that have entered the PSM. Normally distributed noise was applied independently to parameters in all cells, with a positivity constraint and so that 99.7% of the values are within 2.5% of the nominal values.
(10.64 MB MOV)

Video S5 Simulated clock-wave in a rectangular array of 250 heterogeneous cells. Simulated clock-wave in a rectangular array of fifty axial by five lateral heterogeneous cells, for model III parameter selection. Darker grey indicates a higher expression level. Interior, edge, and corner cells are coupled to their eight, five, and three adjacent nearest neighbors, respectively. Normally distributed noise was applied independently to parameters in all cells, with a positivity constraint and so that 99.7% of the values are within 1% of the nominal values.
(7.90 MB MOV)

Video S6 Simulated clock-wave in a rectangular array of 250 heterogeneous cells. Simulated clock-wave in a rectangular array of fifty axial by five lateral heterogeneous cells, for model III parameter selection. Darker grey indicates a higher expression level. Interior, edge, and corner cells are coupled to their eight, five, and three adjacent nearest neighbors, respectively. Normally distributed noise was applied independently to parameters in all cells, with a positivity constraint and so that 99.7% of the values are within 2.5% of the nominal values.
(8.52 MB MOV)

Video S7 Replication of Her1 and Her7 protein knockdown experiment. Simulated clock-wave in fifty identical cells for model III parameter selection, except for a 99.9% reduction in the Her7 clock protein production rate. Red bars represent cells in the tailbud. Green bars represent cells that have entered the PSM.
(10.12 MB MOV)

Video S8 Replication of Her1 and Her13.2 protein knockdown experiment. Simulated clock-wave in fifty identical cells for model III parameter selection, except the value of the control protein G_max_ was set to 1% of its regular value. Red bars represent cells in the tailbud. Green bars represent cells that have entered the PSM.
(9.29 MB MOV)

Video S9 Replication of FGF bead grafting experiment. Simulated clock-wave in fifty identical cells for model III parameter selection. We assume that the bead maintained a maximum (saturated) expression of total control protein G_max_ across ten cells, and that the effect was localized to only those cells in direct contact with the bead. Red bars represent cells in the tailbud. Green bars represent cells that have entered the PSM.
(10.20 MB MOV)
