# Supplementary material for: Somitogenesis Clock-Wave Initiation Requires Differential Decay and Multiple Binding Sites for Clock Protein
Source: PLoS Comput Biol. 2010 Apr 1;6(4):e1000728. doi: 10.1371/journal.pcbi.1000728 (PMC2848544; doi:10.1371/journal.pcbi.1000728)
Supplement: Text S1 — Supporting text explains details of the mathematical model and parameter selection (0.43 MB PDF) [file pcbi.1000728.s001.pdf]

SUPPORTING TEXT 1

SOMITOGENESIS CLOCK-WAVE INITIATION REQUIRES DIFFERENTIAL DECAY AND MULTIPLE BINDING SITES FOR CLOCK PROTEIN.

MARK CAMPANELLI AND TOMÁŠ GEDEON

1. Introduction

This supporting information contains five sections with details on the mathematical model, production and decay curves for the clock-gene, parameter value/range selection, parameter estimation, and parameter sensitivity. In the section on parameter selection, we describe how we selected fixed parameter values, or, in the case of ten parameters, biologically realistic ranges for parameter estimation purposes, followed by a short section that provides a tabulation of parameter estimation results. We also give additional information on the sensitivity of the estimated parameters. In the section on the mathematical model, we provide details about modeling the control protein gradient, about computation of the proportion of monomers, homodimers, and heterodimers in an equilibrated mixture of clock protein and control protein, and about the Shea-Ackers derivation of the mRNA transcription regulatory functions. In the section on production and decay curves for the clock-gene, we give representative curves for clock mRNA production and total protein decay.

2. Parameter Values and Parameter Range Selection

We present first all parameters whose values are fixed in our model and the reasons for our choice of these values. We then specify ranges for parameters which, to the best of our knowledge, have not been determined experimentally. From these ranges we search for parameter values conducive to clock-wave formation. Note that copy numbers per compartment can be converted to molar concentration using a nuclear volume $V_{\text{nuc}} = \frac{4}{3}\pi\,(2.5\ \mu\text{m})^3 = 65.4\ \mu\text{m}^3$, and a cytosolic volume $V_{\text{cyt}} = \frac{4}{3}\pi\left((5\ \mu\text{m})^3 - (2.5\ \mu\text{m})^3\right) = 458\ \mu\text{m}^3$, where the nuclear diameter is 5 μm [1] and the cell diameter is assumed to be 10 μm.

2.1. **Fixed parameters.**

λ The number of cells per anterior-to-posterior somite length. While this number is not strictly constant across the PSM in zebrafish, the constant value $\lambda = 5$ cells per somite is a reasonable approximation over a good portion of the tissue [2].

μ The somite formation rate in somites per minute in the anterior PSM. While the formation rate is not strictly constant throughout somite development in zebrafish, μ is roughly constant over a good portion of developmental time [3,4]. The somite formation rate is observed to be equal to the frequency of oscillation in the tailbud [2]. In all clock-wave simulations, an initial settling time occurs in which oscillations stabilize before any cells enter the PSM, which correlates with the initiation of synchronized oscillatory expression in the early embryo before somitogenesis begins. After the settling time, the oscillation period, T, is computed and this computed period is used to determine $\mu = \frac{1}{T}$. μ determines the exit times T_k of cells from the tailbud using the following relationship:

$$T_k = \frac{k-1}{\lambda\,\mu}. \tag{1}$$

In zebrafish, the oscillatory gene expression in the tailbud has a period of 30 minutes at 28°C [2], so at this temperature $\mu = \frac{1}{30}$ somites per minute. If for any reason the oscillation period cannot be computed after the settling time, the default value $\mu = \frac{1}{30}$ somites per minute is used.

$\tau_{\frac{1}{2}}$ The half-life, in minutes, of the total control protein in a given cell. The half-life is the time after a cell leaves the tailbud when the level of total nuclear protein is reduced to $\frac{1}{2}\,\widehat{G}^{\max}$. To the best of our knowledge, quantitative data for this parameter for the control protein Her13.2 in zebrafish are unavailable. Simulations have shown that a range of values is typically acceptable, e.g., 45–75 minutes. The choice of $\tau_{\frac{1}{2}}$ can affect both the initial shape and axial positioning of the clock-wave. All simulations in this work use $\tau_{\frac{1}{2}} = 60$ minutes, so that the half-life is not too short in relation to the oscillation period in the zebrafish tailbud.

τ_C The delay, in minutes, arising from clock protein production at the ribosomes and subsequent transport into the nucleus. The value $\tau_C = 1.7$ minutes from [1] is used in all simulations.

α_C, α_S The production constants, in protein copies per mRNA copy per minute, of clock and signaling protein, respectively. The common value $\alpha_C = \alpha_S = 4.5$ protein copies per mRNA copy per minute from [1] is used in all simulations except the Her7 knockdown experiment.

γ_c, γ_s Production constants, in mRNA copies per minute, of clock and signaling mRNA, respectively. $\gamma_c = \gamma_s$ in all simulations, and the common value is computed from the chosen value of ρ so that the baseline mRNA production rate in the absence of repressors or activators is the following value cited in [1]:

$$\gamma_c\left(\frac{\rho}{\rho+1}\right) = \gamma_s\left(\frac{\rho}{\rho+1}\right) = 33 \text{ mRNA copies/minute.} \tag{2}$$

ρ_N The promoter binding affinity of the activator N, with units of inverse copy number. To the best of our knowledge, this constant has not been measured in zebrafish. In the generation of oscillatory gene expression in zebrafish, the intercellular signaling does not dominate the intracellular self-repression of the clock-gene [5]. Following the critical concentration parameters used in [6], the activator N is given one tenth the binding affinity of the repressor $C{:}C$ in all simulations, i.e., $\rho_N = \frac{\rho_{C:C}}{10}$.

ω_N The unitless cooperativity between simultaneously bound activator N and RNAP-II complex at the promoter. This number is strictly greater than one. To the best of our knowledge, this parameter has not been measured in zebrafish, so a typical value $\omega_N = 25$ is used in all simulations, which is taken from [7].

δ_c The decay constant, in inverse minutes, of clock mRNA. In all simulations the value used is $\delta_c = 0.206$ minute^{-1} from [6], which is estimated from experiments on zebrafish [5].

τ_S The delay, in minutes, arising from coordinating signal protein production at the ribosomes and subsequent transport through the cell membrane into the nuclei of adjacent cells. The value $\tau_S = 20$ minutes from [1] is used in all simulations.

β_S The decay constant, in inverse minutes, of coordinating signal protein. The value $\beta_S = 0.23$ minute^{-1} from [1] is used in all simulations.

τ_s The delay, in minutes, arising from coordinating signal mRNA transcription at the DNA, post-transcriptional processing, and subsequent transport into the cytosol. The value $\tau_s = 12.4$ minutes from [6] is used in all simulations. This value was taken from a experimentally determined range for zebrafish, which was obtained as a sum of a (non-measured) initiation delay that is assumed to be in the range of 3 to 8 minutes in [5]. This initiation delay is added to the experimentally determined range of 8.4 ± 1.2 minutes. In [6] the initiation delay is taken to be 4 minutes resulting in the value $8.4 + 4 = 12.4$ minutes. Using the full range of initiation delays and full range of measured delays gives the range $[7.2+3, 9.6+8] = [10.2, 17.6]$ minutes.

δ_s The decay constant, in inverse minutes, of coordinating signal mRNA. In all simulations the value used is $\delta_s = 0.273$ minute^{-1} from [6], which is estimated from experiments on zebrafish [5].

2.2. **Parameters Estimated from a Range of Values.**

$\widehat{G}^{\min}$ The minimum level of total control protein, which occurs in the intermediate PSM and has units of copy number per nucleus. In zebrafish, this parameter represents the minimum level of total nuclear Her13.2 protein. To the best of our knowledge, quantitative data for this parameter in zebrafish are unavailable, so $\widehat{G}^{\min}$ is estimated from the range 0–2500 copies per nucleus.

$\widehat{G}^{\max}$ The maximum level of total control protein, which occurs in the tailbud and posterior-most PSM and has units of copy number per nucleus. In zebrafish, this parameter represents the maximum level of total nuclear Her13.2 protein. To the best of our knowledge, quantitative data for this parameter in zebrafish are unavailable, so $\widehat{G}^{\max}$ is estimated from the range 0–2500 copies per nucleus. This allows the maximum control protein level to be on the same order of magnitude as the typical mean level of the oscillatory total clock protein.

ρ The unitless product of the binding affinity ρ_P for RNAP-II complex to a gene's promoter and the copy number P of the complex, i.e., $\rho := \rho_P P$. The assembly of the RNAP-II complex is assumed to be a fast process at equilibrium so that P, and therefore ρ, may be treated as constant. To the best of our knowledge, data for zebrafish are unavailable, so ρ is estimated from the range $\frac{1}{3} - 3$, which can be interpreted as a $25\% - 75\%$ probability of the complex binding in the absence of any other repressors or activators. Note that the lumped parameter ρ may include effects of several constitutively expressed transcription factors in the PSM, such as DeltaD and Su(H) in zebrafish [8, 9].

τ_c The delay, in minutes, arising from clock mRNA transcription at the DNA, post-transcriptional processing, and subsequent transport into the cytosol. Previous work [1] has shown that the existence and period of sustained oscillations is sensitive to the total system delay. Because of this sensitivity, τ_c is estimated from the range $2.3 - 8.1$ minutes. This range is derived from the experiments in [5], which estimated $\tau_c = 3.7 \pm 1.4 + T_{\text{init}}$ minutes, where T_{init} is an unmeasured initiation delay assumed to be in the range of 3 to 8 minutes. Because of differences in the present model from the model in [6], T_{init} was taken to be in the range $0 - 3$ minutes, giving the minimum value $3.7 - 1.4 + 0 = 2.3$ minutes, and the maximum value $3.7 + 1.4 + 3 = 8.1$ minutes. Subsequent analysis of the parameter sensitivities verified that this is an adequate range for modeling oscillations in zebrafish. See Figures 1 and 3.

$\rho_{C:C}$ The binding affinity of the $C{:}C$ repressor to a gene's promoter, with units of inverse copy number. To the best of our knowledge, this constant has not been measured in zebrafish, so $\rho_{C:C}$ is estimated from the biologically realistic range $0.01 - 1$, which is taken from the range given in [7], and which includes the (roughly equivalent) critical concentration used in [1].

$\omega_{C:C}$ The unitless cooperativity between two $C{:}C$ repressors simultaneously bound to a gene's promoter. To the best of our knowledge, this constant has not been measured in zebrafish, so $\omega_{C:C}$ is estimated from the biologically realistic range $1 - 100$ taken from [7]. The value of this parameter is irrelevant for a single binding site, i.e., when $n = 1$.

β_C The decay constant, in inverse minutes, of clock protein monomer. Previous work [1, 10] has shown that the existence and period of sustained oscillations is sensitive to the protein decay rate. Because of this sensitivity and the fact that the present model explicitly tracks the distribution of total clock protein as monomer, homodimer, and heterodimer, β_C is estimated from the range 0.2 – 0.5 minute^{-1}, which includes the value 0.23 minute^{-1} from [1] corresponding to a protein monomer half-life of $\frac{\ln 2}{0.23} = 3$ minutes.

$\kappa_{G:G}, \kappa_{C:C}, \kappa_{C:G}$ The protein dimer dissociation constants with units of copy number. To the best of our knowledge, values for these constants have not been measured in zebrafish, so each dissociation constant is independently estimated from the range 10 – 1000 copies, which is based on the range cited in [11].

2.3. Parameters for Model Scenarios I–IV.

n The number of binding sites for C:C repressor at the clock-gene's promoter. Previous work [12] has shown that the number of binding sites can be a key parameter affecting oscillations produced through delayed negative feedback. In model scenarios I and II, $n = 1$ corresponds to a single binding site. In model scenarios III and IV, $n = 2$ corresponds to two binding sites.

$\beta_{C:C}, \beta_{C:G}$ The respective decay constants, in inverse minutes, of clock protein homodimer and heterodimer with control protein. In all simulations, these parameters share a common value, i.e., $\beta_{C:C} = \beta_{C:G}$. Attention to the findings in [11], concerning differential decay of monomers and dimers, suggests consideration of two limiting cases. In model scenarios I and III, $\beta_{C:C} = \beta_{C:G} = 0$ corresponds to monomer only clock protein decay and thus nonlinear decay of total clock protein. In model scenarios II and IV, $\beta_{C:C} = \beta_{C:G} = \beta_C$ corresponds to equivalent decay of all clock protein forms and gives linear (i.e., first order) decay of total clock protein.

3. Parameter Estimation Tabulations

3.1. Tabulations of ΔT, main article Figure 1B. For model validation stage one, the following table gives the number of simulated solutions out of 40,000 total simulations that had an oscillatory solution with period 30 ± 3 minutes for some level of $\widehat{G} \in [0, 2500]$ and had ΔT in the given range. Note that $\Delta T \geq 15$ minutes is needed for formation of a realistic posterior clock-wave.

Scen. \ ΔT Int.	[0, 2.5)	[2.5, 5)	[5, 7.5)	[7.5, 10)	[10, 12.5)	[12.5, 15)	[15, 17.5)
III	1267	1050	584	229	77	32	8
IV	10364	3875	1139	129	2	0	0

4. Sensitivity of Estimated Parameters in scenarios III and IV

Recall that we selected parameters ρ, τ_c, and β_C uniformly from their ranges, but since the ranges for $\rho_{C:C}$, $\omega_{C:C}$, $\kappa_{G:G}$, $\kappa_{C:C}$, and $\kappa_{C:G}$ are characterized through the range of

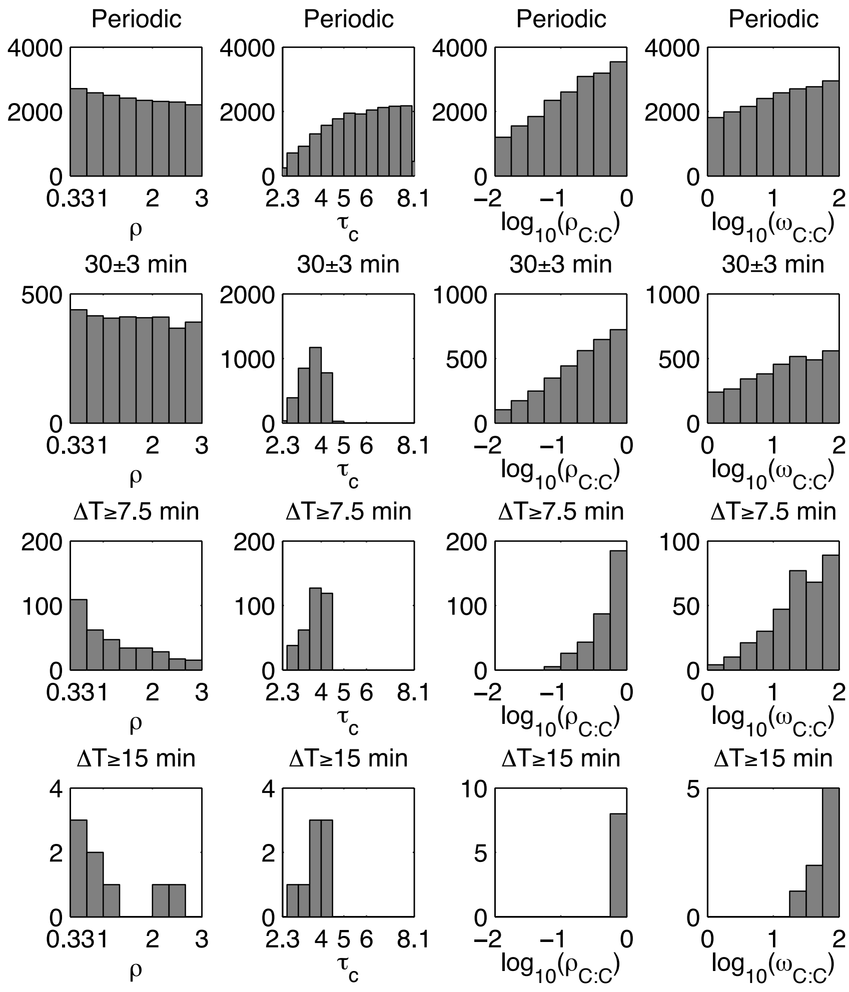

FIGURE 1. **Parameter sensitivities for model scenario III, part 1.** ρ, τ_c, $\rho_{C:C}$, and $\omega_{C:C}$. Graphs give the number of simulated solutions out of 40,000 total simulations satisfying the specified criteria. All parameters are randomly selected from distributions whose ranges are given by the x-axis limits in a way described in the text.

powers of 10, for these parameters we selected uniformly the power. These histograms reflect the sensitivity of clock-wave formation to individual parameters in two ways. Comparing the bottom row of the Figures the sensitive parameters have narrow distributions,

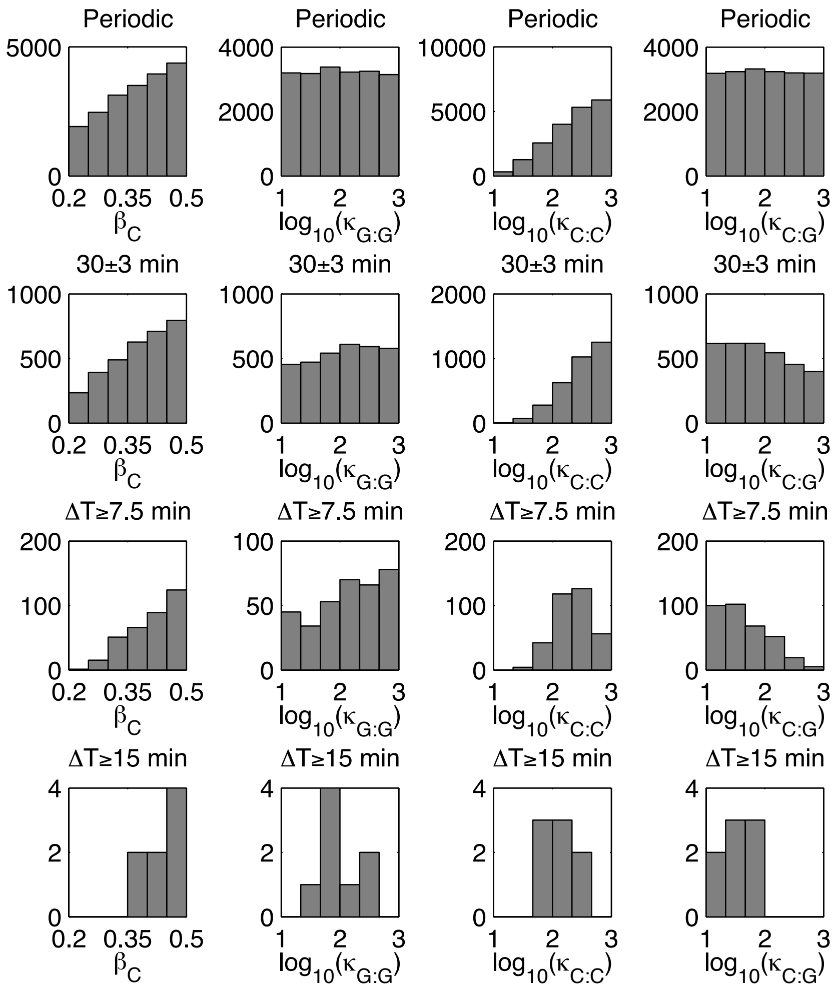

Figure 2. **Parameter sensitivities for model scenario III, part 2.** β_C, $\kappa_{G:G}$, $\kappa_{C:C}$, and $\kappa_{C:G}$. Graphs give the number of simulated solutions out of 40,000 total simulations satisfying the specified criteria.All parameters are randomly selected from distributions whose ranges are given by the x-axis limits in a way described in the text.

which reflect the need for a precise value of this parameter for proper clock-wave formation, while insensitive parameters have wide distributions. On the other hand, comparing histograms along the columns of the Figures 1 and 2 show the selective strength of the four

requirements on each parameter. We summarize the data for the most interesting data selection processes in scenario III and scenario IV, see Figures 1, 2, 3 and 4. In scenario III attaining the proper period of oscillation (30 ± 3) was most sensitive to the clock mRNA production delay τ_c. Furthermore, attaining sufficiently large ΔT for clock-wave formation was most sensitive to the clock homodimer binding affinity $\rho_{C:C}$ and cooperativity $\omega_{C:C}$, the clock monomer decay rate β_C, and the dimer dissociation constants $\kappa_{G:G}$, $\kappa_{C:C}$, and $\kappa_{C:G}$. While both scenarios showed sensitivity of the dimer dissociation constants to increasing ΔT, scenario IV showed additional sensitivity to the heterodimer dissociation constant $\kappa_{C:G}$.

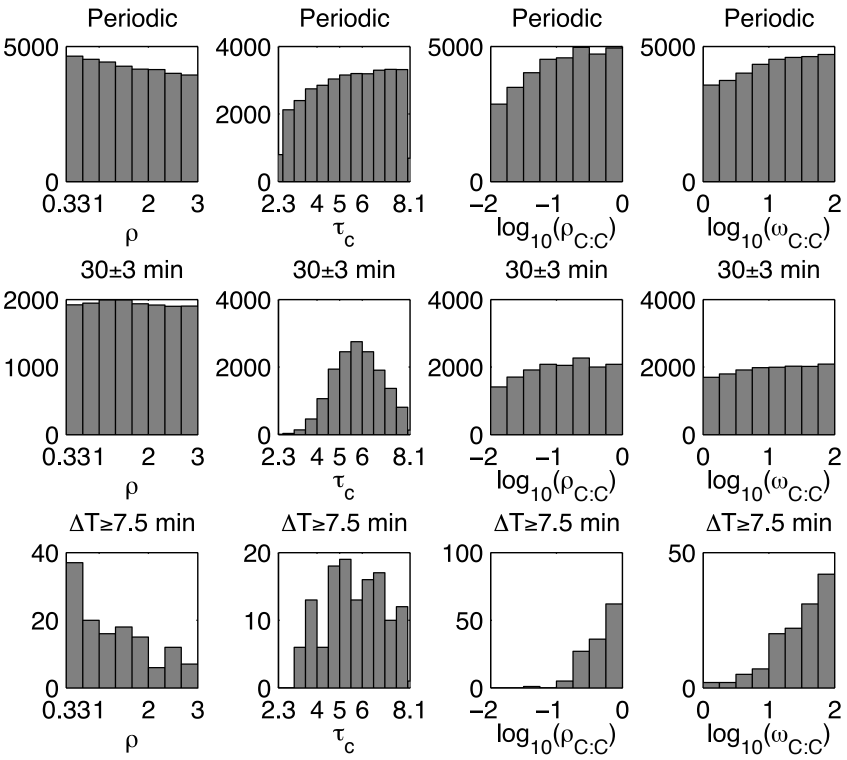

FIGURE 3. **Parameter sensitivities for model scenario IV, part 1.** ρ, τ_c, $\rho_{C:C}$, and $\omega_{C:C}$. Graphs give the number of simulated solutions out of 40,000 total simulations satisfying the specified criteria. All parameters are randomly selected from distributions whose ranges are given by the x-axis limits in a way described in the text.

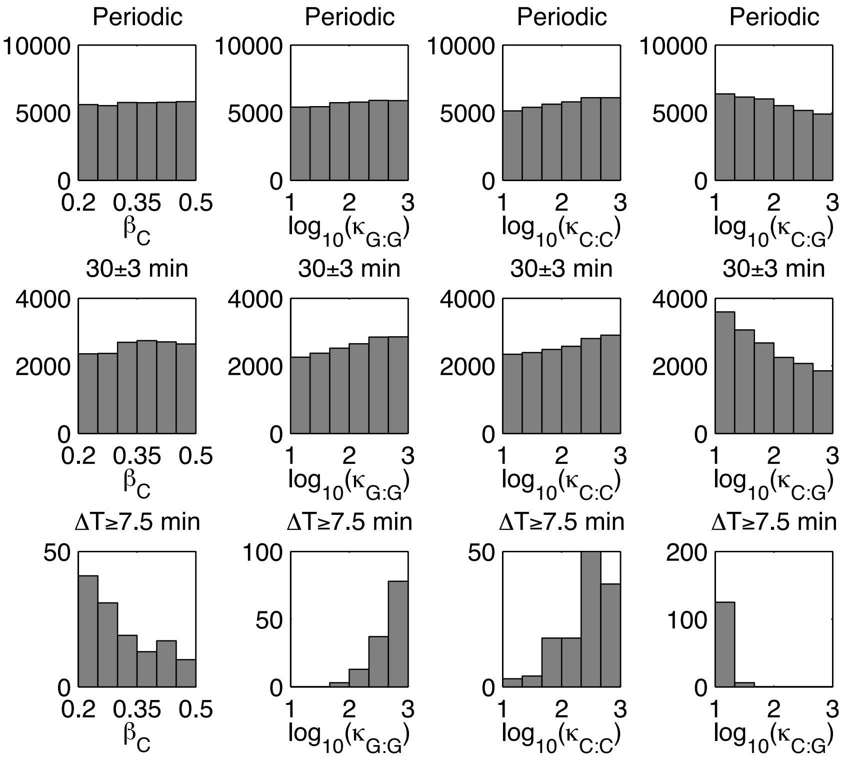

FIGURE 4. **Parameter sensitivities for model scenario IV, part 2.** β_C, $\kappa_{G:G}$, $\kappa_{C:C}$, and $\kappa_{C:G}$. Graphs give the number of simulated solutions out of 40,000 total simulations satisfying the specified criteria. All parameters are randomly selected from distributions whose ranges are given by the x-axis limits in a way described in the text.

5. Production and Decay Curves for the Clock-Gene

In the main text we argue that the differential decay of monomers and dimers (cooperative stability) and two binding sites for the repressor dimers combine to produce large ΔT and a significant change in oscillation rate along the PSM. One of the effects can be seen in Figure 5 where we compare the linear decay rate (scenarios II and IV) and the differential decay rate (scenarios I and III) as a function of the level of total clock protein. Because the proportion of dimers to monomers increases with the level of total clock protein, and only monomers decay under differential decay, the marginal decay rate decreases with the level of total clock protein and the overall decay rate is always below the corresponding linear rate. The level of total control protein $\widehat{G}$ has also some effect on the differential decay rate

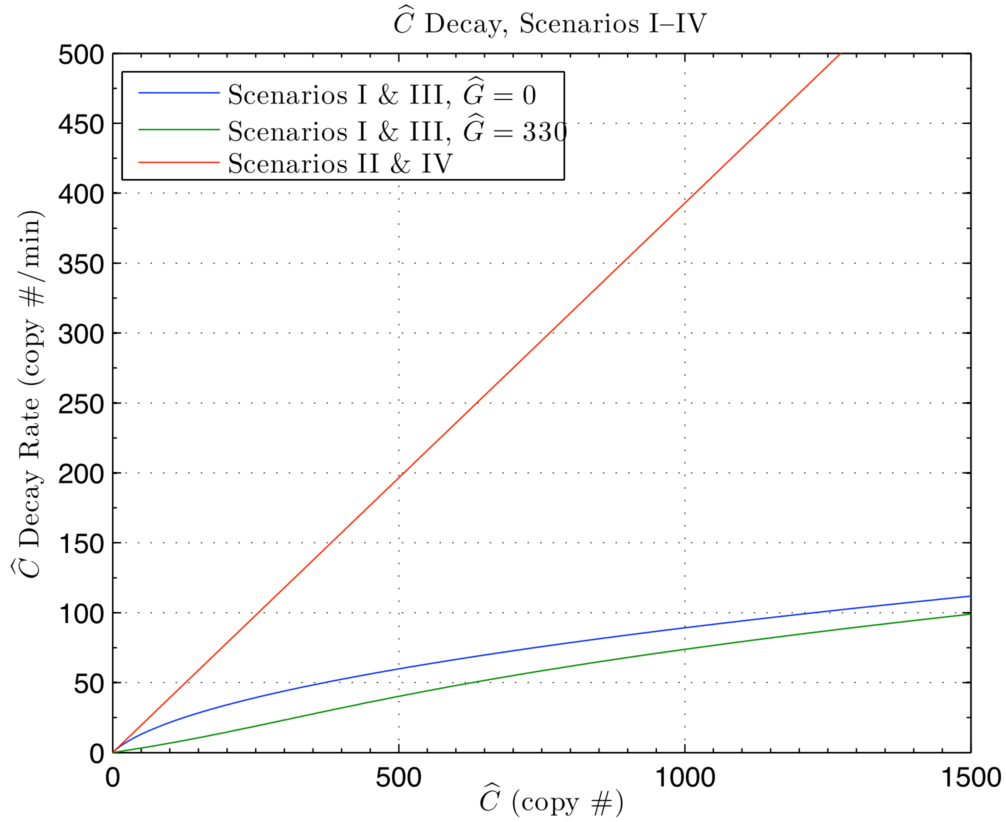

Figure 5. **Total clock protein decay curves for model scenarios I–IV.** In scenarios I and III, total clock protein $\widehat{C}$ decay as a function of $\widehat{C}$ is nonlinear and depends on the level of total control protein $\widehat{G}$. In scenarios II and IV, total clock protein $\widehat{C}$ decay is a linear function of $\widehat{C}$ and is independent of the level of total control protein $\widehat{G}$. For comparison the optimal parameter set for scenario III is used.

(but not on the linear decay rate), because in the tailbud $\widehat{G} = 330$ and a certain portion of clock protein is sequestered as heterodimer with control protein (see Figure 3B of the main paper.)

The two binding sites primarily affected the production of the clock mRNA, since they increased the effective Hill coefficient of the nonlinearity. Figure 6 compares the nonlinear production curve of clock mRNA as a function of total clock protein level. The production curves for scenarios III and IV (two binding sites) were shifted toward low levels of total clock protein as compared to production curves for scenarios I and II (single binding site). Observe that the production curve is shifted even lower as a function of decreasing $\widehat{G}$.

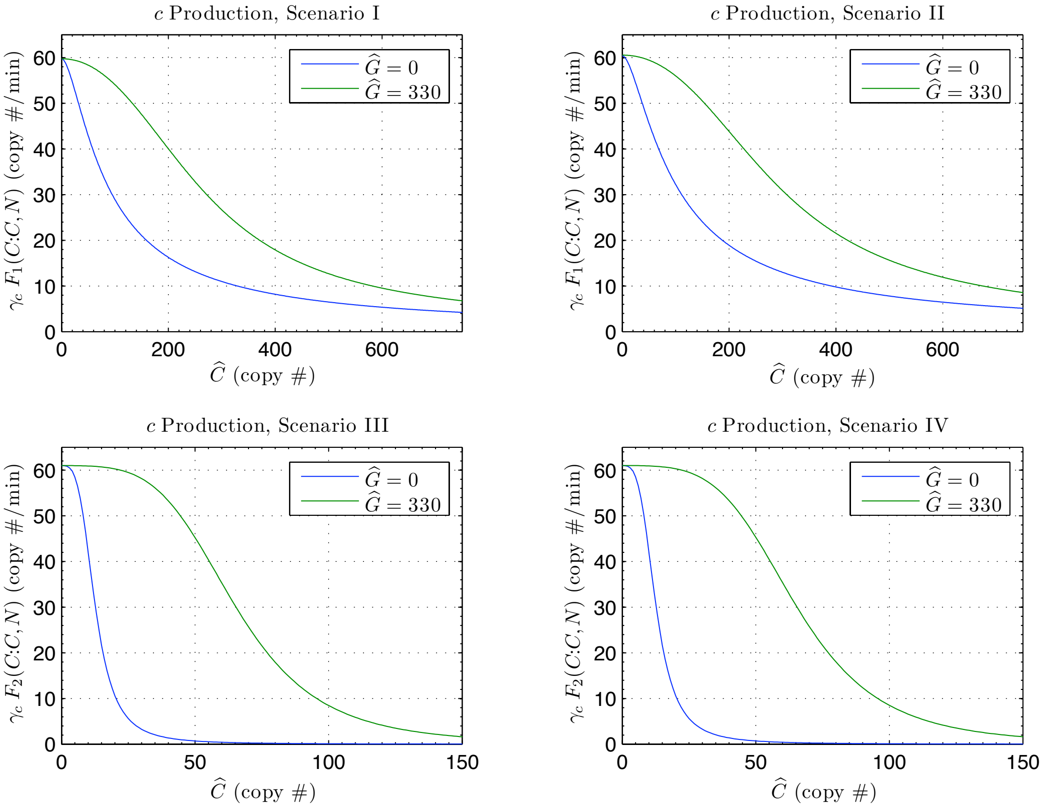

Figure 6. **Clock mRNA production curves for model scenarios I–IV.** Clock mRNA c production as a function of $\widehat{C}$. In scenario I, $N = 18$ for $\widehat{G} = 0$, $N = 18$ for $\widehat{G} = 330$. In scenario II, $N = 36$ for $\widehat{G} = 0$, $N = 48$ for $\widehat{G} = 330$. In scenario III, $N = 520$ for $\widehat{G} = 0$, $N = 497$ for $\widehat{G} = 330$. In scenario IV, $N = 631$ for $\widehat{G} = 0$, $N = 707$ for $\widehat{G} = 330$. For comparison the optimal parameter set for scenario III is used.

Finally, note different scale on the x-axis in scenario I and II figures and scenario III and IV figures, which shows the effect of multiple binding sites on the nonlinearity of the production curves.

6. Mathematical Model

6.1. The Control Protein. In validation stage two, the level of total control protein in the kth axial cell was prescribed by $\widehat{G}_k(t) = \widehat{G}^{\min} + \left(\widehat{G}^{\max} - \widehat{G}^{\min}\right) g(t - T_k; \tau_{\frac{1}{2}})$, where g

is the following continuously differentiable function of time:

$$g(t;\tau_{\frac{1}{2}}) = \begin{cases} 1, & t \leq 0, \\ 1-\frac{1}{2}\,e^{2\left(t-\tau_{\frac{1}{2}}\right)\big/ t}, & 0 < t \leq \tau_{\frac{1}{2}}, \\ \frac{1}{2}\,e^{2\left(t-\tau_{\frac{1}{2}}\right)\big/\left(t-2\,\tau_{\frac{1}{2}}\right)}, & \tau_{\frac{1}{2}} \leq t < 2\,\tau_{\frac{1}{2}}, \\ 0, & 2\,\tau_{\frac{1}{2}} \leq t, \end{cases} \tag{3}$$

where $\tau_{\frac{1}{2}} > 0$ is the half-life parameter. The function g is sigmoidal, concave down for $0 < t < \tau_{\frac{1}{2}}$, and concave up for $\tau_{\frac{1}{2}} < t < 2\,\tau_{\frac{1}{2}}$, see Figure 8 in the main article. T_k is the time that cell k enters the PSM, given by equation (1) above.

Our simulation work indicated that clock-wave formation is more sensitive to the size of the period change ΔT than the half-life $\tau_{\frac{1}{2}}$ of the total control protein. We tested values of $\tau_{\frac{1}{2}} \in [10, 120]$ minutes. The shortest $\tau_{\frac{1}{2}}$ values produced a disorganized wave because the control protein changed too quickly, while longer half-lives shifted generation of the clock-wave anteriorly, even though the clock-wave still formed. All stage two simulations described in this work were run with $\tau_{\frac{1}{2}} = 60$ minutes. The precise shape of the gradient function $g(t, \tau_{\frac{1}{2}})$ was not found to be critical; for example, for the optimal scenario III solution, an exponential decay of the total control protein with $\tau_{\frac{1}{2}} = 60$ minutes also generated a reasonably good clock-wave. The shape of g was chosen to qualitatively match the profiles computed in [13].

6.2. **Competitive Dimerization.** Given the amount of total control protein $\widehat{G}$ and total clock protein $\widehat{C}$ in a cell's nucleus at any given time, we compute the relative amounts of protein monomers, homodimers, and heterodimers. Equilibration of all protein monomers and dimers is assumed to be very fast relative to production and decay of protein and mRNA [11, 14]. The following relationships between total protein, monomer, and dimers hold for control and clock protein, respectively:

$$\widehat{G} = G + 2\,G{:}G + C{:}G, \qquad \widehat{C} = C + 2\,C{:}C + C{:}G. \tag{4}$$

There are three possible dimerization reactions:

$$2\,G \underset{k^-_{G:G}}{\overset{k^+_{G:G}}{\rightleftharpoons}} G{:}G, \qquad 2\,C \underset{k^-_{C:C}}{\overset{k^+_{C:C}}{\rightleftharpoons}} C{:}C, \quad \text{and} \quad C+G \underset{k^-_{C:G}}{\overset{k^+_{C:G}}{\rightleftharpoons}} C{:}G,$$

where the forward and reverse (positive) reaction rates are given above and below the arrows, respectively. Mass action kinetics at equilibrium leads to the following dimerization equations:

$$G{:}G = \frac{G^2}{\kappa_{G:G}}, \quad C{:}C = \frac{C^2}{\kappa_{C:C}}, \quad C{:}G = \frac{C\,G}{\kappa_{C:G}}, \tag{5}$$

where $\kappa_{G:G} := \frac{k^-_{G:G}}{k^+_{G:G}}$, $\kappa_{C:C} := \frac{k^-_{C:C}}{k^+_{C:C}}$, and $\kappa_{C:G} := \frac{k^-_{C:G}}{k^+_{C:G}}$ are the respective dimer dissociation constants. Equations (5) can be used to replace dimers with monomers in equations (4), giving

$$\begin{aligned} \widehat{G} &= G + \frac{2\,G^2}{\kappa_{G:G}} + \frac{C\,G}{\kappa_{C:G}}, \\ \widehat{C} &= C + \frac{2\,C^2}{\kappa_{C:C}} + \frac{C\,G}{\kappa_{C:G}}. \end{aligned} \tag{6}$$

We rewrite (6) as quadratic equations in G and C, respectively, giving

$$\begin{aligned} 0 &= \frac{2}{\kappa_{G:G}}\,G^2 + \left(1 + \frac{C}{\kappa_{C:G}}\right) G - \widehat{G}, \\ 0 &= \frac{2}{\kappa_{C:C}}\,C^2 + \left(1 + \frac{G}{\kappa_{C:G}}\right) C - \widehat{C}. \end{aligned} \tag{7}$$

We have implemented two methods to solve this system of equations. In our two-cell simulations we have used Newton's method with quadratic convergence. However, Newton's method is more difficult to vectorize in MATLAB for the general case of N cells, and computations can be slowed down by the use of `for` loops and the computation of the inverse Jacobian. Therefore for this case we have implemented an iterative formula, described below, which is slower (typically a linear convergence rate), but it is easy to vectorize in MATLAB.

To describe the iterative scheme, we start by solving equations (7) for non-negative roots G and C, respectively. This gives

$$\begin{aligned} G &= \frac{1}{4}\left(\sqrt{\left(\kappa_{G:G}\left(1 + \frac{C}{\kappa_{C:G}}\right)\right)^2 + 8\,\kappa_{G:G}\,\widehat{G}} - \kappa_{G:G}\left(1 + \frac{C}{\kappa_{C:G}}\right)\right), \\ C &= \frac{1}{4}\left(\sqrt{\left(\kappa_{C:C}\left(1 + \frac{G}{\kappa_{C:G}}\right)\right)^2 + 8\,\kappa_{C:C}\,\widehat{C}} - \kappa_{C:C}\left(1 + \frac{G}{\kappa_{C:G}}\right)\right). \end{aligned} \tag{8}$$

Equations (8) define a map from $\mathbb{R}^2_+$ into itself

$$(G_{n+1}, C_{n+1}) := f(G_n, C_n)$$

given by

$$\begin{aligned} G_{n+1} &= \frac{1}{4}\left(\sqrt{\left(\kappa_{G:G}\left(1 + \frac{C_n}{\kappa_{C:G}}\right)\right)^2 + 8\,\kappa_{G:G}\,\widehat{G}} - \kappa_{G:G}\left(1 + \frac{C_n}{\kappa_{C:G}}\right)\right), \\ C_{n+1} &= \frac{1}{4}\left(\sqrt{\left(\kappa_{C:C}\left(1 + \frac{G_n}{\kappa_{C:G}}\right)\right)^2 + 8\,\kappa_{C:C}\,\widehat{C}} - \kappa_{C:C}\left(1 + \frac{G_n}{\kappa_{C:G}}\right)\right). \end{aligned} \tag{9}$$

A fixed point of this map solves (7). By composing the two component maps and computing a straightforward bound on the maximum absolute value of the derivative of the composition we find that if

$$\frac{1}{16}\frac{\kappa_{G:G}\,\kappa_{C:C}}{\kappa_{C:G}^2}\left(1-\frac{\kappa_{G:G}}{\sqrt{\kappa_{G:G}^2+8\,\kappa_{G:G}\,\widehat{G}}}\right)\left(1-\frac{\kappa_{C:C}}{\sqrt{\kappa_{C:C}^2+8\,\kappa_{C:C}\,\widehat{C}}}\right)<1$$

then the map is a contraction and convergence is linear to a unique non-negative fixed point.

This sufficient condition is verified for many parameter combinations, but in practice, convergence occurs under less stringent conditions on the protein dissociation constants. We verify convergence by ensuring a sufficiently small residual in (7). Once the monomer concentrations G and C are approximated sufficiently, the dimer concentrations $G{:}G$, $C{:}C$, and $C{:}G$ may be computed directly using equations (5).

6.3. **Gene Regulation by Transcription Factors.** The function F_n represents the combined effect of the repressor $C{:}C$ and activator N on clock mRNA production. Using the approach of Shea and Ackers [7, 14, 15], F_n is given by the following probability ratio:

$$F_n(C{:}C, N) = \frac{Z_{\mathrm{ON}}}{Z_{\mathrm{ON}} + Z_{\mathrm{OFF}n}},$$

where Z_{ON} and $Z_{\mathrm{OFF}n}$ are sums of terms representing states where RNAP-II complex is bound or unbound, respectively, to the clock-gene's promoter. The positive integer n is the number of *cis* binding sites for the repressor, while the activator is assumed to have only one binding site. The binding of the repressor and RNAP-II complex is assumed to be mutually exclusive, however binding of repressor and activator is *not* assumed to be mutually exclusive. Transcription proceeds only in those states in which RNAP-II complex is bound, which may or may not occur cooperatively with activator.

Consideration of the two possible RNAP-II bound states gives

$$\begin{aligned} Z_{\mathrm{ON}} &= \rho_P\,P\left(1+\omega_N\,(\rho_N\,N)\right) \\ &= \rho\,(1+\omega_N\,(\rho_N\,N)), \end{aligned}$$

where $\rho := \rho_P\,P$. ρ_N is the binding affinity of the activator N induced by adjacent cells' coordinating signal protein S. The assumption that intercellular coupling is weak relative to intracellular negative feedback means that $\rho_N \ll \rho_{C:C}$. $\omega_N > 1$ is the binding cooperativity between the activator N and the RNAP-II complex.

For one repressor binding site, consideration of all the RNAP-II unbound states gives

$$Z_{\mathrm{OFF1}} = 1 + \rho_{C:C}\,C{:}C + \rho_N\,N + \omega_{N\text{-}C:C}\,(\rho_N\,N)\,(\rho_{C:C}\,C{:}C),$$

where $\omega_{N\text{-}C:C}$ represents the cooperativity between simultaneously bound repressor and activator molecules. Consideration of two repressor binding sites gives

$$\begin{aligned}Z_{\mathrm{OFF}2} = 1 &+ \rho_{C:C\text{-}\square}\, C{:}C + \rho_{\square\text{-}C:C}\, C{:}C + \omega_{C:C}\,(\rho_{C:C\text{-}\square}\, C{:}C)\,(\rho_{\square\text{-}C:C}\, C{:}C) + \\ &\rho_N\, N + \omega_{N\text{-}C:C\text{-}\square}\,(\rho_N\, N)\,(\rho_{C:C\text{-}\square}\, C{:}C) + \omega_{N\text{-}\square\text{-}C:C}\,(\rho_N\, N)\,(\rho_{\square\text{-}C:C}\, C{:}C) + \\ &\qquad \omega_{N\text{-}C:C\text{-}C:C}\,(\rho_N\, N)\,(\rho_{C:C\text{-}\square}\, C{:}C)\,(\rho_{\square\text{-}C:C}\, C{:}C)\,,\end{aligned}$$

where $\omega_{N\text{-}C:C\text{-}\square}$, $\omega_{N\text{-}\square\text{-}C:C}$, and $\omega_{N\text{-}C:C\text{-}C:C}$ represent the cooperativity between simultaneously bound repressor and activator molecules, with the first, second, and both repressor site(s) occupied, respectively. The binding affinities for the $C{:}C$ homodimer to the first and second binding site are $\rho_{C:C\text{-}\square}$ and $\rho_{\square\text{-}C:C}$, respectively, with simultaneous binding cooperativity $\omega_{C:C}$.

To the best of our knowledge, no experimental data are available on the binding affinities and cooperativities for the repressor and activator in PSM cells. In response to this lack of knowledge, two straightforward, simplifying assumptions are as follows:

(1) The activator N and the repressor $C{:}C$ bind independently of each other, so that for one binding site
$$\omega_{N\text{-}C:C} = 1,$$
while for two binding sites
$$\omega_{N\text{-}C:C\text{-}\square} = \omega_{N\text{-}\square\text{-}C:C} = 1 \quad \text{and} \quad \omega_{N\text{-}C:C\text{-}C:C} = \omega_{C:C}.$$

(2) For two binding sites, the binding affinity of the repressor at each binding site is the same, i.e.,
$$\rho_{C:C\text{-}\square} = \rho_{\square\text{-}C:C} =: \rho_{C:C}.$$

Applying these assumptions on the parameters for one binding site gives

$$\begin{aligned}Z_{\mathrm{OFF}1} &= 1 + \rho_{C:C}\, C{:}C + \rho_N\, N + (\rho_N\, N)\,(\rho_{C:C}\, C{:}C) \\ &= 1 + \rho_{C:C}\, C{:}C + \rho_N\, N\,(1 + \rho_{C:C}\, C{:}C) \\ &= (1 + \rho_N\, N)\,(1 + \rho_{C:C}\, C{:}C) \\ &= (1 + \rho_N\, N)\,(1 + \Psi_1(C{:}C))\,,\end{aligned}$$

where $\Psi_1(C{:}C) := \rho_{C:C}\, C{:}C$. For two binding sites,

$$\begin{aligned}Z_{\mathrm{OFF}2} &= 1 + 2\,(\rho_{C:C}\, C{:}C) + \omega_{C:C}\,(\rho_{C:C}\, C{:}C)^2 + \\ &\qquad \rho_N\, N + 2\,(\rho_N\, N)\,(\rho_{C:C}\, C{:}C) + \omega_{C:C}\,(\rho_N\, N)\,(\rho_{C:C}\, C{:}C)^2 \\ &= 1 + 2\,(\rho_{C:C}\, C{:}C) + \omega_{C:C}\,(\rho_{C:C}\, C{:}C)^2 + \\ &\qquad \rho_N\, N\left(1 + 2\,(\rho_{C:C}\, C{:}C) + \omega_{C:C}\,(\rho_{C:C}\, C{:}C)^2\right) \\ &= (1 + \rho_N\, N)\left(1 + 2\,(\rho_{C:C}\, C{:}C) + \omega_{C:C}\,(\rho_{C:C}\, C{:}C)^2\right) \\ &= (1 + \rho_N\, N)\,(1 + \Psi_2(C{:}C))\,,\end{aligned}$$

where $\Psi_2(C{:}C) := 2\,\rho_{C:C}\,C{:}C + \omega_{C:C}\,(\rho_{C:C}\,C{:}C)^2$. Altogether, for $n = 1$ or 2, the function F_n is given by

$$F_n(C{:}C, N) := \frac{\rho\,(1 + \omega_N\,\rho_N\,N)}{\rho\,(1 + \omega_N\,\rho_N\,N) + (1 + \rho_N\,N)\,(1 + \Psi_n(C{:}C))}. \tag{10}$$

References

1. Lewis J (2003) Autoinhibition with transcriptional delay: a simple mechanism for the zebrafish somitogenesis oscillator. Curr Biol 13: 1398-408.
2. Holley SA (2007) The genetics and embryology of zebrafish metamerism. Dev Dyn 236: 1422-49.
3. Kanki JP, Ho RK (1997) The development of the posterior body in zebrafish. Development 124: 881-93.
4. Schroter C, Herrgen L, Cardona A, Brouhard GJ, Feldman B, et al. (2008) Dynamics of zebrafish somitogenesis. Dev Dyn 237: 545-53.
5. Giudicelli F, Özbudak EM, Wright GJ, Lewis J (2007) Setting the tempo in development: an investigation of the zebrafish somite clock mechanism. PLoS Biol 5: e150.
6. Özbudak EM, Lewis J (2008) Notch signalling synchronizes the zebrafish segmentation clock but is not needed to create somite boundaries. PLoS Genet 4: e15.
7. Buchler NE, Gerland U, Hwa T (2003) On schemes of combinatorial transcription logic. Proc Natl Acad Sci U S A 100: 5136-41.
8. Mara A, Schroeder J, Chalouni C, Holley SA (2007) Priming, initiation and synchronization of the segmentation clock by deltaD and deltaC. Nat Cell Biol 9: 523-30.
9. Sieger D, Tautz D, Gajewski M (2003) The role of Suppressor of Hairless in Notch mediated signalling during zebrafish somitogenesis. Mech Dev 120: 1083-94.
10. Hirata H, Bessho Y, Kokubu H, Masamizu Y, Yamada S, et al. (2004) Instability of Hes7 protein is crucial for the somite segmentation clock. Nat Genet 36: 750-4.
11. Buchler NE, Gerland U, Hwa T (2005) Nonlinear protein degradation and the function of genetic circuits. Proc Natl Acad Sci U S A 102: 9559-64.
12. Zeiser S, Liebscher HV, Tiedemann H, Rubio-Aliaga I, Przemeck GKH, et al. (2006) Number of active transcription factor binding sites is essential for the Hes7 oscillator. Theor Biol Med Model 3: 11.
13. Baker RE, Maini PK (2007) Travelling gradients in interacting morphogen systems. Math Biosci 209: 30-50.
14. Santillán M, Mackey MC (2004) Why the lysogenic state of phage lambda is so stable: a mathematical modeling approach. Biophys J 86: 75-84.
15. Shea MA, Ackers GK (1985) The OR control system of bacteriophage lambda. A physical-chemical model for gene regulation. J Mol Biol 181: 211-30.
