# Supplementary material for: Somitogenesis Clock-Wave Initiation Requires Differential Decay and Multiple Binding Sites for Clock Protein
Source: PLoS Comput Biol. 2010 Apr 1;6(4):e1000728. doi: 10.1371/journal.pcbi.1000728 (PMC2848544; doi:10.1371/journal.pcbi.1000728)
Supplement: Text S2 — Computer Matlab code used in simulations. The code is available upon request from the authors. (0.50 MB PDF) [file pcbi.1000728.s002.pdf]

SUPPORTING TEXT 7 – MATLAB CODES

SOMITOGENESIS CLOCK-WAVE INITIATION REQUIRES DIFFERENTIAL DECAY AND MULTIPLE BINDING SITES FOR CLOCK PROTEIN.

MARK CAMPANELLI AND TOMÁŠ GEDEON

1. Matlab Code Listings

1.1. Simulation Related Code.

1.1.1. `somitogenesis.m`. This is the main simulation function for the mathematical model. This code calls the built-in Matlab DDE solver `ddesd` by default, but it also works with `dde23`.

```
function sol= somitogenesis(params)

% Usage:
%  sol= somitogenesis(params)
%
% Simulates zebrafish somitogenesis in posterior PSM.
%  Single clock-gene is HER7, denoted H7 (protein) or h7 (mRNA).
%  Control protein is Her13.2, denoted H13.
%  Coordinating-signal gene (optional) is DELTAC, denoted
%   D (protein) or d (mRNA).
%
% Note: To get a default params object for the input argument use
%  params= Params()

%somitogenesisTic= tic;
 % Select dde solver
  ddeFunH= @ddesd; % @dde23 OR @ddesd
 % Select rhs system to solve (see nested functions below)
  if params.numGenes == 1 % HER7 only
    if params.numBindingSites == 1 % Repressor binding sites
      rhsSystem= @rhs1Gene1Site;
    elseif params.numBindingSites == 2
      rhsSystem= @rhs1Gene2Site;
    end
  elseif params.numGenes == 2 % HER7 and DELTAC
    if params.numBindingSites == 1 % Repressor binding sites
      rhsSystem= @rhs2Gene1Site;
    elseif params.numBindingSites == 2
```

Date: October, 2009.

```
      rhsSystem= @rhs2Gene2Site;
    end
  end
 % Optimizations to avoid inefficient params object access
  numEquations= params.numEquations;
  birthTimes= params.birthTimes;
  H7TotalIndices= params.H7TotalIndices;
  h7Indices= params.h7Indices;
  DIndices= params.DIndices;
  dIndices= params.dIndices;
  H7TotalDelayedh7Indices= params.H7TotalDelayedh7Indices;
  DDelayedh7Indices= params.DDelayedh7Indices;
  h7DelayedH7Indices= params.h7DelayedH7Indices;
  H7TotalDelayeddIndices= params.H7TotalDelayeddIndices;
  dDelayedDIndices= params.dDelayedDIndices;
  rho= params.rho;
  kH13H13= params.kH13H13;
  kH7H7= params.kH7H7;
  kH7H13= params.kH7H13;
  tau_h7= params.tau_h7;
%  tau_H7= params.tau_H7;
  aH7= params.aH7;
  bH7= params.bH7;
  bH7H7= params.bH7H7;
  bH7H13= params.bH7H13;
  gH7= params.gH7;
  rH7H7= params.rH7H7;
  wH7H7_H7H7= params.wH7H7_H7H7;
  dH7= params.dH7;
  tau_d= params.tau_d;
%  tau_D= params.tau_D;
  aD= params.aD;
  bD= params.bD;
  gD= params.gD;
  rN= params.rN;
  wN= params.wN;
  wN_H7H7= params.wN_H7H7;
  wN_H7H7_H7H7= params.wN_H7H7_H7H7;
  dD= params.dD;
  couplingMatrix= params.couplingMatrix;
  gradientFuncH= str2func(params.gradientFunc);
  H13TotalMax= params.H13TotalMax;
  H13TotalMin= params.H13TotalMin;
  H13TotalHalfLife= params.H13TotalHalfLife;

  if isstruct(params.history) % Continue existing solution
```

```
  % Assumes longer params.timeInterval for continuation
  % TBD: How to track/record continuations?
    sol= ddeFunH(rhsSystem, params.timeLags, params.history, [params.history.params....
timeInterval(2), params.timeInterval(2)], params.ddeOptions);
    if ~isempty(sol.xe) % Solution went negative
      warning('Zero-crossing in solution during continuation.');
    end
    sol.params= params;
    sol.statistics= sol.params.history.statistics;
  else % Start a new solution
    if params.settlingTime > 0
     % Compute "settling time" portion of solution
      sol= ddeFunH(rhsSystem, params.timeLags, params.history, [params.timeInterval(1) - ...
params.settlingTime, params.timeInterval(1)], params.ddeOptions);
      if isempty(sol.xe) % Solution did not go negative
        sol.params= params; % Needed for calcPerMedAmp() call
       % Calculate periods, medians, and amplitudes at end of settlingTime
        [statistics.periods, statistics.medians, statistics.amplitudes]= calcPerMedAmp(...
sol);
       % Update default tailbud period
        if ~any(isnan(statistics.periods)) && ~any(statistics.periods <= 0)
          periodMean= mean(statistics.periods);
          if all(abs((statistics.periods - periodMean)/periodMean) < params.periodTol)
            params.tailbudPeriod= periodMean;
            birthTimes= params.birthTimes; % Update local copy
          end
        end
       % Continue with "time of interest" portion of solution
        if params.timeInterval(2) - params.timeInterval(1) > 0
          sol= ddeFunH(rhsSystem, params.timeLags, sol, params.timeInterval, params....
ddeOptions);
          if ~isempty(sol.xe) % Solution went negative
            warning('Zero-crossing in solution during "time of interest".');
          end
        end
      else
        warning('Zero-crossing in solution during "settling time".');
      end
    elseif params.timeInterval(2) - params.timeInterval(1) > 0
     % Compute solution without any "settling time"
      statistics.periods= nan(numEquations, 1);
      statistics.medians= nan(numEquations, 1);
      statistics.amplitudes= nan(numEquations, 1);
      sol= ddeFunH(rhsSystem, params.timeLags, params.history, params.timeInterval, ...
params.ddeOptions);
      if ~isempty(sol.xe) % Solution went negative
```

```
        warning('Zero-crossing␣in␣solution␣during␣"time␣of␣interest".');
      end
    else
      warning('No␣non-empty␣time-interval␣on␣which␣to␣solve␣the␣system.');
      sol.params= params; % Record parameters object in solution structure
      return;
    end
    sol.params= params; % Record parameters object in solution structure
    sol.statistics= statistics; % Record statistics in solution structure
  end
%toc(somitogenesisTic)
  if ~params.fastModeBool
   % Calculate Her13.2 & Her7 monomer & dimer concentrations
    sol.output.H13Total= gradientFuncH(repmat(sol.x, length(birthTimes), 1) - repmat(...
birthTimes, 1, length(sol.x)), H13TotalMax, H13TotalMin, H13TotalHalfLife);
    [sol.output.H13, sol.output.H7, sol.output.H13H13, sol.output.H7H7, sol.output.H7H13...
]= calcDimerization(sol.output.H13Total, sol.y(H7TotalIndices,:), kH13H13, kH7H7, kH7H13)...
;
    negativityCheck= (sol.output.H7 < 0) | (sol.output.H13 < 0) | (sol.output.H7H7 < 0) |...
 (sol.output.H13H13 < 0) | (sol.output.H7H13 < 0);
    if any(negativityCheck(:))
      warning('Negative␣copy␣number␣(possibly␣near␣zero)␣detected␣in␣calcDimerization()␣...
computation.');
    end
    plotSolution(sol); % Plot results
  end

 % Nested functions
  function dzdt= rhs1Gene1Site(t, z, Z)
    dzdt= zeros(numEquations, 1); % Preallocate
   % Calculate fast dimerization (Her monomer and dimer amounts)
    [H13, H7, H13H13, H7H7, H7H13]= calcDimerization(gradientFuncH(t - birthTimes, ...
H13TotalMax, H13TotalMin, H13TotalHalfLife), z(H7TotalIndices), kH13H13, kH7H7, kH7H13);
    [H13Delayedh7, H7Delayedh7, H13H13Delayedh7, H7H7Delayedh7, H7H13Delayedh7]= ...
calcDimerization(gradientFuncH(t - birthTimes - tau_h7, H13TotalMax, H13TotalMin, ...
H13TotalHalfLife), Z(H7TotalDelayedh7Indices), kH13H13, kH7H7, kH7H13);
  % System of differential equations
   % Her7 total protein
    dzdt(H7TotalIndices)= aH7.*Z(h7DelayedH7Indices) - bH7.*H7 - 2*bH7H7.*H7H7 - bH7H13.*...
H7H13;
   % Her7 mRNA
    dzdt(h7Indices)= gH7.*rho./(rho + 1 + rH7H7.*H7H7Delayedh7) - dH7.*z(h7Indices);
  end

  function dzdt= rhs1Gene2Site(t, z, Z)
    dzdt= zeros(numEquations, 1); % Preallocate
```

```
    % Calculate fast dimerization (Her monomer and dimer amounts)
      [H13, H7, H13H13, H7H7, H7H13]= calcDimerization(gradientFuncH(t - birthTimes, ...
H13TotalMax, H13TotalMin, H13TotalHalfLife), z(H7TotalIndices), kH13H13, kH7H7, kH7H13);
      [H13Delayedh7, H7Delayedh7, H13H13Delayedh7, H7H7Delayedh7, H7H13Delayedh7]= ...
calcDimerization(gradientFuncH(t - birthTimes - tau_h7, H13TotalMax, H13TotalMin, ...
H13TotalHalfLife), Z(H7TotalDelayedh7Indices), kH13H13, kH7H7, kH7H13);
  % System of differential equations
    % Her7 total protein
      dzdt(H7TotalIndices)= aH7.*Z(h7DelayedH7Indices) - bH7.*H7 - 2*bH7H7.*H7H7 - bH7H13.*...
H7H13;
    % Her7 mRNA
      dzdt(h7Indices)= gH7.*rho./(rho + 1 + 2*rH7H7.*H7H7Delayedh7 + wH7H7_H7H7.*(rH7H7.*...
H7H7Delayedh7).^2) - dH7.*z(h7Indices);
  end

  function dzdt= rhs2Gene1Site(t, z, Z)
      dzdt= zeros(numEquations, 1); % Preallocate
    % Calculate fast dimerization (Her monomer and dimer amounts)
      [H13, H7, H13H13, H7H7, H7H13]= calcDimerization(gradientFuncH(t - birthTimes, ...
H13TotalMax, H13TotalMin, H13TotalHalfLife), z(H7TotalIndices), kH13H13, kH7H7, kH7H13);
      [H13Delayedh7, H7Delayedh7, H13H13Delayedh7, H7H7Delayedh7, H7H13Delayedh7]= ...
calcDimerization(gradientFuncH(t - birthTimes - tau_h7, H13TotalMax, H13TotalMin, ...
H13TotalHalfLife), Z(H7TotalDelayedh7Indices), kH13H13, kH7H7, kH7H13);
      [H13Delayedd, H7Delayedd, H13H13Delayedd, H7H7Delayedd, H7H13Delayedd]= ...
calcDimerization(gradientFuncH(t - birthTimes - tau_d, H13TotalMax, H13TotalMin, ...
H13TotalHalfLife), Z(H7TotalDelayeddIndices), kH13H13, kH7H7, kH7H13);
    % Calculate NICD from coupling
      NDelayedh7= couplingMatrix*Z(DDelayedh7Indices);
  % System of differential equations
    % Her7 total protein
      dzdt(H7TotalIndices)= aH7.*Z(h7DelayedH7Indices) - bH7.*H7 - 2*bH7H7.*H7H7 - bH7H13.*...
H7H13;
    % Her7 mRNA
      dzdt(h7Indices)= gH7.*rho.*(1 + wN.*rN.*NDelayedh7)./(rho.*(1 + wN.*rN.*NDelayedh7) +...
 (1 + rH7H7.*H7H7Delayedh7) + rN.*NDelayedh7.*(1 + wN_H7H7.*rH7H7.*H7H7Delayedh7)) - dH7...
.*z(h7Indices);
    % Delta protein
      dzdt(DIndices)= aD.*Z(dDelayedDIndices) - bD.*z(DIndices);
    % Delta mRNA
      dzdt(dIndices)= gD.*rho./(rho + 1 + rH7H7.*H7H7Delayedd) - dD.*z(dIndices);
  end

  function dzdt= rhs2Gene2Site(t, z, Z)
      dzdt= zeros(numEquations, 1); % Preallocate
    % Calculate fast dimerization (Her monomer and dimer amounts)
```

```
    [H13, H7, H13H13, H7H7, H7H13]= calcDimerization(gradientFuncH(t - birthTimes, ...
H13TotalMax, H13TotalMin, H13TotalHalfLife), z(H7TotalIndices), kH13H13, kH7H7, kH7H13);
    [H13Delayedh7, H7Delayedh7, H13H13Delayedh7, H7H7Delayedh7, H7H13Delayedh7]= ...
calcDimerization(gradientFuncH(t - birthTimes - tau_h7, H13TotalMax, H13TotalMin, ...
H13TotalHalfLife), Z(H7TotalDelayedh7Indices), kH13H13, kH7H7, kH7H13);
    [H13Delayedd, H7Delayedd, H13H13Delayedd, H7H7Delayedd, H7H13Delayedd]= ...
calcDimerization(gradientFuncH(t - birthTimes - tau_d, H13TotalMax, H13TotalMin, ...
H13TotalHalfLife), Z(H7TotalDelayeddIndices), kH13H13, kH7H7, kH7H13);
   % Calculate NICD from coupling
    NDelayedh7= couplingMatrix*Z(DDelayedh7Indices);
  % System of differential equations
   % Her7 total protein
    dzdt(H7TotalIndices)= aH7.*Z(h7DelayedH7Indices) - bH7.*H7 - 2*bH7H7.*H7H7 - bH7H13.*...
H7H13;
   % Her7 mRNA
    dzdt(h7Indices)= gH7.*rho.*(1 + wN.*rN.*NDelayedh7)./(rho.*(1 + wN.*rN.*NDelayedh7) +...
 (1 + 2*rH7H7.*H7H7Delayedh7 + wH7H7_H7H7.*(rH7H7.*H7H7Delayedh7).^2) + rN.*NDelayedh7...
.*(1 + 2*wN_H7H7.*rH7H7.*H7H7Delayedh7 + wN_H7H7_H7H7.*wH7H7_H7H7.*(rH7H7.*H7H7Delayedh7)...
.^2)) - dH7.*z(h7Indices);
   % Delta protein
    dzdt(DIndices)= aD.*Z(dDelayedDIndices) - bD.*z(DIndices);
   % Delta mRNA
    dzdt(dIndices)= gD.*rho./(rho + 1 + rH7H7.*H7H7Delayedd) - dD.*z(dIndices);
  end
end
```

1.1.2. `Params.m`. This is the class definition for the parameters object that holds the system parameters and other configuration information for model simulation. Objects of this class can provide either deterministic parameter values or random values drawn from a specified normal or uniform distribution. The code is lengthy and mostly uninteresting, but it ensures accurate and repeatable parameter specification.

```
classdef Params

 % Class definition for managing parameters and settings for the
 %  somitogenesis.m model.
 % The parameter mean is the nominal value of the parameter. If
 %  noisy parameters are turned off, then the nominal (scalar)
 %  value is returned, otherwise the parameters returned are
 %  random vectors drawn from a specified distribution, one
 %  parameter for each cell. The same default noise seed is used
 %  unless a different seed is selected. This allows repeatable
 %  sampling.

  properties (Constant= true)
    mRNAProductionRate= 33;
    periodTol= 1e-2;
  end
```

```
properties % Defaults only used for simpler Constructor logic
% Simulation/Solver parameters
  noiseBool= false; noiseType; noiseSeed;
  noiseProportionOfMean; timeInterval; settlingTime; history;
  ddeOptions; fastModeBool;
% Model parameters
 % Cell/PSM parameters
  cellsPerSomite; somitesInPSM; tailbudPeriod;
  cellArraySize= [2 1]; numGenes= 2;
 % Cell background parameters
  rhoMean; kH13H13Mean; kH7H7Mean; kH7H13Mean;
 % HER7 Parameters
  numBindingSites; tau_h7Mean= 5.659226564114478; tau_H7Mean= 1.7; aH7Mean;
  bH7Mean; bH7H7Mean; bH7H13Mean; dH7Mean; rH7H7Mean;
  wH7H7_H7H7Mean;
 % DELTAC/NOTCH Parameters
  couplingBool; tau_dMean= 12.4; tau_DMean= 20; aDMean; bDMean;
  dDMean; rNMean; wNMean; wN_H7H7Mean; wN_H7H7_H7H7Mean;
 % Her13.2/gradient parameters
  gradientFunc; H13TotalMaxMean; H13TotalMinMean= 0;
  H13TotalHalfLifeMean;
end

properties (Dependent= true, SetAccess= private)
  timeLags; somiteGrowthRate; cellBirthRate; birthTimes;
  numCells; equationsPerCell; numEquations; gH7Mean; gDMean;
  couplingMatrix;
 % Indexing vectors
  H7TotalIndices; h7Indices; DIndices; dIndices;
  H7TotalDelayedh7Indices; h7DelayedH7Indices;
  DDelayedh7Indices; H7TotalDelayeddIndices; dDelayedDIndices;
 % Computed parameters
  rho; kH13H13; kH7H7; kH7H13; tau_h7; tau_H7; aH7; bH7; bH7H7;
  bH7H13; gH7; rH7H7; wH7H7_H7H7; dH7; tau_d; tau_D; aD; bD;
  gD; rN; wN; wN_H7H7; wN_H7H7_H7H7; dD; H13TotalMax;
  H13TotalMin; H13TotalHalfLife;
end

properties (SetAccess = private)
 % Random number streams for reproducibility purposes
  rhoStream; kH13H13Stream; kH7H7Stream; kH7H13Stream;
  tau_h7Stream; tau_H7Stream; aH7Stream; bH7Stream;
  bH7H7Stream; bH7H13Stream; gH7Stream; rH7H7Stream;
  wH7H7_H7H7Stream; dH7Stream; tau_dStream; tau_DStream;
  aDStream; bDStream; gDStream; rNStream; wNStream;
```

```
    wN_H7H7Stream; wN_H7H7_H7H7Stream; dDStream;
    H13TotalMaxStream; H13TotalMinStream; H13TotalHalfLifeStream;
  end

  methods % CONSTRUCTOR
    function params= Params(noiseBool)
      if (nargin > 0)
        params.noiseBool= noiseBool;
      else
        params.noiseBool= params.noiseBool;
      end
      params.noiseType= 'normal';
      params.noiseSeed= 0;
      params.noiseProportionOfMean= 0.01;
      params= initStreams(params);
      params.timeInterval= [0 500];
      params.settlingTime= 250;
      params.history= 0; % Becomes constant zero vector
      params.ddeOptions= ddeset('AbsTol',1e-15, 'RelTol',1e-4, 'MaxStep', 5, 'Events',...
@zeroCrossingEvent);
      params.fastModeBool= false;
      params.cellsPerSomite= 5;
      params.somitesInPSM= 7;
      params.tailbudPeriod= 30; % May be updated after settlingTime to a computed period
      params.cellArraySize= params.cellArraySize; % [ax lat]
      params.numGenes= params.numGenes; % 1 or 2 genes, HER7 w/possibly DELTAC
      params.rhoMean= 0.639080069045828; % rRNAP*RNAP, unitless
      params.kH13H13Mean= 22.497936196496877; % copies
      params.kH7H7Mean= 155.1730668730243; % copies
      params.kH7H13Mean= 583.9263395489304; % copies
      params.numBindingSites= 2; % 1 or 2 for Her7 dimer
      params.tau_h7Mean= params.tau_h7Mean; % min
      params.tau_H7Mean= params.tau_H7Mean; % min
      params.aH7Mean= 4.5; % Her7 protein copies/min
      params.bH7Mean= 0.497249043509659; % min^(-1)
      params.bH7H7Mean= 0; % min^(-1)
      params.bH7H13Mean= 0; % min^(-1)
      params.rH7H7Mean= 0.944246962644303; % copies^(-1)
      params.wH7H7_H7H7Mean= 30.859932569137623; % unitless
      params.dH7Mean= 1/(0.6*8.1); % min^(-1)
      params.couplingBool= true;
      params.tau_dMean= params.tau_dMean; % min
      params.tau_DMean= params.tau_DMean; % min
      params.aDMean= 4.5; % DeltaC protein copies/min
      params.bDMean= 0.23; % min^(-1)
      params.rNMean= params.rH7H7Mean/10; % copies^(-1)
```

```
      params.wNMean= 25; % unitless
      params.wN_H7H7Mean= 1; % unitless
      params.wN_H7H7_H7H7Mean= 1; % unitless
      params.dDMean= 1/(0.6*6.1); % min^(-1)
      params.gradientFunc= 'sigmoidalGradient';
      params.H13TotalMaxMean= 700; % Copies (in nucleus)
      params.H13TotalMinMean= params.H13TotalMinMean; % Copies (in nucleus)
      params.H13TotalHalfLifeMean= 30; % min
    end % Params Constructor
  end % CONSTRUCTOR

  methods % Public SET methods
    function params= set.noiseBool(params, noiseBool)
      if ~islogical(noiseBool)
        disp('noiseBool␣not␣set,␣true/false␣noiseBool␣required.')
      else
        params.noiseBool= noiseBool;
      end
    end % noiseBool set function
    function params= set.noiseType(params, noiseType)
      if ~ischar(noiseType) || ~(strcmpi(noiseType, 'uniform') || strcmpi(noiseType, '...
normal'))
        disp('noiseType␣not␣set,␣uniform␣OR␣normal␣noiseType␣required.')
      else
        params.noiseType= noiseType;
      end
    end % noiseType set function
    function params= set.noiseSeed(params, noiseSeed)
      if ~isscalar(noiseSeed) || (noiseSeed <  0)
        disp('noiseSeed␣not␣set,␣0␣<=␣noiseSeed␣required.')
      else
        params.noiseSeed= noiseSeed;
        params= initStreams(params);
      end
    end % noiseSeed set function
    function params= set.noiseProportionOfMean(params, noiseProportionOfMean)
      if ~isscalar(noiseProportionOfMean) || (noiseProportionOfMean <  0)
        disp('noiseProportionOfMean␣not␣set,␣0␣<=␣noiseProportionOfMean␣required.')
      else
        params.noiseProportionOfMean= noiseProportionOfMean;
      end
    end % noiseProportionOfMean set function
    function params= set.timeInterval(params, timeInterval)
     if ~isvector(timeInterval) || any(size(timeInterval) ~= [1 2]) || (timeInterval(1) ...
> timeInterval(2))
       disp('timeInterval␣not␣set,␣timeInterval(1)␣<=␣timeInterval(2)␣required.');
```

```
      else
        params.timeInterval(1,1)= timeInterval(1);
        params.timeInterval(1,2)= timeInterval(2);
      end
    end % timeInterval set function
    function params= set.settlingTime(params, settlingTime)
      if ~isscalar(settlingTime) || (settlingTime < 0)
        disp('settlingTime␣not␣set,␣0␣<=␣settlingTime␣required.');
      else
        params.settlingTime= settlingTime;
      end
    end % settlingTime set function
    function params= set.history(params, history)
      if isstruct(history)
        if isfield(history, 'solver') && (strcmp(history.solver, 'dde23') || strcmp(...
history.solver, 'ddesd'))
          params.history= history;
        else
          disp('history␣not␣set,␣unrecognized␣previous␣solution.');
        end
      elseif isa(history, 'function_handle')
        params.history= history;
      elseif isscalar(history)
        if history < 0
          disp('history␣not␣set,␣0␣<=␣history␣required.');
        else
          params.history= history*ones(1, params.numEquations);
        end
      elseif isvector(history)
        if length(history) ~= params.numEquations
          disp('history␣not␣set,␣length(history)␣==␣numEquations␣required.');
        else
          params.history= history;
        end
      else
        disp('history␣not␣set,␣unrecognized␣history␣type.');
      end
    end % history set function
    function params= set.ddeOptions(params, ddeOptions)
      if ~isstruct(ddeOptions)
        disp('ddeOptions␣not␣set,␣use␣ddeset()␣to␣get␣the␣appropriate␣struct.');
      else
        params.ddeOptions= ddeOptions;
      end
    end % ddeOptions set function
    function params= set.fastModeBool(params, fastModeBool)
```

```
      if ~islogical(fastModeBool)
        disp('fastModeBool␣not␣set,␣true/false␣fastModeBool␣required.');
      else
        params.fastModeBool= fastModeBool;
      end
    end % fastModeBool set function
    function params= set.cellsPerSomite(params, cellsPerSomite)
      if ~isscalar(cellsPerSomite) || (cellsPerSomite <= 0)
        disp('cellsPerSomite␣not␣set,␣0␣<␣cellsPerSomite␣required.');
      else
        params.cellsPerSomite= cellsPerSomite;
      end
    end % cellsPerSomite set function
    function params= set.somitesInPSM(params, somitesInPSM)
      if ~isscalar(somitesInPSM) || (somitesInPSM <= 0)
        disp('somitesInPSM␣not␣set,␣0␣<␣somitesInPSM␣required.');
      else
        params.somitesInPSM= somitesInPSM;
      end
    end % somitesInPSM set function
    function params= set.tailbudPeriod(params, tailbudPeriod)
      if ~isscalar(tailbudPeriod) || (tailbudPeriod <= 0)
        disp('tailbudPeriod␣not␣set,␣0␣<␣tailbudPeriod␣required.');
      else
        params.tailbudPeriod= tailbudPeriod;
      end
    end % tailbudPeriod set function
    function params= set.cellArraySize(params, cellArraySize)
      if ~isvector(cellArraySize) || any(size(cellArraySize) ~= [1 2]) || (rem(...
cellArraySize(1),1) ~= 0) || (cellArraySize(1) < 0) || (rem(cellArraySize(2),1) ~= 0) || ...
(cellArraySize(2) < 0)
        disp('cellArraySize␣not␣set,␣cellArraySize=␣[M␣N],␣M␣and␣N␣positive␣integers,␣...
required.');
      elseif any(params.cellArraySize ~= cellArraySize)
        if params.numCells > 1 % Existing value
          params.cellArraySize= cellArraySize;
          if params.numCells == 1 % New value
            params.couplingBool= false;
          else % params.numCells > 1
            params.couplingBool= params.couplingBool;
          end
        else % Existing params.numCells == 1
          params.cellArraySize= cellArraySize;
          if params.numCells > 1
            params.couplingBool= true;
          end
```

```
      end
      if isvector(params.history)
        disp('Notice: history reset to default constant zero vector.')
        params.history= 0;
      else
       disp('Notice: Existing history may be invalid.');
      end
    end
  end % cellArraySize set function
  function params= set.numGenes(params, numGenes)
    if ~isscalar(numGenes) || ((numGenes ~= 1) && (numGenes ~= 2))
      disp('numGenes not set, numGenes= 1 or 2 required.');
    elseif params.numGenes ~= numGenes
      params.numGenes= numGenes;
      if isvector(params.history)
        disp('Notice: history reset to default constant zero vector.')
        params.history= 0;
      else
        disp('Notice: Existing history may be invalid.')
      end
      if params.numGenes == 1
        params.couplingBool= false; % Turn off coupling
      elseif params.numGenes == 2
        if params.numCells > 1
          if ~params.couplingBool
            disp('Notice: couplingBool reset to default true.');
            params.couplingBool= true; % Coupling on by default
          end
        else % params.numCells == 1
          params.couplingBool= false;
        end
      end
    end
  end % numGenes set function
  function params= set.rhoMean(params, rhoMean)
    if ~isscalar(rhoMean) || (rhoMean < 0)
      disp('rhoMean not set, 0 <= rhoMean required.');
    else
      params.rhoMean= rhoMean;
    end
  end % rhoMean set function
  function params= set.kH13H13Mean(params, kH13H13Mean)
    if ~isscalar(kH13H13Mean) || (kH13H13Mean <= 0)
      disp('kH13H13Mean not set, 0 < kH13H13Mean required.');
    else
      params.kH13H13Mean= kH13H13Mean;
```

```
  end
end % kH13H13Mean set function
function params= set.kH7H7Mean(params, kH7H7Mean)
  if ~isscalar(kH7H7Mean) || (kH7H7Mean <= 0)
    disp('kH7H7Mean not set, 0 < kH7H7Mean required.');
  else
    params.kH7H7Mean= kH7H7Mean;
  end
end % kH7H7Mean set function
function params= set.kH7H13Mean(params, kH7H13Mean)
  if ~isscalar(kH7H13Mean) || (kH7H13Mean <= 0)
    disp('kH7H13Mean not set, 0 < kH7H13Mean required.');
  else
    params.kH7H13Mean= kH7H13Mean;
  end
end % kH7H13Mean set function
function params= set.numBindingSites(params, numBindingSites)
  if ~isscalar(numBindingSites) || ((numBindingSites ~= 1) && (numBindingSites ~= 2))
    disp('numBindingSites not set, numBindingSites= 1 or 2 required.');
  else
    params.numBindingSites= numBindingSites;
  end
end % numBindingSites set function
function params= set.tau_h7Mean(params, tau_h7Mean)
  if ~isscalar(tau_h7Mean) || (tau_h7Mean <= 0)
    disp('tau_h7Mean not set, 0 < tau_h7Mean required.');
  else
    params.tau_h7Mean= tau_h7Mean;
  end
end % tau_h7Mean set function
function params= set.tau_H7Mean(params, tau_H7Mean)
  if ~isscalar(tau_H7Mean) || (tau_H7Mean <= 0)
    disp('tau_H7Mean not set, 0 < tau_H7Mean required.');
  else
    params.tau_H7Mean= tau_H7Mean;
  end
end % tau_H7Mean set function
function params= set.aH7Mean(params, aH7Mean)
  if ~isscalar(aH7Mean) || (aH7Mean < 0)
    disp('aH7Mean not set, 0 <= aH7Mean required.');
  else
    params.aH7Mean= aH7Mean;
  end
end % aH7Mean set function
function params= set.bH7Mean(params, bH7Mean)
  if ~isscalar(bH7Mean) || (bH7Mean < 0)
```

```
      disp('bH7Mean␣not␣set,␣0␣<=␣bH7Mean␣required.');
    else
      params.bH7Mean= bH7Mean;
    end
  end % bH7Mean set function
  function params= set.bH7H7Mean(params, bH7H7Mean)
    if ~isscalar(bH7H7Mean) || (bH7H7Mean < 0)
      disp('bH7H7Mean␣not␣set,␣0␣<=␣bH7H7Mean␣required.');
    else
      params.bH7H7Mean= bH7H7Mean;
    end
  end % bH7H7Mean set function
  function params= set.bH7H13Mean(params, bH7H13Mean)
    if ~isscalar(bH7H13Mean) || (bH7H13Mean < 0)
      disp('bH7H13Mean␣not␣set,␣0␣<=␣bH7H13Mean␣required.');
    else
      params.bH7H13Mean= bH7H13Mean;
    end
  end % bH7H13Mean set function
  function params= set.rH7H7Mean(params, rH7H7Mean)
    if ~isscalar(rH7H7Mean) || (rH7H7Mean < 0)
      disp('rH7H7Mean␣not␣set,␣0␣<=␣rH7H7Mean␣required.');
    else
      params.rH7H7Mean= rH7H7Mean;
    end
  end % rH7H7Mean set function
  function params= set.wH7H7_H7H7Mean(params, wH7H7_H7H7Mean)
    if ~isscalar(wH7H7_H7H7Mean) || (wH7H7_H7H7Mean < 0)
      disp('wH7H7_H7H7Mean␣not␣set,␣0␣<=␣wH7H7_H7H7Mean␣required.');
    else
      params.wH7H7_H7H7Mean= wH7H7_H7H7Mean;
    end
  end % wH7H7_H7H7Mean set function
  function params= set.dH7Mean(params, dH7Mean)
    if ~isscalar(dH7Mean) || (dH7Mean < 0)
      disp('dH7Mean␣not␣set,␣0␣<=␣dH7Mean␣required.');
    else
      params.dH7Mean= dH7Mean;
    end
  end % dH7Mean set function
  function params= set.couplingBool(params, couplingBool)
    if ~islogical(couplingBool)
      disp('couplingBool␣not␣set,␣true/false␣couplingBool␣required.');
    elseif couplingBool && ((params.numGenes ~= 2) || (params.numCells == 1))
      disp('couplingBool␣not␣set,␣numGenes␣==␣2␣and␣numCells␣>=␣2␣required.');
    else
```

```
      params.couplingBool= couplingBool;
    end
  end % couplingBool set function
  function params= set.tau_dMean(params, tau_dMean)
    if ~isscalar(tau_dMean) || (tau_dMean <= 0)
      disp('tau_dMean not set, 0 < tau_dMean required.');
    else
      params.tau_dMean= tau_dMean;
    end
  end % tau_dMean set function
  function params= set.tau_DMean(params, tau_DMean)
    if ~isscalar(tau_DMean) || (tau_DMean <= 0)
      disp('tau_DMean not set, 0 < tau_DMean required.');
    else
      params.tau_DMean= tau_DMean;
    end
  end % tau_DMean set function
  function params= set.aDMean(params, aDMean)
    if ~isscalar(aDMean) || (aDMean < 0)
      disp('aDMean not set, 0 <= aDMean required.');
    else
      params.aDMean= aDMean;
    end
  end % aDMean set function
  function params= set.bDMean(params, bDMean)
    if ~isscalar(bDMean) || (bDMean < 0)
      disp('bDMean not set, 0 <= bDMean required.');
    else
      params.bDMean= bDMean;
    end
  end % bDMean set function
  function params= set.rNMean(params, rNMean)
    if ~isscalar(rNMean) || (rNMean < 0)
      disp('rNMean not set, 0 <= rNMean required.');
    else
      params.rNMean= rNMean;
    end
  end % rNMean set function
  function params= set.wNMean(params, wNMean)
    if ~isscalar(wNMean) || (wNMean < 0)
      disp('wNMean not set, 0 <= wNMean required.');
    else
      params.wNMean= wNMean;
    end
  end % wNMean set function
  function params= set.wN_H7H7Mean(params, wN_H7H7Mean)
```

```
      if ~isscalar(wN_H7H7Mean) || (wN_H7H7Mean < 0)
        disp('wN_H7H7Mean␣not␣set,␣0␣<=␣wN_H7H7Mean␣required.');
      else
        params.wN_H7H7Mean= wN_H7H7Mean;
      end
    end % wN_H7H7Mean set function
    function params= set.wN_H7H7_H7H7Mean(params, wN_H7H7_H7H7Mean)
      if ~isscalar(wN_H7H7_H7H7Mean) || (wN_H7H7_H7H7Mean < 0)
        disp('wN_H7H7_H7H7Mean␣not␣set,␣0␣<=␣wN_H7H7_H7H7Mean␣required.');
      else
        params.wN_H7H7_H7H7Mean= wN_H7H7_H7H7Mean;
      end
    end % wN_H7H7_H7H7Mean set function
    function params= set.dDMean(params, dDMean)
      if ~isscalar(dDMean) || (dDMean < 0)
        disp('dDMean␣not␣set,␣0␣<=␣dDMean␣required.');
      else
        params.dDMean= dDMean;
      end
    end % dDMean set function
    function params= set.H13TotalMaxMean(params, H13TotalMaxMean)
      if ~isscalar(H13TotalMaxMean) || (H13TotalMaxMean < 0) || (H13TotalMaxMean < params...
.H13TotalMinMean)
        disp('H13TotalMaxMean␣not␣set,␣0␣<=␣H13TotalMinMean␣<=␣H13TotalMaxMean␣required....
');
      else
        params.H13TotalMaxMean= H13TotalMaxMean;
      end
    end % H13TotalMaxMean set function
    function params= set.H13TotalMinMean(params, H13TotalMinMean)
      if ~isscalar(H13TotalMinMean) || (H13TotalMinMean < 0) || (params.H13TotalMaxMean <...
 H13TotalMinMean)
        disp('H13TotalMinMean␣not␣set,␣0␣<=␣H13TotalMinMean␣<=␣H13TotalMaxMean␣required....
');
      else
        params.H13TotalMinMean= H13TotalMinMean;
      end
    end % H13TotalMinMean set function
    function params= set.H13TotalHalfLifeMean(params, H13TotalHalfLifeMean)
      if ~isscalar(H13TotalHalfLifeMean) || (H13TotalHalfLifeMean <= 0)
        disp('H13TotalHalfLifeMean␣not␣set,␣0␣<␣H13TotalHalfLifeMean␣required.');
      else
        params.H13TotalHalfLifeMean= H13TotalHalfLifeMean;
      end
    end % H13TotalHalfLifeMean set function
    function params= set.gradientFunc(params, gradientFunc)
```

```
      if ~ischar(gradientFunc)
        disp('gradientFunc not set, a string is required.');
      else
        params.gradientFunc= gradientFunc;
      end
    end % gradientFunc set function
  end % Public SET methods

  methods % Public GET methods
    function timeLags= get.timeLags(params)
      if params.numGenes == 1
        timeLags= [params.tau_h7'; params.tau_H7'];
        timeLags= timeLags(:)'; % Reorder by cell
      elseif params.numGenes == 2
        timeLags= [params.tau_h7'; params.tau_H7'; params.tau_d'; params.tau_D'];
        timeLags= timeLags(:)'; % Reorder by cell
      end
    end
    function somiteGrowthRate= get.somiteGrowthRate(params)
      somiteGrowthRate= 1/params.tailbudPeriod;
    end % get somiteGrowthRate
    function cellBirthRate= get.cellBirthRate(params)
      cellBirthRate= params.cellsPerSomite*params.somiteGrowthRate;
    end % get somiteGrowthRate
    function birthTimes= get.birthTimes(params)
      birthTimes= repmat(params.timeInterval(1) + (0:params.cellArraySize(1) - 1)'/params...
.cellBirthRate, params.cellArraySize(2), 1);
    end
    function numCells= get.numCells(params)
      numCells= prod(params.cellArraySize);
    end
    function equationsPerCell= get.equationsPerCell(params)
      equationsPerCell= 2*params.numGenes;
    end
    function numEquations= get.numEquations(params)
      numEquations= params.numCells*params.equationsPerCell;
    end
    function gH7Mean= get.gH7Mean(params)
      gH7Mean= params.mRNAProductionRate*(1 + params.rhoMean)/params.rhoMean;
    end
    function gDMean= get.gDMean(params)
      gDMean= params.mRNAProductionRate*(1 + params.rhoMean)/params.rhoMean;
    end
    function couplingMatrix= get.couplingMatrix(params)
      if params.couplingBool
       % Additive nearest-neighbor (includes cornering cells)
```

```
        couplingMatrix= Params.createNearestNeighborCouplingMatrix(params.cellArraySize);
       % Alternative: Additive, axial-only, nearest-neighbor
%         couplingMatrix= Params.createAxialCouplingMatrix(params.cellArraySize);
       % Option: neighbor-averaged coupling
%         couplingMatrix= couplingMatrix./repmat(sum(couplingMatrix, 2), 1, params....
numCells);
      elseif params.numGenes == 2
        couplingMatrix= zeros(params.numCells);
      else
        couplingMatrix= [];
      end
    end
   % Indexing vectors
    function H7TotalIndices= get.H7TotalIndices(params)
      H7TotalIndices= 1:params.equationsPerCell:params.numEquations;
    end
    function h7Indices= get.h7Indices(params)
      h7Indices= 2:params.equationsPerCell:params.numEquations;
    end
    function DIndices= get.DIndices(params)
      if params.numGenes == 1
        DIndices= [];
      elseif params.numGenes == 2
        DIndices= 3:params.equationsPerCell:params.numEquations;
      end
    end
    function dIndices= get.dIndices(params)
      if params.numGenes == 1
        dIndices= [];
      elseif params.numGenes == 2
        dIndices= 4:params.equationsPerCell:params.numEquations;
      end
    end
    function H7TotalDelayedh7Indices= get.H7TotalDelayedh7Indices(params)
      if params.noiseBool
        if params.numGenes == 1
          H7TotalDelayedh7Indices= diag(repmat([true false], 1, params.numCells));
        elseif params.numGenes == 2
          H7TotalDelayedh7Indices= diag(repmat([true false false false], 1, params....
numCells));
        end
      else % No noise
        if params.numGenes == 1
          H7TotalDelayedh7Indices= repmat([true false; false false], params.numCells, 1);
        elseif params.numGenes == 2
```

```
        H7TotalDelayedh7Indices= repmat([true false false false; false false false ...
false; false false false false; false false false false], params.numCells, 1);
      end
    end
  end
  function h7DelayedH7Indices= get.h7DelayedH7Indices(params)
    if params.noiseBool
      if params.numGenes == 1
        h7DelayedH7Indices= diag(repmat([false true], 1, params.numCells));
      elseif params.numGenes == 2
        h7DelayedH7Indices= diag(repmat([false true false false], 1, params.numCells));
      end
    else % No noise
      if params.numGenes == 1
        h7DelayedH7Indices= repmat([false false; false true], params.numCells, 1);
      elseif params.numGenes == 2
        h7DelayedH7Indices= repmat([false false false false; false true false false; ...
false false false false; false false false false], params.numCells, 1);
      end
    end
  end
  function DDelayedh7Indices= get.DDelayedh7Indices(params)
    if params.noiseBool
      if params.numGenes == 1
        DDelayedh7Indices= [];
      elseif params.numGenes == 2
        DDelayedh7Indices= diag([repmat([true false false false], 1, params.numCells - ...
1), true, false], -2);
      end
    else
      if params.numGenes == 1
        DDelayedh7Indices= [];
      elseif params.numGenes == 2
        DDelayedh7Indices= repmat([false false false false; false false false false; ...
true false false false; false false false false], params.numCells, 1);
      end
    end
  end
  function H7TotalDelayeddIndices= get.H7TotalDelayeddIndices(params)
    if params.noiseBool
      if params.numGenes == 1
        H7TotalDelayeddIndices= [];
      elseif params.numGenes == 2
        H7TotalDelayeddIndices= diag([repmat([true false false false], 1, params....
numCells - 1), true, false], 2);
      end
```

```
      else
        if params.numGenes == 1
          H7TotalDelayeddIndices= [];
        elseif params.numGenes == 2
          H7TotalDelayeddIndices= repmat([false false true false; false false false false...
; false false false false; false false false false], params.numCells, 1);
        end
      end
    end
    function dDelayedDIndices= get.dDelayedDIndices(params)
      if params.noiseBool
        if params.numGenes == 1
          dDelayedDIndices= [];
        elseif params.numGenes == 2
          dDelayedDIndices= diag(repmat([false false false true], 1, params.numCells));
        end
      else
        if params.numGenes == 1
          dDelayedDIndices= [];
        elseif params.numGenes == 2
          dDelayedDIndices= repmat([false false false false; false false false false; ...
false false false false; false false false true], params.numCells, 1);
        end
      end
    end
   % Computed Parameters
    function rho= get.rho(params)
      if params.noiseBool
        rho= addNoise(params, params.rhoMean, params.rhoStream);
      else
        rho= params.rhoMean;
      end
    end
    function kH13H13= get.kH13H13(params)
      if params.noiseBool
        kH13H13= addNoise(params, params.kH13H13Mean, params.kH13H13Stream);
      else
        kH13H13= params.kH13H13Mean;
      end
    end
    function kH7H7= get.kH7H7(params)
      if params.noiseBool
        kH7H7= addNoise(params, params.kH7H7Mean, params.kH7H7Stream);
      else
        kH7H7= params.kH7H7Mean;
      end
```

```
end
function kH7H13= get.kH7H13(params)
  if params.noiseBool
    kH7H13= addNoise(params, params.kH7H13Mean, params.kH7H13Stream);
  else
    kH7H13= params.kH7H13Mean;
  end
end
function tau_h7= get.tau_h7(params)
  if params.noiseBool
    tau_h7= addNoise(params, params.tau_h7Mean, params.tau_h7Stream);
  else
    tau_h7= params.tau_h7Mean;
  end
end
function tau_H7= get.tau_H7(params)
  if params.noiseBool
    tau_H7= addNoise(params, params.tau_H7Mean, params.tau_H7Stream);
  else
    tau_H7= params.tau_H7Mean;
  end
end
function aH7= get.aH7(params)
  if params.noiseBool
    aH7= addNoise(params, params.aH7Mean, params.aH7Stream);
  else
    aH7= params.aH7Mean;
  end
end
function bH7= get.bH7(params)
  if params.noiseBool
    bH7= addNoise(params, params.bH7Mean, params.bH7Stream);
  else
    bH7= params.bH7Mean;
  end
end
function bH7H7= get.bH7H7(params)
  if params.noiseBool
    bH7H7= addNoise(params, params.bH7H7Mean, params.bH7H7Stream);
  else
    bH7H7= params.bH7H7Mean;
  end
end
function bH7H13= get.bH7H13(params)
  if params.noiseBool
    bH7H13= addNoise(params, params.bH7H13Mean, params.bH7H13Stream);
```

```
  else
    bH7H13= params.bH7H13Mean;
  end
end
function gH7= get.gH7(params)
  if params.noiseBool
    gH7= addNoise(params, params.gH7Mean, params.gH7Stream);
  else
    gH7= params.gH7Mean;
  end
end
function dH7= get.dH7(params)
  if params.noiseBool
    dH7= addNoise(params, params.dH7Mean, params.dH7Stream);
  else
    dH7= params.dH7Mean;
  end
end
function rH7H7= get.rH7H7(params)
  if params.noiseBool
    rH7H7= addNoise(params, params.rH7H7Mean, params.rH7H7Stream);
  else
    rH7H7= params.rH7H7Mean;
  end
end
function wH7H7_H7H7= get.wH7H7_H7H7(params)
  if params.noiseBool
    wH7H7_H7H7= addNoise(params, params.wH7H7_H7H7Mean, params.wH7H7_H7H7Stream);
  else
    wH7H7_H7H7= params.wH7H7_H7H7Mean;
  end
end
function tau_d= get.tau_d(params)
  if params.noiseBool
    tau_d= addNoise(params, params.tau_dMean, params.tau_dStream);
  else
    tau_d= params.tau_dMean;
  end
end
function tau_D= get.tau_D(params)
  if params.noiseBool
    tau_D= addNoise(params, params.tau_DMean, params.tau_DStream);
  else
    tau_D= params.tau_DMean;
  end
end
```

```
function aD= get.aD(params)
  if params.noiseBool
    aD= addNoise(params, params.aDMean, params.aDStream);
  else
    aD= params.aDMean;
  end
end
function bD= get.bD(params)
  if params.noiseBool
    bD= addNoise(params, params.bDMean, params.bDStream);
  else
    bD= params.bDMean;
  end
end
function gD= get.gD(params)
  if params.noiseBool
    gD= addNoise(params, params.gDMean, params.gDStream);
  else
    gD= params.gDMean;
  end
end
function dD= get.dD(params)
  if params.noiseBool
    dD= addNoise(params, params.dDMean, params.dDStream);
  else
    dD= params.dDMean;
  end
end
function rN= get.rN(params)
  if params.noiseBool
    rN= addNoise(params, params.rNMean, params.rNStream);
  else
    rN= params.rNMean;
  end
end
function wN= get.wN(params)
  if params.noiseBool
    wN= addNoise(params, params.wNMean, params.wNStream);
  else
    wN= params.wNMean;
  end
end
function wN_H7H7= get.wN_H7H7(params)
  if params.noiseBool
    wN_H7H7= addNoise(params, params.wN_H7H7Mean, params.wN_H7H7Stream);
  else
```

```
        wN_H7H7= params.wN_H7H7Mean;
      end
    end
    function wN_H7H7_H7H7= get.wN_H7H7_H7H7(params)
      if params.noiseBool
        wN_H7H7_H7H7= addNoise(params, params.wN_H7H7_H7H7Mean, params.wN_H7H7_H7H7Stream...
);
      else
        wN_H7H7_H7H7= params.wN_H7H7_H7H7Mean;
      end
    end
    function H13TotalMax= get.H13TotalMax(params)
      if params.noiseBool
        H13TotalMax= addNoise(params, params.H13TotalMaxMean, params.H13TotalMaxStream);
      else
        H13TotalMax= params.H13TotalMaxMean;
      end
    end
    function H13TotalMin= get.H13TotalMin(params)
      if params.noiseBool
        H13TotalMin= addNoise(params, params.H13TotalMinMean, params.H13TotalMinStream);
      else
        H13TotalMin= params.H13TotalMinMean;
      end
    end
    function H13TotalHalfLife= get.H13TotalHalfLife(params)
      if params.noiseBool
        H13TotalHalfLife= addNoise(params, params.H13TotalHalfLifeMean, params....
H13TotalHalfLifeStream);
      else
        H13TotalHalfLife= params.H13TotalHalfLifeMean;
      end
    end

   % addNoise method
    function paramVec= addNoise(params, paramMean, paramStream)
      paramVec= zeros(params.numCells, 1);
      if 0 < paramMean % Only add noise to (strictly) positive parameters
        paramStream.reset; % Makes noise repeatable
        if strcmpi(params.noiseType, 'uniform')
          paramVec= paramMean*(1 + (-params.noiseProportionOfMean + 2*params....
noiseProportionOfMean*(rand(paramStream, size(paramVec)))));
        else % "positive" normal
          unconvergedIndices= (paramVec <= 0); % Non-negativity constraint
          while any(unconvergedIndices(:))
           % Note: 99.7% within +/- noiseProportionOfMean of mean value
```

```
            paramVec(unconvergedIndices)= paramMean*(1 + params.noiseProportionOfMean/3*...
randn(paramStream, size(find(unconvergedIndices))));
            unconvergedIndices= (paramVec <= 0);
          end
        end
      end
    end % addNoise method

    % DISP method
    function disp(params)
      if params.noiseBool
        disp('noiseBool:␣true');
      else
        disp('noiseBool:␣false');
      end
      disp([ 'noiseType:␣' params.noiseType ]);
      disp([ 'noiseSeed:␣' num2str(params.noiseSeed) ]);
      disp([ 'noiseProportionOfMean:␣' num2str(params.noiseProportionOfMean) ]);
      disp([ 'timeInterval:␣' mat2str(params.timeInterval) ]);
      disp([ 'settlingTime:␣' num2str(params.settlingTime) ]);
      disp([ 'timeLags:␣' mat2str(params.timeLags) ]);
      if isstruct(params.history)
        disp('history:␣'); disp(params.history);
      elseif isa(params.history, 'function_handle')
        disp([ 'history:␣@' func2str(params.history) ]);
      elseif isvector(params.history)
        disp([ 'history:␣' mat2str(params.history) ]);
      end
      if isempty(params.ddeOptions)
        disp('ddeOptions:␣[]');
      else
        disp('ddeOptions:␣'); disp(params.ddeOptions);
      end
      if(params.fastModeBool)
        disp('fastMode:␣true');
      else
        disp('fastMode:␣false');
      end
      disp([ 'cellsPerSomite:␣' num2str(params.cellsPerSomite) ]);
      disp([ 'somitesInPSM:␣' num2str(params.somitesInPSM) ]);
      disp([ 'tailbudPeriod:␣' num2str(params.tailbudPeriod) ]);
      disp([ 'somiteGrowthRate:␣' num2str(params.somiteGrowthRate) ]);
      disp([ 'cellBirthRate:␣' num2str(params.cellBirthRate) ]);
      disp([ 'birthTimes:␣' mat2str(params.birthTimes') ]);
      disp([ 'cellArraySize:␣' mat2str(params.cellArraySize) ]);
      disp([ 'numCells:␣' num2str(params.numCells) ]);
```

```
disp([ 'numGenes:␣' num2str(params.numGenes) ]);
disp([ 'equationsPerCell:␣' num2str(params.equationsPerCell) ]);
disp([ 'numEquations:␣' num2str(params.numEquations) ]);
disp([ 'gradientFunc:␣' params.gradientFunc ]);
disp([ 'H13TotalMaxMean:␣' num2str(params.H13TotalMaxMean) ]);
disp([ 'H13TotalMinMean:␣' num2str(params.H13TotalMinMean) ]);
disp([ 'H13TotalHalfLifeMean:␣' num2str(params.H13TotalHalfLifeMean) ]);
disp([ 'rhoMean:␣' num2str(params.rhoMean) ]);
disp([ 'numBindingSites:␣' num2str(params.numBindingSites) ]);
disp([ 'tau_h7Mean:␣' num2str(params.tau_h7Mean) ]);
disp([ 'tau_H7Mean:␣' num2str(params.tau_H7Mean) ]);
disp([ 'aH7Mean:␣' num2str(params.aH7Mean) ]);
disp([ 'bH7Mean:␣' num2str(params.bH7Mean) ]);
disp([ 'bH7H7Mean:␣' num2str(params.bH7H7Mean) ]);
disp([ 'bH7H13Mean:␣' num2str(params.bH7H13Mean) ]);
disp([ 'gH7Mean:␣' num2str(params.gH7Mean) ]);
disp([ 'dH7Mean:␣' num2str(params.dH7Mean) ]);
disp([ 'rH7H7Mean:␣' num2str(params.rH7H7Mean) ]);
disp([ 'wH7H7_H7H7Mean:␣' num2str(params.wH7H7_H7H7Mean) ]);
disp([ 'kH13H13Mean:␣' num2str(params.kH13H13Mean) ]);
disp([ 'kH7H7Mean:␣' num2str(params.kH7H7Mean) ]);
disp([ 'kH7H13Mean:␣' num2str(params.kH7H13Mean) ]);
disp([ 'tau_dMean:␣' num2str(params.tau_dMean) ]);
disp([ 'tau_DMean:␣' num2str(params.tau_DMean) ]);
disp([ 'aDMean:␣' num2str(params.aDMean) ]);
disp([ 'bDMean:␣' num2str(params.bDMean) ]);
disp([ 'gDMean:␣' num2str(params.gDMean) ]);
disp([ 'dDMean:␣' num2str(params.dDMean) ]);
disp([ 'rNMean:␣' num2str(params.rNMean) ]);
disp([ 'wNMean:␣' num2str(params.wNMean) ]);
disp([ 'wN_H7H7Mean:␣' num2str(params.wN_H7H7Mean) ]);
disp([ 'wN_H7H7_H7H7Mean:␣' num2str(params.wN_H7H7_H7H7Mean) ]);
if(params.couplingBool)
  disp('couplingBool:␣true');
else
  disp('couplingBool:␣false');
end
if isempty(params.couplingMatrix)
  disp('couplingMatrix:␣[]');
else
  [M N]= size(params.couplingMatrix);
  if (M > 2) || (N > 2)
    disp([ 'couplingMatrix:␣' num2str(M) '␣x␣' num2str(N) '␣matrix']);
  else
    disp('couplingMatrix:␣'); disp(params.couplingMatrix);
  end
```

```
      end
      disp([ 'H7TotalIndices:␣' mat2str(params.H7TotalIndices) ]);
      disp([ 'h7Indices:␣' mat2str(params.h7Indices) ]);
      disp([ 'DIndices:␣' mat2str(params.DIndices) ]);
      disp([ 'dIndices:␣' mat2str(params.dIndices) ]);
      [M N]= size(params.H7TotalDelayedh7Indices);
      if (M == 0) && (N == 0)
        disp('H7TotalDelayedh7Indices:␣[]');
        disp('h7DelayedH7Indices:␣[]');
        disp('DDelayedh7Indices:␣[]');
        disp('H7TotalDelayeddIndices:␣[]');
        disp('dDelayedDIndices:␣[]');
      elseif (M > 8) || (N > 8)
        disp([ 'H7TotalDelayedh7Indices:␣' num2str(M) '␣x␣' num2str(N) '␣matrix']);
        disp([ 'h7DelayedH7Indices:␣' num2str(M) '␣x␣' num2str(N) '␣matrix']);
        disp([ 'DDelayedh7Indices:␣' num2str(M) '␣x␣' num2str(N) '␣matrix']);
        disp([ 'H7TotalDelayeddIndices:␣' num2str(M) '␣x␣' num2str(N) '␣matrix']);
        disp([ 'dDelayedDIndices:␣' num2str(M) '␣x␣' num2str(N) '␣matrix']);
      else
        disp('H7TotalDelayedh7Indices:␣'); disp(params.H7TotalDelayedh7Indices);
        disp('h7DelayedH7Indices:␣'); disp(params.h7DelayedH7Indices);
        disp('DDelayedh7Indices:␣'); disp(params.DDelayedh7Indices);
        disp('H7TotalDelayeddIndices:␣'); disp(params.H7TotalDelayeddIndices);
        disp('dDelayedDIndices:␣'); disp(params.dDelayedDIndices);
      end
    end % disp
  end % Public GET methods

  methods (Access = 'private') % Access by class members only
    function params= initStreams(params)
      [params.rhoStream, params.kH13H13Stream, params.kH7H7Stream, params.kH7H13Stream, ...
params.tau_h7Stream, params.tau_H7Stream, params.gH7Stream, params.aH7Stream, params....
bH7Stream, params.bH7H7Stream, params.bH7H13Stream, params.dH7Stream, params.rH7H7Stream,...
 params.wH7H7_H7H7Stream, params.tau_dStream, params.tau_DStream, params.gDStream, params...
.aDStream, params.bDStream, params.dDStream, params.rNStream, params.wNStream, params....
wN_H7H7Stream, params.wN_H7H7_H7H7Stream, params.H13TotalMaxStream, params....
H13TotalMinStream, params.H13TotalHalfLifeStream]= RandStream.create('mrg32k3a', '...
NumStreams',27, 'Seed',params.noiseSeed);
    end
  end % Private methods

  methods (Static)
    function couplingMatrix= createNearestNeighborCouplingMatrix(cellArraySize)
    % Note: Works for 1D vectors and 2D arrays.
    % Note: DeltaC assumed to be evenly distributed through cell membrane
    %  to adjacent cells without Notch receptor saturation. This scaling
```

```
% is lumped into the rN parameter.

  M= cellArraySize(1);
  N= cellArraySize(2);
  K= M*N;
  couplingMatrix= zeros(K);

  for k= 1:K
    if k == 1 % Top-left corner
      if M > 1
        couplingMatrix(1,2)= 1;
        if N > 1
          couplingMatrix(1,M+1:M+2)= 1;
        end
      elseif N > 1
        couplingMatrix(1,M+1)= 1;
      end
    elseif (2 <= k) && (k <= M-1) % Left side
      couplingMatrix(k,[k-1,k+1])= 1;
      if N > 1
        couplingMatrix(k,k+M-1:k+M+1)= 1;
      end
    elseif k == M % Bottom-left corner
      couplingMatrix(M,k-1)= 1;
      if N > 1
        couplingMatrix(M,k+M-1:k+M)= 1;
      end
    elseif (rem(k, M) == 1) && (k < K-M+1) % Top side
      couplingMatrix(k,[k-M:k-M+1, k+1, k+M:k+M+1])= 1;
    elseif (rem(k, M) == 0) && (k < K) % Bottom side
      if M > 1
        couplingMatrix(k,[k-M-1:k-M, k-1, k+M-1:k+M])= 1;
      else
        couplingMatrix(k,[k-M, k+M])= 1;
      end
    elseif k == K-M+1 % Top-right corner
      if M > 1
        couplingMatrix(k,[k-M:k-M+1, k+1])= 1;
      else
        couplingMatrix(k,k-M)= 1;
      end
    elseif (K-M+1 < k) && (k < K) % Right side
      couplingMatrix(k,[k-M-1:k-M+1, k-1, k+1])= 1;
    elseif k == K % Bottom-right corner
      couplingMatrix(k,[k-M-1:k-M, k-1])= 1;
    else % Inside
```

```
        couplingMatrix(k,[k-M-1:k-M+1, k-1, k+1, k+M-1:k+M+1])= 1;
      end
    end
  end % createNearestNeighborCouplingMatrix()

  function couplingMatrix= createAxialCouplingMatrix(cellArraySize)
  % Note: Works for 1D vectors and 2D arrays.
  % Note: DeltaC assumed to be evenly distributed through cell membrane
  %  to adjacent cells without Notch receptor saturation. This scaling
  %  is lumped into the rN parameter.

    M= cellArraySize(1);
    N= cellArraySize(2);
    K= M*N;
    couplingMatrix= zeros(K);

    for k= 1:K
      if k == 1 % Top-left corner
        if M > 1
          couplingMatrix(1,2)= 1;
        end
      elseif (2 <= k) && (k <= M-1) % Left side
        couplingMatrix(k,[k-1, k+1])= 1;
      elseif k == M % Bottom-left corner
        couplingMatrix(M,k-1)= 1;
      elseif (rem(k, M) == 1) && (k < K-M+1) % Top side
        couplingMatrix(k,k+1)= 1;
      elseif (rem(k, M) == 0) && (k < K) % Bottom side
        if M > 1
          couplingMatrix(k,k-1)= 1;
        end
      elseif k == K-M+1 % Top-right corner
        if M > 1
          couplingMatrix(k,k+1)= 1;
        end
      elseif (K-M+1 < k) && (k < K) % Right side
        couplingMatrix(k,[k-1, k+1])= 1;
      elseif k == K % Bottom-right corner
        couplingMatrix(k,k-1)= 1;
      else % Inside
        couplingMatrix(k,[k-1, k+1])= 1;
      end
    end
  end % createAxialCouplingMatrix()
end % Static methods
```

```
end % classdef
```

1.1.3. `calcDimerization.m`. This implements a simple iterative numerical scheme for calculating monomer, homodimer, and heterodimer amounts from total clock protein and total control protein amounts.

```
function [H13, H7, H13H13, H7H7, H7H13]= ...
  calcDimerization(H13Total, H7Total, kH13H13, kH7H7, kH7H13)

% Usage:
% [H13, H7, H13H13, H7H7, H7H13]= calcDimerization(H13Total, H7Total, kH13H13, kH7H7, ...
kH7H13)
%
% Computes (via iteration) the nonlinear algebraic system of equations for
%  the fast dimerization.
% 2D Vectorized - assumes H7Total & H13Total arrays have same size and
%  are non-negative:
%   1) The first (row) dimension is the total number of cells.
%   2) The second (column) dimension is typically represents a time-series.
% The kappa parameters may be all scalars or all column vectors with
%  the same number of rows as H7Total & H13Total.

%tic
  residualTol= 1e-6;
  promptFlag= true;
  solution= cat(3, H13Total, H7Total); % IC for H13 and H7 iterative map
  arraySize= size(H13Total); % Same as size(H7Total)
  unconvergedIndices= true(arraySize); % For stopping criteron

  if ~isscalar(kH13H13) && (arraySize(2) > 1) % Heterogeneous cell population
    kH7H7= repmat(kH7H7, 1, arraySize(2));
    kH13H13= repmat(kH13H13, 1, arraySize(2));
    kH7H13= repmat(kH7H13, 1, arraySize(2));
  end

  k= 0; % Iteration counter
  while any(unconvergedIndices(:)) % Iteration loop
    k= k + 1;
    if promptFlag && ~rem(k,1000)
      while true % User input loop
        response= menu(['No convergence to root after ' num2str(k) ' iterations. Iterate...
 further? '], 'Yes', 'Yes, turn off prompting', 'No, terminate execution');
        switch response
          case 1
            break;
          case 2
            promptFlag= false;
            break;
```

```
            case 3
              disp('Parameter Dump:');
              disp([ 'H13Total= ' mat2str(H13Total) ]);
              disp([ 'H7Total= ' mat2str(H7Total) ]);
              disp([ 'kH13H13= ' mat2str(kH13H13) ]);
              disp([ 'kH7H7= ' mat2str(kH7H7) ]);
              disp([ 'kH7H13= ' mat2str(kH7H13) ]);
              error('Failed to converge to root.');
            otherwise
              continue;
          end
        end
      end
     % Iterate the map
      solution= cat(3, ...
(sqrt((kH13H13.*(1 + solution(:,:,2)./kH7H13)).^2 + 8*kH13H13.*H13Total) - kH13H13.*(1 + ...
solution(:,:,2)./kH7H13))/4, ...
(sqrt((kH7H7.*(1 + solution(:,:,1)./kH7H13)).^2 + 8*kH7H7.*H7Total) - kH7H7.*(1 + ...
solution(:,:,1)./kH7H13))/4);
     % Use component-wise sup norm residual to compute unconverged indices
      unconvergedIndices= ...
(abs(2./kH13H13.*solution(:,:,1).^2 + (1 + solution(:,:,2)./kH7H13).*solution(:,:,1) - ...
H13Total) >= residualTol) | ...
(abs(2./kH7H7.*solution(:,:,2).^2 + (1 + solution(:,:,1)./kH7H13).*solution(:,:,2) - ...
H7Total) >= residualTol);
  end

 % Assign monomers to output variables
  H13= solution(:,:,1);
  H7= solution(:,:,2);
 % Compute dimers
  H7H13= H7.*H13./kH7H13;
  H13H13= (H13Total - H13 - H7H13)/2; % Faster than H13H13= H13.^2./kH13H13
  H7H7= (H7Total - H7 - H7H13)/2; % Faster than H7H7= H7.^2./kH7H7
%toc
%  k
end
```

1.1.4. `calcDimerizationNewton2Cell.m`. This implements Newton's method for calculating monomer, homodimer, and heterodimer amounts from total clock protein and total control protein amounts. It can replace the function `calcDimerization.m` in `somitogenesis.m` for two-cell only simulations where speed is very important, such as in parameter estimation.

```
function [H13, H7, H13H13, H7H7, H7H13]= ...
  calcDimerizationNewton2Cell(H13Total, H7Total, kH13H13, kH7H7, kH7H13)

% Usage:
```

```
%  [H13, H7, H13H13, H7H7, H7H13]= calcDimerizationNewton2Cell(H13Total, H7Total, kH13H13...
, kH7H7, kH7H13)
%
% Computes (via Newton iteration) the nonlinear algebraic system of equations for the ...
fast dimerization.
% H13Total and H7Total should be 2 x 1 vectors and kH13H13, kH7H7, and kH7H13 should be ...
scalars.

%tic
  residualTol= 1e-6;
  promptFlag= true;
  solutionCell1= [H13Total(1); H7Total(1)]/2; % IC Cell 1
  solutionCell2= [H13Total(2); H7Total(2)]/2; % IC Cell 2
  unconvergedIndices= true(4,1); % For stopping criteron

%  if ~isscalar(kH13H13) && (arraySize(2) > 1) % Heterogeneous cell population
%    kH7H7= repmat(kH7H7, 1, arraySize(2));
%    kH13H13= repmat(kH13H13, 1, arraySize(2));
%    kH7H13= repmat(kH7H13, 1, arraySize(2));
%    error('Heterogeneous cells not yet supported.');
%  end

  k= 0; % Iteration counter
  while any(unconvergedIndices(:)) % Iteration loop
    k= k + 1;
    if promptFlag && ~rem(k,1000)
      while true % User input loop
        response= menu(['No convergence to root after ' num2str(k) ' iterations. Iterate...
 further? '], 'Yes', 'Yes, turn off prompting', 'No, terminate execution');
        switch response
          case 1
            break;
          case 2
            promptFlag= false;
            break;
          case 3
            disp('Parameter Dump:');
            disp([ 'H13Total= ' mat2str(H13Total) ]);
            disp([ 'H7Total= ' mat2str(H7Total) ]);
            disp([ 'kH13H13= ' mat2str(kH13H13) ]);
            disp([ 'kH7H7= ' mat2str(kH7H7) ]);
            disp([ 'kH7H13= ' mat2str(kH7H13) ]);
            error('Failed to converge to root.');
          otherwise
            continue;
        end
```

```
      end
    end
   % Iterate the map: solution= solution - Dinv(solution)*F(solution);
    solutionCell1= solutionCell1 - ...
[4/kH7H7*solutionCell1(2) + 1 + solutionCell1(1)/kH7H13, -solutionCell1(1)/kH7H13;
 -solutionCell1(2)/kH7H13, 4/kH13H13*solutionCell1(1) + 1 + solutionCell1(2)/kH7H13]/...
 ((4/kH13H13*solutionCell1(1) + 1 + solutionCell1(2)/kH7H13)*(4/kH7H7*solutionCell1(2) + ...
...
 1 + solutionCell1(1)/kH7H13) - solutionCell1(1)*solutionCell1(2)/kH7H13^2)*...
[2/kH13H13*solutionCell1(1).^2 + (1 + solutionCell1(2)/kH7H13).*solutionCell1(1) - ...
H13Total(1);
 2/kH7H7*solutionCell1(2).^2 + (1 + solutionCell1(1)/kH7H13).*solutionCell1(2) - H7Total...
(1)];
    solutionCell2= solutionCell2 - ...
[4/kH7H7*solutionCell2(2) + 1 + solutionCell2(1)/kH7H13, -solutionCell2(1)/kH7H13;
 -solutionCell2(2)/kH7H13, 4/kH13H13*solutionCell2(1) + 1 + solutionCell2(2)/kH7H13]/...
 ((4/kH13H13*solutionCell2(1) + 1 + solutionCell2(2)/kH7H13)*(4/kH7H7*solutionCell2(2) + ...
...
 1 + solutionCell2(1)/kH7H13) - solutionCell2(1)*solutionCell2(2)/kH7H13^2)*...
[2/kH13H13*solutionCell2(1)^2 + (1 + solutionCell2(2)/kH7H13)*solutionCell2(1) - H13Total...
(2);
 2/kH7H7*solutionCell2(2)^2 + (1 + solutionCell2(1)/kH7H13)*solutionCell2(2) - H7Total(2)...
];

%    solution= solution - ...
%[[4/kH7H7*solution(2) + 1 + solution(1)/kH7H13, -solution(1)/kH7H13;
%  -solution(2)/kH7H13, 4/kH13H13*solution(1) + 1 + solution(2)/kH7H13]/...
%   ((4/kH13H13*solution(1) + 1 + solution(2)/kH7H13)*(4/kH7H7*solution(2) + ...
%   1 + solution(1)/kH7H13) - solution(1)*solution(2)/kH7H13^2), [0 0; 0 0];
% [0 0; 0 0], [4/kH7H7*solution(4) + 1 + solution(3)/kH7H13, -solution(3)/kH7H13;
%  -solution(4)/kH7H13, 4/kH13H13*solution(3) + 1 + solution(4)/kH7H13]/...
%   ((4/kH13H13*solution(3) + 1 + solution(4)/kH7H13)*(4/kH7H7*solution(4) + ...
%   1 + solution(3)/kH7H13) - solution(3)*solution(4)/kH7H13^2)]*...
%[2/kH13H13*solution(1).^2 + (1 + solution(2)/kH7H13).*solution(1) - H13Total(1);
% 2/kH7H7*solution(2).^2 + (1 + solution(1)/kH7H13).*solution(2) - H7Total(1);
% 2/kH13H13*solution(3)^2 + (1 + solution(4)/kH7H13)*solution(3) - H13Total(2);
% 2/kH7H7*solution(4)^2 + (1 + solution(3)/kH7H13)*solution(4) - H7Total(2)];

   % Use component-wise sup norm residual to compute unconverged indices
    unconvergedIndices= abs(...
[2/kH13H13*solutionCell1(1)^2 + (1 + solutionCell1(2)./kH7H13)*solutionCell1(1) - ...
H13Total(1);
 2/kH7H7*solutionCell1(2)^2 + (1 + solutionCell1(1)/kH7H13)*solutionCell1(2) - H7Total(1)...
;
 2/kH13H13*solutionCell2(1)^2 + (1 + solutionCell2(2)./kH7H13)*solutionCell2(1) - ...
H13Total(2);
```

```
 2/kH7H7*solutionCell2(2)^2 + (1 + solutionCell2(1)/kH7H13)*solutionCell2(2) - H7Total(2)...
])  ...
>= residualTol;

%    unconvergedIndices= ...
%(abs(2./kH13H13.*solution([1 3]).^2 + (1 + solution([2 4])./kH7H13).*solution([1 3]) - ...
H13Total) >= residualTol) | ...
%(abs(2./kH7H7.*solution([2 4]).^2 + (1 + solution([1 3])./kH7H13).*solution([2 4]) - ...
H7Total) >= residualTol);
  end

 % Assign monomers to output variables
  H13= [solutionCell1(1); solutionCell2(1)];
  H7= [solutionCell1(2); solutionCell2(2)];
 % Compute dimers
  H7H13= H7.*H13./kH7H13;
  H13H13= (H13Total - H13 - H7H13)/2; % Faster than H13H13= H13.^2./kH13H13
  H7H7= (H7Total - H7 - H7H13)/2; % Faster than H7H7= H7.^2./kH7H7
%toc
%  k
end
```

1.1.5. `calcDimerizationNewton.m`. This implements Newton's method for calculating monomer, homodimer, and heterodimer amounts from total clock protein and total control protein amounts in a single cell. It is **not** vectorized for use in `somitogenesis.m`.

```
function [H13, H7, H13H13, H7H7, H7H13]= ...
  calcDimerizationNewton(H13Total, H7Total, kH13H13, kH7H7, kH7H13)

% Usage:
%  [H13, H7, H13H13, H7H7, H7H13]= calcDimerizationNewton(H13Total, H7Total, kH13H13, ...
kH7H7, kH7H13)
%
% Computes (via Newton iteration) the nonlinear algebraic system of equations for the ...
fast dimerization.
% All inputs must be scalar.

%tic
  residualTol= 1e-6;
  promptFlag= true;

 % System vector
  F= @(solution) ...
[2/kH13H13*solution(1)^2 + (1 + solution(2)/kH7H13)*solution(1) - H13Total;
 2/kH7H7*solution(2)^2 + (1 + solution(1)/kH7H13)*solution(2) - H7Total];

 % Inverse of Jacobian
```

```
  Dinv= @(solution) ...
[4/kH7H7*solution(2) + 1 + solution(1)/kH7H13, -solution(1)/kH7H13;
 -solution(2)/kH7H13, 4/kH13H13*solution(1) + 1 + solution(2)/kH7H13]/...
 ((4/kH13H13*solution(1) + 1 + solution(2)/kH7H13)*(4/kH7H7*solution(2) + ...
 1 + solution(1)/kH7H13) - solution(1)*solution(2)/kH7H13^2);

%  solution= cat(3, H13Total, H7Total); % IC for H13 and H7 iterative map
  solution= [H13Total/2; H7Total/2];
  inputSize= size(H13Total); % Same as size(H7Total)
  unconvergedIndices= true(inputSize); % For stopping criteron

  if ~isscalar(kH13H13) && (arraySize(2) > 1) % Heterogeneous cell population
    kH7H7= repmat(kH7H7, 1, arraySize(2));
    kH13H13= repmat(kH13H13, 1, arraySize(2));
    kH7H13= repmat(kH7H13, 1, arraySize(2));
    error('Heterogeneous cells not yet supported.');
  end

  k= 0; % Iteration counter
  while any(unconvergedIndices(:)) % Iteration loop
    k= k + 1;
    if promptFlag && ~rem(k,1000)
      while true % User input loop
        response= menu(['No convergence to root after ' num2str(k) ' iterations. Iterate...
 further? '], 'Yes', 'Yes, turn off prompting', 'No, terminate execution');
        switch response
          case 1
            break;
          case 2
            promptFlag= false;
            break;
          case 3
            disp('Parameter Dump:');
            disp([ 'H13Total= ' mat2str(H13Total) ]);
            disp([ 'H7Total= ' mat2str(H7Total) ]);
            disp([ 'kH13H13= ' mat2str(kH13H13) ]);
            disp([ 'kH7H7= ' mat2str(kH7H7) ]);
            disp([ 'kH7H13= ' mat2str(kH7H13) ]);
            error('Failed to converge to root.');
          otherwise
            continue;
        end
      end
    end
   % Iterate the map
    solution= solution - Dinv(solution)*F(solution);
```

```
   % Use component-wise sup norm residual to compute unconverged indices
    unconvergedIndices= ...
(abs(2./kH13H13.*solution(1).^2 + (1 + solution(2)./kH7H13).*solution(1) - H13Total) >= ...
residualTol) | ...
(abs(2./kH7H7.*solution(2).^2 + (1 + solution(1)./kH7H13).*solution(2) - H7Total) >= ...
residualTol);
  end

 % Assign monomers to output variables
  H13= solution(1);
  H7= solution(2);
 % Compute dimers
  H7H13= H7.*H13./kH7H13;
  H13H13= (H13Total - H13 - H7H13)/2; % Faster than H13H13= H13.^2./kH13H13
  H7H7= (H7Total - H7 - H7H13)/2; % Faster than H7H7= H7.^2./kH7H7
%toc
%  k
end
```

1.1.6. `calcDimerizationFzero.m`. This implements MATLAB's `fzero` function for calculating monomer, homodimer, and heterodimer amounts from total clock protein and total control protein amounts. It is used primarily for comparison and testing purposes.

```
function [H13, H7, H13H13, H7H7, H7H13]= calcDimerizationFzero(H13Total, H7Total, kH13H13...
, kH7H7, kH7H13)

% Usage:
%  [H13, H7, H13H13, H7H7, H7H13]= calcDimerizationFzero(H13Total, H7Total, kH13H13, ...
kH7H7, kH7H13)
%
% Computes (via fzero) the nonlinear algebraic system of equations for the fast ...
dimerization.
% H13Total and H7Total inputs may be 2D vectors, kH13H13, kH7H7, and kH7H13 should be ...
scalars.

%tic
%  options= optimset(optimset('fzero'), 'TolX',10e-6);
  arraySize= size(H13Total); % Same as size(H7Total)

  if ~isscalar(kH13H13) && (arraySize(2) > 1) % Heterogeneous cell population
    kH7H7= repmat(kH7H7, 1, arraySize(2));
    kH13H13= repmat(kH13H13, 1, arraySize(2));
    kH7H13= repmat(kH7H13, 1, arraySize(2));
    error('Heterogeneous␣cells␣not␣yet␣supported.');
  end
```

```
  H7= zeros(arraySize); % Pre-allocate

 % G is soved in terms of C using one equation and substituted into the
 %  other equation, which is solved for C using fzero.
  for ii= 1:arraySize(1)
    for jj= 1:arraySize(2)
      H7FunH= @(x) 2/kH7H7*x^2 + (1 + ...
kH13H13/4*(sqrt((1 + x/kH7H13)^2 + 8/kH13H13*H13Total(ii,jj)) - ...
(1 + x/kH7H13))/kH7H13)*x - H7Total(ii,jj);
      H7(ii,jj)= fzero(H7FunH, H7Total(ii,jj)/2);
    end
  end

 % Compute other monomer
  H13= kH13H13/4*(sqrt((1 + H7/kH7H13).^2 + 8/kH13H13*H13Total) - ...
(1 + H7/kH7H13));
 % Compute dimers
  H7H13= H7.*H13./kH7H13;
  H13H13= (H13Total - H13 - H7H13)/2; % Faster than H13H13= H13.^2./kH13H13
  H7H7= (H7Total - H7 - H7H13)/2; % Faster than H7H7= H7.^2./kH7H7
%toc
end
```

1.1.7. `calcPerMedAmp.m`. This computes the component-wise period, median, and amplitude of simply periodic or steady-state solutions computed by `somitogenesis.m`.

```
function [periods, medians, amplitudes]= calcPerMedAmp(sol)

 % Usage:
 %  [periods, medians, amplitudes]= calcPerMedAmp(sol)
 %
 % Calculate periods, medians, and amplitudes of simply periodic
 %  (including equilibrated) state variables in the
 %  dde23()/ddsd() solution sol.
 % This algorithm assumes it is analyzing (in each component of
 %  sol.y) a time series reaching an equilibrium or a simply
 %  periodic behavior with at least three internal peak-to-peak
 %  oscillations (two internal max and two internal min, in
 %  either alternating order).
 % Simply periodic time series have isolated extrema, with a
 %  single maximum and a single minimum in each fundamental
 %  period.
 % Assumes (& requires) at least three data points in sol.x.
 % Equilibriated state variables are assigned zero period,
 %  although in certain situations an equilibrium may be
 %  represented by a periodic solution with very small amplitude,
 %  in which case the median estimates the equilibrium.
```

```
% Tolerances (componentwise)
  relTol= 1e-2; % Relative tolerance for detection of equilibrium
  absTol= 1e-2; % Absolute tolerance for detection of equilibrium
  tol= 1e-2; % Relative tolerance for detection of periodic signal

  [M N]= size(sol.y); % Record dimensions of time-series
  minMask= zeros(1, N); % Pre-allocate
  maxMask= minMask; % Pre-allocate
% Default values (NaN)
  periods= nan(M, 1); % Protein(s) & mRNA(s) for each cell
  medians= periods;
  amplitudes= medians;

  tailTime= sol.params.settlingTime/5; % Somewhat arbitrary
  tailStartIndex= find((sol.x(end) - tailTime) <= sol.x, 1);
  if isempty(tailStartIndex)
    tailStartIndex= 1; % Use whole time-series
  end

% TBD: Vectorize for loop?
  for k= 1:M
  % Check first for an equilibrium
    tailMean= mean(sol.y(k,tailStartIndex:end));
    if (tailMean ~= 0)
      if all(abs((sol.y(k,tailStartIndex:end) - tailMean)/tailMean) < relTol)
        periods(k)= 0;
        medians(k)= tailMean;
        amplitudes(k)= 0;
        continue;
      end
    elseif all(abs(sol.y(k,tailStartIndex:end)) < absTol)
      periods(k)= 0;
      medians(k)= 0;
      amplitudes(k)= 0;
      continue;
    end
  % Not an equilibrium, or equilibrium not sufficiently
  %  "reached", so check for a simply periodic component
  % Note: Assumes that neither minMask nor maxMask matrices are
  %  empty, and the isolated extrema assumption is key to the
  %  following detection logic.
   % Find local minima
    minMask(2:end-1)= (sol.y(k,1:end-2) >= sol.y(k,2:end-1)) & (sol.y(k,2:end-1) <= sol.y...
(k,3:end));
    minMask(1)= sol.y(k,1) <= sol.y(k,2);
```

```
    minMask(end)= sol.y(k,end-1) >= sol.y(k,end);
    minIndices= find(minMask);
   % Find local maxima
    maxMask(2:end-1)= sol.y(k,1:end-2) <= sol.y(k,2:end-1) & sol.y(k,2:end-1) >= sol.y(k...
,3:end);
    maxMask(1)= sol.y(k,1) <= sol.y(k,2);
    maxMask(end)= sol.y(k,end-1) <= sol.y(k,end);
    maxIndices= find(maxMask);
   % Clear any initial constant artifact from constant history
    for i= 1:min(length(minIndices), length(maxIndices))
      if minIndices(i) ~= maxIndices(i)
        minIndices= minIndices(i:end);
        maxIndices= maxIndices(i:end);
        break;
      end
    end
   % Check for at least 1.5 oscillations for analysis
    if isempty(minIndices) || isempty(maxIndices)
%       disp( ['Omega limit set not found in component ' num2str(k) '; equilibrium?'] );
      continue;
    else
      numEdgeMinima= (minIndices(1) < maxIndices(1)) + (minIndices(end) == N);
      numEdgeMaxima= (maxIndices(1) < minIndices(1)) + (maxIndices(end) == N);
    end
    if ((length(minIndices) - numEdgeMinima <= 1) && (length(maxIndices) - numEdgeMaxima ...
<= 1)) || (minIndices(end) == maxIndices(end))
%       disp( ['Omega limit set not found in component ' num2str(k) '; equilibrium?'] );
    else % Oscillatory - analyze using last 1.5 oscillations
      if minIndices(end) == N % Ignore any last minimum at end
        lastMinIndex= minIndices(end-1);
        lastMaxIndex= maxIndices(end);
        penultimateMaxIndex= maxIndices(end-1);
      elseif maxIndices(end) == N % Ignore any last maximum at end
        lastMaxIndex= maxIndices(end-1);
        lastMinIndex= minIndices(end);
        penultimateMinIndex= minIndices(end-1);
      else % TBD: This doesn't happen?
        if sol.x(lastMaxIndex) < sol.x(lastMinIndex) % Last extremum is a minimum
          lastMinIndex= minIndices(end);
          penultimateMinIndex= minIndices(end-1);
          lastMaxIndex= maxIndices(end);
        else % Last extremum is a maximum
          lastMaxIndex= maxIndices(end);
          penultimateMaxIndex= maxIndices(end-1);
          lastMinIndex= minIndices(end);
        end
```

```
      end
    % Function handles for fminbnd()
     solFn= @(x) deval(sol, x, k);
     minusSolFn= @(x) -deval(sol, x, k);

    % Try to find and characterize a limit cycle oscillation
     if sol.x(lastMaxIndex) < sol.x(lastMinIndex) % Last extremum is a minimum
      % Use last two minima and intervening maximum
      % Use left or right adjacent point depending on slope
       if sol.yp(k,lastMinIndex) < 0
         xLastMin= fminbnd(solFn, sol.x(lastMinIndex), sol.x(lastMinIndex+1));
       elseif sol.yp(k,lastMinIndex) > 0
         xLastMin= fminbnd(solFn, sol.x(lastMinIndex-1), sol.x(lastMinIndex));
       else
         xLastMin= sol.x(lastMinIndex);
       end

       if sol.yp(k,penultimateMinIndex) < 0
         xPenultimateMin= fminbnd(solFn, sol.x(penultimateMinIndex), sol.x(...
penultimateMinIndex+1));
       elseif sol.yp(k,penultimateMinIndex) > 0
         xPenultimateMin= fminbnd(solFn, sol.x(penultimateMinIndex-1), sol.x(...
penultimateMinIndex));
       else
         xPenultimateMin= sol.x(penultimateMinIndex);
       end
      % lastMaxIndex should be inbetween
      % Invert time series so that maxima become minima
       if sol.yp(k,lastMaxIndex) < 0
         xLastMax= fminbnd(minusSolFn, sol.x(lastMaxIndex-1), sol.x(lastMaxIndex));
       elseif sol.yp(k,lastMaxIndex) > 0
         xLastMax= fminbnd(minusSolFn, sol.x(lastMaxIndex), sol.x(lastMaxIndex+1));
       else
         xLastMax= sol.x(lastMaxIndex);
       end

       lastMin= deval(sol, xLastMin, k);
       lastMax= deval(sol, xLastMax, k);
       penultimateMin= deval(sol, xPenultimateMin, k);
       lastAmplitude= lastMax - lastMin;
       penultimateAmplitude= lastMax - penultimateMin;

       if (lastAmplitude ~= 0) && (abs((lastAmplitude - penultimateAmplitude)/...
lastAmplitude) < tol)
         periods(k)= xLastMin - xPenultimateMin;
         medians(k)= (lastMin + lastMax)/2;
```

```
          amplitudes(k)= lastAmplitude;
        else
%           disp(['Omega limit set not found in component ' num2str(k) '; damped ...
oscillations?']);
        end
      else % Last extremum is a maximum
        % Use last two maxima and intervening minimum
        % Use left or right adjacent point depending on slope
        % Time series is inverted to turn maxima into minima
        if sol.yp(k,lastMaxIndex) < 0
          xLastMax= fminbnd(minusSolFn, sol.x(lastMaxIndex-1), sol.x(lastMaxIndex));
        elseif sol.yp(k,lastMaxIndex) > 0
          xLastMax= fminbnd(minusSolFn, sol.x(lastMaxIndex), sol.x(lastMaxIndex+1));
        else
          xLastMax= sol.x(lastMaxIndex);
        end

        if sol.yp(k,penultimateMaxIndex) < 0
          xPenultimateMax= fminbnd(minusSolFn, sol.x(penultimateMaxIndex-1), sol.x(...
penultimateMaxIndex));
        elseif sol.yp(k,penultimateMaxIndex) > 0
          xPenultimateMax= fminbnd(minusSolFn, sol.x(penultimateMaxIndex), sol.x(...
penultimateMaxIndex+1));
        else
          xPenultimateMax= sol.x(penultimateMaxIndex);
        end
        % lastMinIndex should be inbetween
        if sol.yp(k,lastMinIndex) < 0
          xLastMin= fminbnd(solFn, sol.x(lastMinIndex), sol.x(lastMinIndex+1));
        elseif sol.yp(k,lastMinIndex) > 0
          xLastMin= fminbnd(solFn, sol.x(lastMinIndex-1), sol.x(lastMinIndex));
        else
          xLastMin= sol.x(lastMinIndex);
        end

        lastMax= deval(sol, xLastMax, k);
        lastMin= deval(sol, xLastMin, k);
        penultimateMax= deval(sol, xPenultimateMax, k);
        lastAmplitude= lastMax - lastMin;
        penultimateAmplitude= penultimateMax - lastMin;

        if (lastAmplitude ~= 0) && (abs((lastAmplitude - penultimateAmplitude)/...
lastAmplitude) < tol)
          periods(k)= xLastMax - xPenultimateMax;
          medians(k)= (lastMin + lastMax)/2;
          amplitudes(k)= lastAmplitude;
```

```
        else
%           disp( ['Omega limit set not found in component ' num2str(k) '; damped ...
oscillations?'] );
        end
      end
    end
  end
end
```

1.1.8. `sigmoidalGradient.m`. This computes the total protein level using a sigmoidal spatiotemporal gradient.

```
function H13Total= sigmoidalGradient(t, H13TotalMax, H13TotalMin, H13TotalHalfLife)

% Usage:
%  H13Total= sigmoidalGradient(t, H13TotalMax, H13TotalMin, H13TotalHalfLife)
%
% Calculates total Her13.2 gradient at time t.
% 2D Vectorized based on dimensions of t:
%  1) The first (row) dimension represents cell number.
%  2) The second (column) dimension represents time.
%
% H13TotalMax, H13TotalMin, and H13TotalHalfLife may be scalars
%  or column vectors, and are assumed to have the same
%  dimensions. If they are column vectors, then they should have
%  the same number of rows as t.
%
% This function calculates:
%  If t <= 0
%    H13Total= H13TotalMax
%  Elseif 0 < t <= H13TotalHalfLife
%    H13Total= H13TotalMin + (H13TotalMax - H13TotalMin)/2 * (2 - exp(2*(1 - ...
H13TotalHalfLife/t)))
%  Elseif H13TotalHalfLife <= t < 2*H13TotalHalfLife
%    H13Total= H13TotalMin + (H13TotalMax - H13TotalMin)/2 * exp(2*(1 + H13TotalHalfLife...
/(t - 2*H13TotalHalfLife)))
%  Else
%    H13Total= H13TotalMin
%  End

  if isscalar(H13TotalMax)
    H13Total= H13TotalMax*ones(size(t)); % Pre-allocate

    tMask= (0 < t) & (t <= H13TotalHalfLife);
    H13Total(tMask)= H13TotalMin + (H13TotalMax - H13TotalMin)/2*(2 - exp(2*(1 - ...
H13TotalHalfLife./t(tMask))));

    tMask= (H13TotalHalfLife < t) & (t < 2*H13TotalHalfLife);
```

```
    H13Total(tMask)= H13TotalMin + (H13TotalMax - H13TotalMin)/2*exp(2*(1 + ...
H13TotalHalfLife./(t(tMask) - 2*H13TotalHalfLife)));

    tMask= (2*H13TotalHalfLife <= t);
    H13Total(tMask)= H13TotalMin;
  else
    tSize= size(t);
    if tSize(2) > 1 % Match second dimension length
      H13TotalMax= repmat(H13TotalMax, 1, tSize(2));
      H13TotalMin= repmat(H13TotalMin, 1, tSize(2));
      H13TotalHalfLife= repmat(H13TotalHalfLife, 1, tSize(2));
    end
    H13Total= H13TotalMax; % Pre-allocate

    tMask= (0 < t) & (t <= H13TotalHalfLife);
    H13Total(tMask)= H13TotalMin(tMask) + (H13TotalMax(tMask) - H13TotalMin(tMask))/2.*(2...
 - exp(2*(1 - H13TotalHalfLife(tMask)./t(tMask))));

    tMask= (H13TotalHalfLife < t) & (t < 2*H13TotalHalfLife);
    H13Total(tMask)= H13TotalMin(tMask) + (H13TotalMax(tMask) - H13TotalMin(tMask))/2.*...
exp(2*(1 + H13TotalHalfLife(tMask)./(t(tMask) - 2*H13TotalHalfLife(tMask))));

    tMask= (2*H13TotalHalfLife <= t);
    H13Total(tMask)= H13TotalMin(tMask);
  end
end
```

1.1.9. `sigmoidalBeadGradient.m`. This computes the total protein level using a sigmoidal spatiotemporal gradient with a simulated bead. It should be used for simulations of a line of 50 cells.

```
function H13Total= sigmoidalBeadGradient(t, H13TotalMax, H13TotalMin, H13TotalHalfLife)

% Note: This function assumes a 1D line of 50 cells!
%
% Usage:
%  H13Total= sigmoidalBeadGradient(t, H13TotalMax, H13TotalMin, H13TotalHalfLife)
%
% Calculates total Her13.2 gradient at time t.
% 2D Vectorized based on dimensions of t:
%  1) The first (row) dimension represents cell number.
%  2) The second (column) dimension represents time.
%
% H13TotalMax, H13TotalMin, and H13TotalHalfLife may be scalars
%  or column vectors, and are assumed to have the same
%  dimensions. If they are column vectors, then they should have
%  the same number of rows as t.
%
```

```
% This function calculates:
%  If t <= 0 OR the FGF bead is present
%    H13Total= H13TotalMax
%  Elseif 0 < t <= H13TotalHalfLife
%    H13Total= H13TotalMin + (H13TotalMax - H13TotalMin)/2 * (2 - exp(2*(1 - ...
H13TotalHalfLife/t)))
%  Elseif H13TotalHalfLife <= t < 2*H13TotalHalfLife
%    H13Total= H13TotalMin + (H13TotalMax - H13TotalMin)/2 * exp(2*(1 + H13TotalHalfLife...
/(t - 2*H13TotalHalfLife)))
%  Else
%    H13Total= H13TotalMin
%  End

  tSize= size(t);
 % Bead is in the middle of line of cells and a maximum of 10 cells wide
  beadIndices= max(floor(tSize(1)/2 - 5), 0):min(floor(tSize(1)/2 + 5), tSize(1));

  if isscalar(H13TotalMax)
    H13Total= H13TotalMax*ones(tSize); % Pre-allocate

    tMask= (0 < t) & (t <= H13TotalHalfLife);
    tMask(beadIndices,:)= 0; % Simulate FGF bead
    H13Total(tMask)= H13TotalMin + (H13TotalMax - H13TotalMin)/2*(2 - exp(2*(1 - ...
H13TotalHalfLife./t(tMask))));

    tMask= (H13TotalHalfLife < t) & (t < 2*H13TotalHalfLife);
    tMask(beadIndices,:)= 0; % Simulate FGF bead
    H13Total(tMask)= H13TotalMin + (H13TotalMax - H13TotalMin)/2*exp(2*(1 + ...
H13TotalHalfLife./(t(tMask) - 2*H13TotalHalfLife)));

    tMask= (2*H13TotalHalfLife <= t);
    tMask(beadIndices,:)= 0; % Simulate FGF bead
    H13Total(tMask)= H13TotalMin;
  else
    if tSize(2) > 1 % Match second dimension length
      H13TotalMax= repmat(H13TotalMax, 1, tSize(2));
      H13TotalMin= repmat(H13TotalMin, 1, tSize(2));
      H13TotalHalfLife= repmat(H13TotalHalfLife, 1, tSize(2));
    end
    H13Total= H13TotalMax; % Pre-allocate

    tMask= (0 < t) & (t <= H13TotalHalfLife);
    tMask(beadIndices,:)= 0; % Simulate FGF bead
    H13Total(tMask)= H13TotalMin(tMask) + (H13TotalMax(tMask) - H13TotalMin(tMask))/2.*(2...
 - exp(2*(1 - H13TotalHalfLife(tMask)./t(tMask))));
```

```
    tMask= (H13TotalHalfLife < t) & (t < 2*H13TotalHalfLife);
    tMask(beadIndices,:)= 0; % Simulate FGF bead
    H13Total(tMask)= H13TotalMin(tMask) + (H13TotalMax(tMask) - H13TotalMin(tMask))/2.*...
exp(2*(1 + H13TotalHalfLife(tMask)./(t(tMask) - 2*H13TotalHalfLife(tMask))));

    tMask= (2*H13TotalHalfLife <= t);
    tMask(beadIndices,:)= 0; % Simulate FGF bead
    H13Total(tMask)= H13TotalMin(tMask);
  end
end
```

1.1.10. `exponentialGradient.m`. This computes the total protein level using an exponential spatiotemporal gradient.

```
function H13Total= exponentialGradient(t, H13TotalMax, H13TotalMin, H13TotalHalfLife)

% Usage:
%  H13Total= exponentialGradient(t, H13TotalMax, H13TotalMin, H13TotalHalfLife)
%
% Calculates total Her13.2 gradient at time t.
% 2D Vectorized based on dimensions of t:
%  1) The first (row) dimension represents cell number.
%  2) The second (column) dimension represents time.
%
% H13TotalMax, H13TotalMin, and H13TotalHalfLife may be scalars
%  or column vectors, and are assumed to have the same
%  dimensions. If they are column vectors, then they should have
%  the same number of rows as t.
%
% This function calculates:
%  If t <= 0
%    H13Total= H13TotalMax
%  Else
%    H13Total= H13TotalMin + (H13TotalMax - H13TotalMin) * exp(-ln(2)*t/H13TotalHalfLife)
%  End

  if isscalar(H13TotalMax)
    H13Total= H13TotalMax*ones(size(t)); % Pre-allocate

    tMask= (0 < t);
    H13Total(tMask)= H13TotalMin + (H13TotalMax - H13TotalMin)*...
exp(-log(2)*t(tMask)/H13TotalHalfLife);
  else
    tSize= size(t);
    if tSize(2) > 1 % Match second dimension length
      H13TotalMax= repmat(H13TotalMax, 1, tSize(2));
      H13TotalMin= repmat(H13TotalMin, 1, tSize(2));
```

```
        H13TotalHalfLife= repmat(H13TotalHalfLife, 1, tSize(2));
      end
      H13Total= H13TotalMax; % Pre-allocate

      tMask= (0 < t);
      H13Total(tMask)= H13TotalMin(tMask) + ...
(H13TotalMax(tMask) - H13TotalMin(tMask))/2.*...
exp(-log(2)*t(tMask)./H13TotalHalfLife(tMask));
    end
end
```

1.1.11. `zeroCrossingEvent.m`. This short routine, called internally by `ddesd` or `dde23`, ensures that computed solutions are non-negative.

```
function [value, isTerminal, direction]= zeroCrossingEvent(t, z, Z)

 % Stop integration if there is a zero crossing because solutions
 %  should be non-negative. Going negative => something is wrong.

  value= z; % Detect zero crossing in any component
  isTerminal= ones(size(z)); % Stop the integration
  direction= -ones(size(z)); % Negative direction
end
```

1.2. Parameter Estimation Code.

1.2.1. `parameterSamplerParallel.m`. For parameter estimation and model selection purposes, this code runs multiple simulations using `somitogenesis.m` at parameter combinations selected randomly from a parameter space. MATLAB's `parfor` loop structure is used to run simulations with different parameters in parallel on multiple processors, if they are available.

```
function sample= parameterSamplerParallel(argIn1, varargin)

%
% Usage:
%  sample= parameterSamplerParallel(argIn1, varargin)
%
% Compares one and two binding site scenarios for a random sample
%  of select parameters. Linear total protein decay vs.
%  differential decay is chosen with a boolean variable below. A
%  range from an existing parameter sample may be used by passing
%  the parameter sample struct in argIn1 and the first and last
%  sample indices to use in the second and third arguments,
%  respectively. Otherwise, passing a single positive integer in
%  argIn1 generates a new parameter sample of size argIn1.
%  Simulations for different samples can run in parallel by use
%  of the parfor loop.
```

```
parameterSamplerTic= tic;
 % Initialize
  timeStamp= datestr(now,30);
  H13TotalMaxSweepStepSize= 10;
  H13TotalMaxSweepLimit= 2500;
  linearDecayBool= true; % linear OR differential decay
 % Parameters object to broadcast across parfor loops
  paramsBroadcast= Params(false); % false => no parameter noise
  paramsBroadcast.timeInterval= [0 0];
  paramsBroadcast.settlingTime= 250;
  paramsBroadcast.fastModeBool= true;
  paramsBroadcast.gradientFunc= 'constantGradient';

 % Parameter sample ranges (params is a struct)
  params.rhoRange= [1/3 3];
  params.kH13H13LogRange= [1 3];
  params.kH7H7LogRange= [1 3];
  params.kH7H13LogRange= [1 3];
  params.tau_h7Range= [2.3 8.1];
  params.tau_H7Range= paramsBroadcast.tau_H7Mean;
  params.aH7Range= paramsBroadcast.aH7Mean;
  params.bH7Range= [0.20 0.50];
  if linearDecayBool
    params.bH7H7Range= params.bH7Range;
    params.bH7H13Range= params.bH7Range;
  else
    params.bH7H7Range= 0;
    params.bH7H13Range= 0;
  end
  paramsBroadcast.rhoMean= params.rhoRange(1);
  params.gH7Range(2)= paramsBroadcast.gH7Mean;
  paramsBroadcast.rhoMean= params.rhoRange(2);
  params.gH7Range(1)= paramsBroadcast.gH7Mean;
  params.rH7H7LogRange= [-2 0];
  params.wH7H7_H7H7LogRange= [0 2];
  params.dH7Range= paramsBroadcast.dH7Mean;
  params.tau_dRange= paramsBroadcast.tau_dMean;
  params.tau_DRange= paramsBroadcast.tau_DMean;
  params.aDRange= paramsBroadcast.aDMean;
  params.bDRange= paramsBroadcast.bDMean;
  paramsBroadcast.rhoMean= params.rhoRange(1);
  params.gDRange(2)= paramsBroadcast.gDMean;
  paramsBroadcast.rhoMean= params.rhoRange(2);
  params.gDRange(1)= paramsBroadcast.gDMean;
  params.rNLogRange= [-1 1];
  params.wNRange= paramsBroadcast.wNMean;
```

```
  params.wN_H7H7Range= paramsBroadcast.wN_H7H7Mean;
  params.wN_H7H7_H7H7Range= paramsBroadcast.wN_H7H7_H7H7Mean;
  params.dDRange= paramsBroadcast.dDMean;
  params.H13TotalMaxSweep= 0:H13TotalMaxSweepStepSize:H13TotalMaxSweepLimit;
  H13TotalMaxSweepLength= length(params.H13TotalMaxSweep);

  if ~isstruct(argIn1) % Generate a random sample of size argIn1
    sample.sampleSize= argIn1;
    startIndex= 1;
    endIndex= sample.sampleSize;
   % Generate sampling from uniform distributions
    [params.rhoStream, params.kH13H13Stream, params.kH7H7Stream, params.kH7H13Stream, ...
params.tau_h7Stream, params.bH7Stream, params.rH7H7Stream, params.wH7H7_H7H7Stream]= ...
RandStream.create('mrg32k3a', 'NumStreams',8, 'Seed',sum(100*clock));
    rhoSample= params.rhoRange(1) + (params.rhoRange(2) - params.rhoRange(1))*rand(params...
.rhoStream, sample.sampleSize, 1);
    kH13H13Sample= 10.^(params.kH13H13LogRange(1) + (params.kH13H13LogRange(2) - params....
kH13H13LogRange(1))*rand(params.kH13H13Stream, sample.sampleSize,1));
    kH7H7Sample= 10.^(params.kH7H7LogRange(1) + (params.kH7H7LogRange(2) - params....
kH7H7LogRange(1))*rand(params.kH7H7Stream, sample.sampleSize, 1));
    kH7H13Sample= 10.^(params.kH7H13LogRange(1) + (params.kH7H13LogRange(2) - params....
kH7H13LogRange(1))*rand(params.kH13H13Stream, sample.sampleSize, 1));
    tau_h7Sample= params.tau_h7Range(1) + (params.tau_h7Range(2) - params.tau_h7Range(1))...
*rand(params.tau_h7Stream, sample.sampleSize, 1);
    bH7Sample= params.bH7Range(1) + (params.bH7Range(2) - params.bH7Range(1))*rand(params...
.bH7Stream, sample.sampleSize, 1);
    rH7H7Sample= 10.^(params.rH7H7LogRange(1) + (params.rH7H7LogRange(2) - params....
rH7H7LogRange(1))*rand(params.rH7H7Stream, sample.sampleSize, 1));
    wH7H7_H7H7Sample= 10.^(params.wH7H7_H7H7LogRange(1) + (params.wH7H7_H7H7LogRange(2) -...
 params.wH7H7_H7H7LogRange(1))*rand(params.wH7H7_H7H7Stream, sample.sampleSize, 1));
    rNSample= rH7H7Sample/10;
  else % Use given sample
    paramsIn= argIn1;
    if nargin == 3
      startIndex= varargin{1};
      endIndex= varargin{2};
      sample.sampleSize= length(startIndex:endIndex);
    else
      sample.sampleSize= length(paramsIn.rhoSample);
      startIndex= 1;
      endIndex= sample.sampleSize;
    end
   % Generate sampling from existing sample, WIP: Migrate to Streams
    rhoSample= paramsIn.rhoSample(startIndex:endIndex);
    kH13H13Sample= paramsIn.kH13H13Sample(startIndex:endIndex);
    kH7H7Sample= paramsIn.kH7H7Sample(startIndex:endIndex);
```

```
    kH7H13Sample= paramsIn.kH7H13Sample(startIndex:endIndex);
    tau_h7Sample= paramsIn.tau_h7Sample(startIndex:endIndex);
    bH7Sample= paramsIn.bH7Sample(startIndex:endIndex);
    rH7H7Sample= paramsIn.rH7H7Sample(startIndex:endIndex);
    wH7H7_H7H7Sample= paramsIn.wH7H7_H7H7Sample(startIndex:endIndex);
    rNSample= rH7H7Sample/10;
  end

 % Initialize
  oneSiteProblemPoints= [];
  twoSiteProblemPoints= [];
  oneSitePeriods= zeros([paramsBroadcast.numEquations H13TotalMaxSweepLength sample....
sampleSize]);
  oneSiteMedians= oneSitePeriods;
  oneSiteAmplitudes= oneSitePeriods;
  twoSitePeriods= oneSitePeriods;
  twoSiteMedians= oneSitePeriods;
  twoSiteAmplitudes= oneSitePeriods;

 % Run simulation for parameter sample, for both one & two binding sites;
 %  parfor loops can execute in parallel and in any order.
  parfor sampleCtr= 1:sample.sampleSize
%     disp(['Sample #' num2str(sampleCtr)]);
    paramsLocal= paramsBroadcast;
   % Initialize for 1 or 2 binding sites
    paramsLocal.rhoMean= rhoSample(sampleCtr);
    paramsLocal.kH13H13Mean= kH13H13Sample(sampleCtr);
    paramsLocal.kH7H7Mean= kH7H7Sample(sampleCtr);
    paramsLocal.kH7H13Mean= kH7H13Sample(sampleCtr);
    paramsLocal.tau_h7Mean= tau_h7Sample(sampleCtr);
    paramsLocal.bH7Mean= bH7Sample(sampleCtr);
    if linearDecayBool
      paramsLocal.bH7H7Mean= paramsLocal.bH7Mean;
      paramsLocal.bH7H13Mean= paramsLocal.bH7Mean;
    else
      paramsLocal.bH7H7Mean= 0;
      paramsLocal.bH7H13Mean= 0;
    end
    paramsLocal.rH7H7Mean= rH7H7Sample(sampleCtr);
    paramsLocal.wH7H7_H7H7Mean= wH7H7_H7H7Sample(sampleCtr);
    paramsLocal.rNMean= rNSample(sampleCtr);

   % One binding-site
%     disp('One binding-site');
    paramsLocal.numBindingSites= 1;
```

```
    [oneSitePeriods(:,:,sampleCtr), oneSiteMedians(:,:,sampleCtr), oneSiteAmplitudes(:,:,...
sampleCtr), nanFlag]= sweepH13TotalMax(paramsLocal, params.H13TotalMaxSweep);
    if nanFlag
      oneSiteProblemPoints= [oneSiteProblemPoints sampleCtr];
    end

   % Two binding-sites
%     disp('Two binding-sites');
    paramsLocal.numBindingSites= 2;
    [twoSitePeriods(:,:,sampleCtr), twoSiteMedians(:,:,sampleCtr), twoSiteAmplitudes(:,:,...
sampleCtr), nanFlag]= sweepH13TotalMax(paramsLocal, params.H13TotalMaxSweep);
    if nanFlag
      twoSiteProblemPoints= [twoSiteProblemPoints sampleCtr];
    end
  end

  params.rhoSample= rhoSample;
  params.kH13H13Sample= kH13H13Sample;
  params.kH7H7Sample= kH7H7Sample;
  params.kH7H13Sample= kH7H13Sample;
  params.tau_h7Sample= tau_h7Sample;
  params.bH7Sample= bH7Sample;
  params.rH7H7Sample= rH7H7Sample;
  params.wH7H7_H7H7Sample= wH7H7_H7H7Sample;
  params.rNSample= rNSample;
  sample.params= params;

  sample.oneSite.periods=oneSitePeriods;
  sample.oneSite.medians= oneSiteMedians;
  sample.oneSite.amplitudes= oneSiteAmplitudes;
  sample.oneSite.problemPoints= oneSiteProblemPoints;

  sample.twoSite.periods= twoSitePeriods;
  sample.twoSite.medians= twoSiteMedians;
  sample.twoSite.amplitudes= twoSiteAmplitudes;
  sample.twoSite.problemPoints= twoSiteProblemPoints;

toc(parameterSamplerTic)
 % Save sample to a file with a time-stamp
  [success, message, messageID]= mkdir('datasets');
  if success
    if linearDecayBool
      save(['datasets' filesep 'parameterSampleLinear_' timeStamp '_' num2str(startIndex)...
 'to' num2str(endIndex)], 'sample');
    else
```

```
      save(['datasets' filesep 'parameterSampleNonlinear_' timeStamp '_' num2str(...
startIndex) 'to' num2str(endIndex)], 'sample');
    end
  else
    disp('WARNING: Failed to save the dataset to disk.');
  end
 % Warn about problem points
  if ~(isempty(sample.oneSite.problemPoints) && isempty(sample.twoSite.problemPoints))
    disp('WARNING: There are problem points!');
  end
end
```

1.2.2. `sweepH13TotalMax.m`. This function is called by `parameterSamplerParallel.m` to step through a range of total control protein levels and determine the minimum and maximum total control protein levels in that range.

```
function [periods, medians, amplitudes, nanFlag]= ...
  sweepH13TotalMax(params, H13TotalMaxSweep)

 % Initialize & Preallocate
  maxNumLoops= 50;
  H13TotalMaxSweepLength= length(H13TotalMaxSweep);
  periods= zeros(params.numEquations, H13TotalMaxSweepLength);
  medians= periods;
  amplitudes= medians;
  nanFlag= false;

 % Sweep through H13TotalMax values
  for H13TotalMaxCtr= 1:H13TotalMaxSweepLength
    params.H13TotalMaxMean= H13TotalMaxSweep(H13TotalMaxCtr);
%     disp(['H13TotalMaxCtr= ' num2str(H13TotalMaxCtr)]);
    for loopCtr= 1:maxNumLoops
%       disp(['loopCtr= ' num2str(loopCtr)]);
      sol= somitogenesis(params);
      periods(:,H13TotalMaxCtr)= sol.statistics.periods;
      medians(:,H13TotalMaxCtr)= sol.statistics.medians;
      amplitudes(:,H13TotalMaxCtr)= sol.statistics.amplitudes;
     % Create new history function for next iteration, note t-shift
      params.history= @(t) deval(sol, t + sol.params.settlingTime);

      if ~any(isnan(periods(:,H13TotalMaxCtr)))
        break;
      elseif loopCtr == maxNumLoops
        nanFlag= true;
      end
    end

    if nanFlag
```

```
      break;
    end
  end
end
```

1.2.3. `parameterStatistics.m`. This code statistically analyzes parameter sampling results for model scenarios I–IV using outputs from `parameterSamplerParallel.m`. The results of these analyses enable model selection.

```
function output= parameterStatistics(nonlinearSample, linearSample)

% Usage:
%  parameterStatistics(nonlinearSample, linearSample)
%
% This function does a comparative statistical analysis on model
%  scenarios I-IV. Each of the two input datasets,
%  nonlinearSample and linearSample, includes one and two
%  binding site data. nonlinearSample and linearSample are
%  required to have the same dimension datasets.

  disp(' ');

  if ~isempty(nonlinearSample.oneSite.problemPoints) || ~isempty(nonlinearSample.twoSite....
problemPoints) || ~isempty(linearSample.oneSite.problemPoints) || ~isempty(linearSample....
twoSite.problemPoints)
    disp('WARNING: There are problem points!');
    disp(' ');
  end

  amplitudeThresh= 5; % Require at least this peak-to-peak amplitude of mRNA & protein ...
molecules
  periodTol= 0.01; % Require that mRNA & protein oscillation periods agree to within this
  targetPeriod= 30; % Desired period of oscillation
  deltaPeriod= 3; % Allowable +/- range in oscillation period
  targetDelta= 15; % Filter out changes in period less than this

  [numEquations numSweeps numSamples]= size(nonlinearSample.oneSite.amplitudes);

% One binding site, nonlinear decay
  disp('MODEL SCENARIO I, 1 binding site, nonlinear decay:');
 % Period mask
  periodMeans1n= mean(nonlinearSample.oneSite.periods, 1);
  fullPeriodMeans1n = repmat(periodMeans1n, [numEquations, 1, 1]);
  periodMeans1n= squeeze(periodMeans1n)';
  periodMask1n= zeros(size(fullPeriodMeans1n));
  periodMask1n(fullPeriodMeans1n ~= 0)= abs((nonlinearSample.oneSite.periods(...
fullPeriodMeans1n ~= 0) - fullPeriodMeans1n(fullPeriodMeans1n ~= 0))./fullPeriodMeans1n(...
fullPeriodMeans1n ~= 0)) < periodTol;
```

```
  periodMask1n(fullPeriodMeans1n == 0)= abs(nonlinearSample.oneSite.periods(...
fullPeriodMeans1n == 0)) < periodTol;
  periodMask1n= squeeze(all(periodMask1n, 1))';
  clear fullPeriodMeans1n
 % Amplitude mask
  amplitudeMask1n= squeeze(all(amplitudeThresh <=nonlinearSample.oneSite.amplitudes, 1))...
';
 % Combine masks and find periodic proportion
  fullOscMask1n= periodMask1n & amplitudeMask1n;
  oscMask1n= any(fullOscMask1n, 2);
  oscIndices1n= find(oscMask1n);
  oscProp1n= length(oscIndices1n)/numSamples;
  disp(['Periodic proportion: ' num2str(length(oscIndices1n)) '/' num2str(numSamples) '...
= ' num2str(oscProp1n) ]);

  [maxArray1n, maxIndices1n]= max(fullOscMask1n.*periodMeans1n, [], 2);
  globalMax1n= max(maxArray1n);
  minArray1n= zeros(numSamples, 1);
  minIndices1n= ones(numSamples, 1);
  for k= 1:numSamples
   % Finding minimum strictly positive period
    positiveIndices= fullOscMask1n(k,:) > 0;
    if any(positiveIndices)
      [minArray1n(k), minIndices1n(k)]= min(periodMeans1n(k, positiveIndices));
    end
  end
  globalMin1n= min(minArray1n(0 < minArray1n));
  if isempty(globalMin1n)
    disp('No period range.');
  else
    disp(['Nontrivial period range: [' num2str(globalMin1n) ', ' num2str(globalMax1n) '...
]' ]);
  end

 % Compute min and max H13Total parameters
  oscH13TotalSweepIndicesMin1n= minIndices1n(0 < minArray1n);
  oscH13TotalSweepIndicesMax1n= maxIndices1n(0 < maxArray1n);
  oscH13TotalMinValues1n= nan(min(length(oscH13TotalSweepIndicesMin1n), ...
length(oscH13TotalSweepIndicesMax1n)),1);
  oscH13TotalMaxValues1n= oscH13TotalMinValues1n;
  if (length(oscH13TotalSweepIndicesMin1n) ~= length(oscH13TotalSweepIndicesMax1n))
    disp('WARNING: Different length min/max vectors for total control protein.');
  else
    for k= 1:length(oscH13TotalSweepIndicesMin1n)
     % Note that the ordering of the max and min period is not guaranteed
      oscH13TotalMinValues1n(k)= min(...
```

```
nonlinearSample.params.H13TotalMaxSweep(oscH13TotalSweepIndicesMin1n(k)), ...
nonlinearSample.params.H13TotalMaxSweep(oscH13TotalSweepIndicesMax1n(k)));
      oscH13TotalMaxValues1n(k)= max(...
nonlinearSample.params.H13TotalMaxSweep(oscH13TotalSweepIndicesMin1n(k)), ...
nonlinearSample.params.H13TotalMaxSweep(oscH13TotalSweepIndicesMax1n(k)));
    end
  end

  [deltaMax1n, deltaMaxIndex1n]= max(maxArray1n - minArray1n);
  if ~isempty(oscIndices1n)
    disp([ 'Maximum Delta T: [' num2str(minArray1n(deltaMaxIndex1n)) ', ' num2str(...
maxArray1n(deltaMaxIndex1n)) '], Delta T= ' num2str(deltaMax1n) ', k= ' num2str(...
deltaMaxIndex1n) ', j= [' num2str(minIndices1n(deltaMaxIndex1n)) ',' num2str(maxIndices1n...
(deltaMaxIndex1n)) ']' ]);
  else
    disp('Maximum Delta T: NaN');
  end

 % Analyze subset of data with tailbud period in desired range
 %  Uses IVT which assumes period is a cts. fn. of H13TotalMax
  periodOscMask1n= (minArray1n <= targetPeriod + deltaPeriod) & (targetPeriod - ...
deltaPeriod <= maxArray1n);
  periodOscIndices1n= find(periodOscMask1n);
  periodOscProp1n= length(periodOscIndices1n)/numSamples;
  disp([ num2str(targetPeriod) ' +/- ' num2str(deltaPeriod) ' min period proportion: ' ...
num2str(length(periodOscIndices1n)) '/' num2str(numSamples) '= ' num2str(periodOscProp1n)...
 ]);

  periodMaxArray1n= periodOscMask1n.*maxArray1n;
  periodMaxIndices1n= maxIndices1n;
  periodMaxIndices1n(periodOscMask1n == 0)= 1;
  periodMinArray1n= periodOscMask1n.*minArray1n;
  periodMinIndices1n= minIndices1n;
  periodMinIndices1n(periodOscMask1n == 0)= 1;
  periodDeltaArray1n= periodMaxArray1n - periodMinArray1n;
  [periodDeltaMax1n, periodDeltaMaxIndex1n]= max(periodDeltaArray1n);
  if ~isempty(periodOscIndices1n)
```

```
    disp([ 'Maximum␣Delta␣T␣containing␣' num2str(targetPeriod) '␣+/-␣' num2str(...
deltaPeriod) '␣:␣[' num2str(periodMinArray1n(periodDeltaMaxIndex1n)) ',␣' num2str(...
periodMaxArray1n(periodDeltaMaxIndex1n)) '],␣Delta␣T=␣' num2str(periodDeltaMax1n) ',␣k=␣...
' num2str(periodDeltaMaxIndex1n) ',␣j=␣[' num2str(periodMinIndices1n(...
periodDeltaMaxIndex1n)) ',' num2str(periodMaxIndices1n(periodDeltaMaxIndex1n)) ']␣@' 10 ...
'␣rho=␣' num2str(nonlinearSample.params.rhoSample(periodDeltaMaxIndex1n)) ',␣kH13H13=␣' ...
num2str(nonlinearSample.params.kH13H13Sample(periodDeltaMaxIndex1n)) ',␣kH7H7=␣' num2str(...
nonlinearSample.params.kH7H7Sample(periodDeltaMaxIndex1n)) ',␣kH7H13=␣' num2str(...
nonlinearSample.params.kH7H13Sample(periodDeltaMaxIndex1n)) ',␣tau_h7=␣' num2str(...
nonlinearSample.params.tau_h7Sample(periodDeltaMaxIndex1n)) ',␣bH7=␣' num2str(...
nonlinearSample.params.bH7Sample(periodDeltaMaxIndex1n)) ',␣rH7H7=␣' num2str(...
nonlinearSample.params.rH7H7Sample(periodDeltaMaxIndex1n)) ',␣wH7H7_H7H7=␣' num2str(...
nonlinearSample.params.wH7H7_H7H7Sample(periodDeltaMaxIndex1n)) ',␣rN=␣' num2str(...
nonlinearSample.params.rNSample(periodDeltaMaxIndex1n))]);
  else
    disp([ 'Maximum␣Delta␣T␣containing␣' num2str(targetPeriod) '␣+/-␣' num2str(...
deltaPeriod) '␣:␣NaN' ]);
  end

  periodOscH13TotalSweepIndicesMin1n= periodMinIndices1n(0 < periodMinArray1n);
  periodOscH13TotalSweepIndicesMax1n= periodMaxIndices1n(0 < periodMaxArray1n);
  periodOscH13TotalMinValues1n= nan(min(length(periodOscH13TotalSweepIndicesMin1n), ...
length(periodOscH13TotalSweepIndicesMax1n)),1);
  periodOscH13TotalMaxValues1n= periodOscH13TotalMinValues1n;
  if (length(periodOscH13TotalSweepIndicesMin1n) ~= length(...
periodOscH13TotalSweepIndicesMax1n))
    disp('WARNING:␣Different␣length␣min/max␣vectors␣for␣total␣control␣protein.');
  else
    for k= 1:length(periodOscH13TotalSweepIndicesMin1n)
     % Note that the ordering of the max and min period is not guaranteed
      periodOscH13TotalMinValues1n(k)= min(...
nonlinearSample.params.H13TotalMaxSweep(periodOscH13TotalSweepIndicesMin1n(k)), ...
nonlinearSample.params.H13TotalMaxSweep(periodOscH13TotalSweepIndicesMax1n(k)));
      periodOscH13TotalMaxValues1n(k)= max(...
nonlinearSample.params.H13TotalMaxSweep(periodOscH13TotalSweepIndicesMin1n(k)), ...
nonlinearSample.params.H13TotalMaxSweep(periodOscH13TotalSweepIndicesMax1n(k)));
    end
  end

  periodDeltaCountFuncH1n= @(deltaTMin) nnz(deltaTMin <= periodDeltaArray1n(...
periodOscIndices1n));
  output.nonlinearDecay.oneSiteIndices= periodOscIndices1n(targetDelta <= ...
periodDeltaArray1n(periodOscIndices1n))';
```

```
  disp([ num2str(targetPeriod) '␣+/-␣' num2str(deltaPeriod) '␣min␣period␣proportion␣with...
␣Delta␣T␣>=␣' num2str(targetDelta) ':␣' num2str(periodDeltaCountFuncH1n(targetDelta)) '/...
' num2str(numSamples) '=␣' num2str(periodDeltaCountFuncH1n(targetDelta)/numSamples) ]);

  disp('␣');

% One binding site, linear decay
  disp('MODEL␣SCENARIO␣II,␣1␣binding␣site,␣linear␣decay:');
 % Period mask
  periodMeans1l= mean(linearSample.oneSite.periods, 1);
  fullPeriodMeans1l= repmat(periodMeans1l, [numEquations, 1, 1]);
  periodMeans1l= squeeze(periodMeans1l)';
  periodMask1l= zeros(size(fullPeriodMeans1l));
  periodMask1l(fullPeriodMeans1l ~= 0)= abs((linearSample.oneSite.periods(...
fullPeriodMeans1l ~= 0) - fullPeriodMeans1l(fullPeriodMeans1l ~= 0))./fullPeriodMeans1l(...
fullPeriodMeans1l ~= 0)) < periodTol;
  periodMask1l(fullPeriodMeans1l == 0)= abs(linearSample.oneSite.periods(...
fullPeriodMeans1l == 0)) < periodTol;
  periodMask1l= squeeze(all(periodMask1l, 1))';
  clear fullPeriodMeans1l
 % Amplitude mask
  amplitudeMask1l= squeeze(all(amplitudeThresh <= linearSample.oneSite.amplitudes, 1))';
 % Combine masks and find periodic proportion
  fullOscMask1l= periodMask1l & amplitudeMask1l;
  oscMask1l= any(fullOscMask1l, 2);
  oscIndices1l= find(oscMask1l);
  oscProp1l= length(oscIndices1l)/numSamples;
  disp([ 'Periodic␣proportion:␣'  num2str(length(oscIndices1l)) '/' num2str(numSamples) ...
'=␣' num2str(oscProp1l) ]);

  [maxArray1l, maxIndices1l]= max(fullOscMask1l.*periodMeans1l, [], 2);
  globalMax1l= max(maxArray1l);
  minArray1l= zeros(numSamples, 1);
  minIndices1l= ones(numSamples, 1);
  for k= 1:numSamples
    positiveIndices= fullOscMask1l(k,:) > 0;
    if any(positiveIndices)
      [minArray1l(k), minIndices1l(k)]= min(periodMeans1l(k, positiveIndices));
    end
  end
  globalMin1l= min(minArray1l(0 < minArray1l));
  if isempty(globalMin1l)
    disp('No␣period␣range.');
  else
    disp([ 'Nontrivial␣period␣range:␣[' num2str(globalMin1l) ',␣' num2str(globalMax1l) '...
]' ]);
```

```
  end

  oscH13TotalSweepIndicesMin1l= minIndices1l(0 < minArray1l);
  oscH13TotalSweepIndicesMax1l= maxIndices1l(0 < maxArray1l);
  oscH13TotalMinValues1l= nan(min(length(oscH13TotalSweepIndicesMin1l), ...
length(oscH13TotalSweepIndicesMax1l)),1);
  oscH13TotalMaxValues1l= oscH13TotalMinValues1l;
  if (length(oscH13TotalSweepIndicesMin1l) ~= length(oscH13TotalSweepIndicesMax1l))
    disp('WARNING: Different length min/max vectors for total control protein.');
  else
    for k= 1:length(oscH13TotalSweepIndicesMin1l)
     % Note that the ordering of the max and min period is not guaranteed
      oscH13TotalMinValues1l(k)= min(...
nonlinearSample.params.H13TotalMaxSweep(oscH13TotalSweepIndicesMin1l(k)), ...
nonlinearSample.params.H13TotalMaxSweep(oscH13TotalSweepIndicesMax1l(k)));
      oscH13TotalMaxValues1l(k)= max(...
nonlinearSample.params.H13TotalMaxSweep(oscH13TotalSweepIndicesMin1l(k)), ...
nonlinearSample.params.H13TotalMaxSweep(oscH13TotalSweepIndicesMax1l(k)));
    end
  end

  [deltaMax1l, deltaMaxIndex1l]= max(maxArray1l - minArray1l);
  if ~isempty(oscIndices1l)
    disp([ 'Maximum Delta T: [' num2str(minArray1l(deltaMaxIndex1l)) ', ' num2str(...
maxArray1l(deltaMaxIndex1l)) '], Delta T= ' num2str(deltaMax1l) ', k= ' num2str(...
deltaMaxIndex1l) ', j= [' num2str(minIndices1l(deltaMaxIndex1l)) ',' num2str(maxIndices1l...
(deltaMaxIndex1l)) ']' ]);
  else
    disp('Maximum Delta T: NaN');
  end

  periodOscMask1l= (minArray1l <= targetPeriod + deltaPeriod) & (targetPeriod - ...
deltaPeriod <= maxArray1l);
  periodOscIndices1l= find(periodOscMask1l);
  periodOscProp1l= length(periodOscIndices1l)/numSamples;
  disp([ num2str(targetPeriod) ' +/- ' num2str(deltaPeriod) ' min period proportion: ' ...
num2str(length(periodOscIndices1l)) '/' num2str(numSamples) '= ' num2str(periodOscProp1l)...
 ]);

  periodMaxArray1l= periodOscMask1l.*maxArray1l;
  periodMaxIndices1l= maxIndices1l;
  periodMaxIndices1l(periodOscMask1l == 0)= 1;
  periodMinArray1l= periodOscMask1l.*minArray1l;
  periodMinIndices1l= minIndices1l;
  periodMinIndices1l(periodOscMask1l == 0)= 1;
  periodDeltaArray1l= periodMaxArray1l - periodMinArray1l;
```

```
  [periodDeltaMax11, periodDeltaMaxIndex11]= max(periodDeltaArray11);
  if ~isempty(periodOscIndices11)
    disp([ 'Maximum Delta T containing ' num2str(targetPeriod) ' +/- ' num2str(...
deltaPeriod) ' : [' num2str(periodMinArray11(periodDeltaMaxIndex11)) ', ' num2str(...
periodMaxArray11(periodDeltaMaxIndex11)) '], Delta T= ' num2str(periodDeltaMax11) ', k= ...
' num2str(periodDeltaMaxIndex11) ', j= [' num2str(periodMinIndices11(...
periodDeltaMaxIndex11)) ',' num2str(periodMaxIndices11(periodDeltaMaxIndex11)) '] @' 10 ...
' rho= ' num2str(linearSample.params.rhoSample(periodDeltaMaxIndex11)) ', kH13H13= ' ...
num2str(linearSample.params.kH13H13Sample(periodDeltaMaxIndex11)) ', kH7H7= ' num2str(...
linearSample.params.kH7H7Sample(periodDeltaMaxIndex11)) ', kH7H13= ' num2str(linearSample...
.params.kH7H13Sample(periodDeltaMaxIndex11)) ', tau_h7= ' num2str(linearSample.params....
tau_h7Sample(periodDeltaMaxIndex11)) ', bH7= ' num2str(linearSample.params.bH7Sample(...
periodDeltaMaxIndex11)) ', rH7H7= ' num2str(linearSample.params.rH7H7Sample(...
periodDeltaMaxIndex11)) ', wH7H7_H7H7= ' num2str(linearSample.params.wH7H7_H7H7Sample(...
periodDeltaMaxIndex11)) ', rN= ' num2str(linearSample.params.rNSample(...
periodDeltaMaxIndex11))]);
  else
    disp([ 'Maximum Delta T containing ' num2str(targetPeriod) ' +/- ' num2str(...
deltaPeriod) ' : NaN' ]);
  end

  periodOscH13TotalSweepIndicesMin11= periodMinIndices11(0 < periodMinArray11);
  periodOscH13TotalSweepIndicesMax11= periodMaxIndices11(0 < periodMaxArray11);
  periodOscH13TotalMinValues11= nan(min(length(periodOscH13TotalSweepIndicesMin11), ...
length(periodOscH13TotalSweepIndicesMax11)),1);
  periodOscH13TotalMaxValues11= periodOscH13TotalMinValues11;
  if (length(periodOscH13TotalSweepIndicesMin11) ~= length(...
periodOscH13TotalSweepIndicesMax11))
    disp('WARNING: Different length min/max vectors for total control protein.');
  else
    for k= 1:length(periodOscH13TotalSweepIndicesMin11)
     % Note that the ordering of the max and min period is not guaranteed
      periodOscH13TotalMinValues11(k)= min(...
nonlinearSample.params.H13TotalMaxSweep(periodOscH13TotalSweepIndicesMin11(k)), ...
nonlinearSample.params.H13TotalMaxSweep(periodOscH13TotalSweepIndicesMax11(k)));
      periodOscH13TotalMaxValues11(k)= max(...
nonlinearSample.params.H13TotalMaxSweep(periodOscH13TotalSweepIndicesMin11(k)), ...
nonlinearSample.params.H13TotalMaxSweep(periodOscH13TotalSweepIndicesMax11(k)));
    end
  end

  periodDeltaCountFuncH11= @(deltaTMin) nnz(deltaTMin <= periodDeltaArray11(...
periodOscIndices11));
  output.linearDecay.oneSiteIndices= periodOscIndices11(targetDelta <= periodDeltaArray11...
(periodOscIndices11))';
```

```
  disp([ num2str(targetPeriod) ' +/- ' num2str(deltaPeriod) ' min period proportion with...
 Delta T >= ' num2str(targetDelta) ': ' num2str(periodDeltaCountFuncH1l(targetDelta)) '/...
' num2str(numSamples) '= ' num2str(periodDeltaCountFuncH1l(targetDelta)/numSamples) ]);

  disp(' ');

% Two binding sites, nonlinear decay
  disp('MODEL SCENARIO III, 2 binding sites, nonlinear decay:');
 % Period mask
  periodMeans2n= mean(nonlinearSample.twoSite.periods, 1);
  fullPeriodMeans2n = repmat(periodMeans2n, [numEquations, 1, 1]);
  periodMeans2n= squeeze(periodMeans2n)';
  periodMask2n= zeros(size(fullPeriodMeans2n));
  periodMask2n(fullPeriodMeans2n ~= 0)= abs((nonlinearSample.twoSite.periods(...
fullPeriodMeans2n ~= 0) - fullPeriodMeans2n(fullPeriodMeans2n ~= 0))./fullPeriodMeans2n(...
fullPeriodMeans2n ~= 0)) < periodTol;
  periodMask2n(fullPeriodMeans2n == 0)= abs(nonlinearSample.twoSite.periods(...
fullPeriodMeans2n == 0)) < periodTol;
  periodMask2n= squeeze(all(periodMask2n, 1))';
  clear fullPeriodMeans2n;
 % Amplitude mask
  amplitudeMask2n= squeeze(all(amplitudeThresh <= nonlinearSample.twoSite.amplitudes, 1))...
';
 % Combine masks and find periodic proportion
  fullOscMask2n= periodMask2n & amplitudeMask2n;
  oscMask2n= any(fullOscMask2n, 2);
  oscIndices2n= find(oscMask2n);
  oscProp2n= length(oscIndices2n)/numSamples;
  disp([ 'Periodic proportion: '  num2str(length(oscIndices2n)) '/' num2str(numSamples) ...
'= ' num2str(oscProp2n) ]);

  [maxArray2n, maxIndices2n]= max(fullOscMask2n.*periodMeans2n, [], 2);
  globalMax2n= max(maxArray2n);
  minArray2n= zeros(numSamples, 1);
  minIndices2n= ones(numSamples, 1);
  for k= 1:numSamples
    positiveIndices= fullOscMask2n(k,:) > 0;
    if any(positiveIndices)
      [minArray2n(k), minIndices2n(k)]= min(periodMeans2n(k, positiveIndices));
    end
  end
  globalMin2n= min(minArray2n(0 < minArray2n));
  if isempty(globalMin2n)
    disp('No period range.');
  else
```

```
    disp([ 'Nontrivial␣period␣range:␣[' num2str(globalMin2n) ',␣' num2str(globalMax2n) '...
]' ]);
  end

  oscH13TotalSweepIndicesMin2n= minIndices2n(0 < minArray2n);
  oscH13TotalSweepIndicesMax2n= maxIndices2n(0 < maxArray2n);
  oscH13TotalMinValues2n= nan(min(length(oscH13TotalSweepIndicesMin2n), ...
length(oscH13TotalSweepIndicesMax2n)),1);
  oscH13TotalMaxValues2n= oscH13TotalMinValues2n;
  if (length(oscH13TotalSweepIndicesMin2n) ~= length(oscH13TotalSweepIndicesMax2n))
    disp('WARNING:␣Different␣length␣min/max␣vectors␣for␣total␣control␣protein.');
  else
    for k= 1:length(oscH13TotalSweepIndicesMin2n)
     % Note that the ordering of the max and min period is not guaranteed
      oscH13TotalMinValues2n(k)= min(...
nonlinearSample.params.H13TotalMaxSweep(oscH13TotalSweepIndicesMin2n(k)), ...
nonlinearSample.params.H13TotalMaxSweep(oscH13TotalSweepIndicesMax2n(k)));
      oscH13TotalMaxValues2n(k)= max(...
nonlinearSample.params.H13TotalMaxSweep(oscH13TotalSweepIndicesMin2n(k)), ...
nonlinearSample.params.H13TotalMaxSweep(oscH13TotalSweepIndicesMax2n(k)));
    end
  end

  [deltaMax2n, deltaMaxIndex2n]= max(maxArray2n - minArray2n);
  if ~isempty(oscIndices2n)
    disp([ 'Maximum␣Delta␣T:␣[' num2str(minArray2n(deltaMaxIndex2n)) ',␣' num2str(...
maxArray2n(deltaMaxIndex2n)) '],␣Delta␣T=␣' num2str(deltaMax2n) ',␣k=␣' num2str(...
deltaMaxIndex2n) ',␣j=␣[' num2str(minIndices2n(deltaMaxIndex2n)) ',␣' num2str(...
maxIndices2n(deltaMaxIndex2n)) ']' ]);
  else
    disp('Maximum␣Delta␣T:␣NaN');
  end

  periodOscMask2n= (minArray2n <= targetPeriod + deltaPeriod) & (targetPeriod - ...
deltaPeriod <= maxArray2n);
  periodOscIndices2n= find(periodOscMask2n);
  periodOscProp2n= length(periodOscIndices2n)/numSamples;
  disp([ num2str(targetPeriod) '␣+/-␣' num2str(deltaPeriod) '␣min␣period␣proportion:␣' ...
num2str(length(periodOscIndices2n)) '/' num2str(numSamples) '=␣' num2str(periodOscProp2n)...
 ]);

  periodMaxArray2n= periodOscMask2n.*maxArray2n;
  periodMaxIndices2n= maxIndices2n;
  periodMaxIndices2n(periodOscMask2n == 0)= 1;
  periodMinArray2n= periodOscMask2n.*minArray2n;
  periodMinIndices2n= minIndices2n;
```

```
  periodMinIndices2n(periodOscMask2n == 0)= 1;
  periodDeltaArray2n= periodMaxArray2n - periodMinArray2n;
  [periodDeltaMax2n, periodDeltaMaxIndex2n]= max(periodDeltaArray2n);
  if ~isempty(periodOscIndices2n)
    disp(['Maximum Delta T containing ' num2str(targetPeriod) ' +/- ' num2str(...
deltaPeriod) ' : [' num2str(periodMinArray2n(periodDeltaMaxIndex2n)) ', ' num2str(...
periodMaxArray2n(periodDeltaMaxIndex2n)) '], Delta T= ' num2str(periodDeltaMax2n) ', k= ...
' num2str(periodDeltaMaxIndex2n) ', j= [' num2str(periodMinIndices2n(...
periodDeltaMaxIndex2n)) ',' num2str(periodMaxIndices2n(periodDeltaMaxIndex2n)) '] @' 10 ...
' rho= ' num2str(nonlinearSample.params.rhoSample(periodDeltaMaxIndex2n)) ', kH13H13= ' ...
num2str(nonlinearSample.params.kH13H13Sample(periodDeltaMaxIndex2n)) ', kH7H7= ' num2str(...
nonlinearSample.params.kH7H7Sample(periodDeltaMaxIndex2n)) ', kH7H13= ' num2str(...
nonlinearSample.params.kH7H13Sample(periodDeltaMaxIndex2n)) ', tau_h7= ' num2str(...
nonlinearSample.params.tau_h7Sample(periodDeltaMaxIndex2n)) ', bH7= ' num2str(...
nonlinearSample.params.bH7Sample(periodDeltaMaxIndex2n)) ', rH7H7= ' num2str(...
nonlinearSample.params.rH7H7Sample(periodDeltaMaxIndex2n)) ', wH7H7_H7H7= ' num2str(...
nonlinearSample.params.wH7H7_H7H7Sample(periodDeltaMaxIndex2n)) ', rN= ' num2str(...
nonlinearSample.params.rNSample(periodDeltaMaxIndex2n))]);
  else
    disp(['Maximum Delta T containing ' num2str(targetPeriod) ' +/- ' num2str(...
deltaPeriod) ' : NaN' ]);
  end

  periodOscH13TotalSweepIndicesMin2n= periodMinIndices2n(0 < periodMinArray2n);
  periodOscH13TotalSweepIndicesMax2n= periodMaxIndices2n(0 < periodMaxArray2n);
  periodOscH13TotalMinValues2n= nan(min(length(periodOscH13TotalSweepIndicesMin2n), ...
length(periodOscH13TotalSweepIndicesMax2n)),1);
  periodOscH13TotalMaxValues2n= periodOscH13TotalMinValues2n;
  if (length(periodOscH13TotalSweepIndicesMin2n) ~= length(...
periodOscH13TotalSweepIndicesMax2n))
    disp('WARNING: Different length min/max vectors for total control protein.');
  else
    for k= 1:length(periodOscH13TotalSweepIndicesMin2n)
     % Note that the ordering of the max and min period is not guaranteed
      periodOscH13TotalMinValues2n(k)= min(...
nonlinearSample.params.H13TotalMaxSweep(periodOscH13TotalSweepIndicesMin2n(k)), ...
nonlinearSample.params.H13TotalMaxSweep(periodOscH13TotalSweepIndicesMax2n(k)));
      periodOscH13TotalMaxValues2n(k)= max(...
nonlinearSample.params.H13TotalMaxSweep(periodOscH13TotalSweepIndicesMin2n(k)), ...
nonlinearSample.params.H13TotalMaxSweep(periodOscH13TotalSweepIndicesMax2n(k)));
    end
  end

  halfTargetDeltaIndices2n= ...
periodOscIndices2n(targetDelta/2 <= periodDeltaArray2n(periodOscIndices2n));
  halfTargetDeltaH13TotalSweepIndicesMin2n= periodMinIndices2n(halfTargetDeltaIndices2n);
```

```
  halfTargetDeltaH13TotalSweepIndicesMax2n= periodMaxIndices2n(halfTargetDeltaIndices2n);
  halfTargetDeltaH13TotalMinValues2n= nan(min(length(...
halfTargetDeltaH13TotalSweepIndicesMin2n), ...
length(halfTargetDeltaH13TotalSweepIndicesMax2n)),1);
  halfTargetDeltaH13TotalMaxValues2n= halfTargetDeltaH13TotalMinValues2n;
  if (length(halfTargetDeltaH13TotalSweepIndicesMin2n) ~= length(...
halfTargetDeltaH13TotalSweepIndicesMax2n))
    disp('WARNING: Different length min/max vectors for total control protein.');
  else
    for k= 1:length(halfTargetDeltaH13TotalSweepIndicesMin2n)
     % Note that the ordering of the max and min period is not guaranteed
      halfTargetDeltaH13TotalMinValues2n(k)= min(...
nonlinearSample.params.H13TotalMaxSweep(halfTargetDeltaH13TotalSweepIndicesMin2n(k)), ...
nonlinearSample.params.H13TotalMaxSweep(halfTargetDeltaH13TotalSweepIndicesMax2n(k)));
      halfTargetDeltaH13TotalMaxValues2n(k)= max(...
nonlinearSample.params.H13TotalMaxSweep(halfTargetDeltaH13TotalSweepIndicesMin2n(k)), ...
nonlinearSample.params.H13TotalMaxSweep(halfTargetDeltaH13TotalSweepIndicesMax2n(k)));
    end
  end

  targetDeltaIndices2n= ...
periodOscIndices2n(targetDelta <= periodDeltaArray2n(periodOscIndices2n));
  targetDeltaH13TotalSweepIndicesMin2n= periodMinIndices2n(targetDeltaIndices2n);
  targetDeltaH13TotalSweepIndicesMax2n= periodMaxIndices2n(targetDeltaIndices2n);
  targetDeltaH13TotalMinValues2n= nan(min(length(targetDeltaH13TotalSweepIndicesMin2n), ...
...
length(targetDeltaH13TotalSweepIndicesMax2n)),1);
  targetDeltaH13TotalMaxValues2n= targetDeltaH13TotalMinValues2n;
  if (length(targetDeltaH13TotalSweepIndicesMin2n) ~= length(...
targetDeltaH13TotalSweepIndicesMax2n))
    disp('WARNING: Different length min/max vectors for total control protein.');
  else
    for k= 1:length(targetDeltaH13TotalSweepIndicesMin2n)
     % Note that the ordering of the max and min period is not guaranteed
      targetDeltaH13TotalMinValues2n(k)= min(...
nonlinearSample.params.H13TotalMaxSweep(targetDeltaH13TotalSweepIndicesMin2n(k)), ...
nonlinearSample.params.H13TotalMaxSweep(targetDeltaH13TotalSweepIndicesMax2n(k)));
      targetDeltaH13TotalMaxValues2n(k)= max(...
nonlinearSample.params.H13TotalMaxSweep(targetDeltaH13TotalSweepIndicesMin2n(k)), ...
nonlinearSample.params.H13TotalMaxSweep(targetDeltaH13TotalSweepIndicesMax2n(k)));
    end
  end

  periodDeltaCountFuncH2n= @(deltaTMin) nnz(deltaTMin <= periodDeltaArray2n(...
periodOscIndices2n));
```

```
  output.nonlinearDecay.twoSiteIndices= periodOscIndices2n(targetDelta <= ...
periodDeltaArray2n(periodOscIndices2n))';
  output.nonlinearDecay.twoSiteH13TotalAtMinPeriod= nonlinearSample.params....
H13TotalMaxSweep(periodMinIndices2n(output.nonlinearDecay.twoSiteIndices));

  disp([num2str(targetPeriod) ' +/- ' num2str(deltaPeriod) ' min period proportion with...
 Delta T >= ' num2str(targetDelta) ': ' num2str(periodDeltaCountFuncH2n(targetDelta)) '/...
' num2str(numSamples) ' = ' num2str(periodDeltaCountFuncH2n(targetDelta)/numSamples) ]);

  disp(' ');

% Two binding sites, linear decay
  disp('MODEL SCENARIO IV, 2 binding sites, linear decay:');
 % Period mask
  periodMeans2l= mean(linearSample.twoSite.periods, 1);
  fullPeriodMeans2l = repmat(periodMeans2l, [numEquations, 1, 1]);
  periodMeans2l= squeeze(periodMeans2l)';
  periodMask2l= zeros(size(fullPeriodMeans2l));
  periodMask2l(fullPeriodMeans2l ~= 0)= abs((linearSample.twoSite.periods(...
fullPeriodMeans2l ~= 0) - fullPeriodMeans2l(fullPeriodMeans2l ~= 0))./fullPeriodMeans2l(...
fullPeriodMeans2l ~= 0)) < periodTol;
  periodMask2l(fullPeriodMeans2l == 0)= abs(linearSample.twoSite.periods(...
fullPeriodMeans2l == 0)) < periodTol;
  periodMask2l= squeeze(all(periodMask2l, 1))';
  clear fullPeriodMeans2l;
 % Amplitude mask
  amplitudeMask2l= squeeze(all(amplitudeThresh <= linearSample.twoSite.amplitudes, 1))';
 % Combine masks and find periodic proportion
  fullOscMask2l= periodMask2l & amplitudeMask2l;
  oscMask2l= any(fullOscMask2l, 2);
  oscIndices2l= find(oscMask2l);
  oscProp2l= length(oscIndices2l)/numSamples;
  disp([ 'Periodic proportion: ' num2str(length(oscIndices2l)) '/' num2str(numSamples) '...
= ' num2str(oscProp2l) ]);

  [maxArray2l, maxIndices2l]= max(fullOscMask2l.*periodMeans2l, [], 2);
  globalMax2l= max(maxArray2l);
  minArray2l= zeros(numSamples, 1);
  minIndices2l= ones(numSamples, 1);
  for k= 1:numSamples
    positiveIndices= fullOscMask2l(k,:) > 0;
    if any(positiveIndices)
      [minArray2l(k), minIndices2l(k)]= min(periodMeans2l(k, positiveIndices));
    end
  end
  globalMin2l= min(minArray2l(0 < minArray2l));
```

```
  if isempty(globalMin2l)
    disp('No period range.');
  else
    disp([ 'Nontrivial period range: [' num2str(globalMin2l) ', ' num2str(globalMax2l) '...
]' ]);
  end

  oscH13TotalSweepIndicesMin2l= minIndices2l(0 < minArray2l);
  oscH13TotalSweepIndicesMax2l= maxIndices2l(0 < maxArray2l);
  oscH13TotalMinValues2l= nan(min(length(oscH13TotalSweepIndicesMin2l), ...
length(oscH13TotalSweepIndicesMax2l)),1);
  oscH13TotalMaxValues2l= oscH13TotalMinValues2l;
  if (length(oscH13TotalSweepIndicesMin2l) ~= length(oscH13TotalSweepIndicesMax2l))
    disp('WARNING: Different length min/max vectors for total control protein.');
  else
    for k= 1:length(oscH13TotalSweepIndicesMin2l)
     % Note that the ordering of the max and min period is not guaranteed
      oscH13TotalMinValues2l(k)= min(...
nonlinearSample.params.H13TotalMaxSweep(oscH13TotalSweepIndicesMin2l(k)), ...
nonlinearSample.params.H13TotalMaxSweep(oscH13TotalSweepIndicesMax2l(k)));
      oscH13TotalMaxValues2l(k)= max(...
nonlinearSample.params.H13TotalMaxSweep(oscH13TotalSweepIndicesMin2l(k)), ...
nonlinearSample.params.H13TotalMaxSweep(oscH13TotalSweepIndicesMax2l(k)));
    end
  end

  [deltaMax2l, deltaMaxIndex2l]= max(maxArray2l - minArray2l);
  if ~isempty(oscIndices2l)
    disp([ 'Maximum Delta T: [' num2str(minArray2l(deltaMaxIndex2l)) ', ' num2str(...
maxArray2l(deltaMaxIndex2l)) '], Delta T= ' num2str(deltaMax2l) ', k= ' num2str(...
deltaMaxIndex2l) ', j= [' num2str(minIndices2l(deltaMaxIndex2l)) ', ' num2str(...
maxIndices2l(deltaMaxIndex2l)) ']' ]);
  else
    disp('Maximum Delta T: NaN');
  end

  periodOscMask2l= (minArray2l <= targetPeriod + deltaPeriod) & (targetPeriod - ...
deltaPeriod <= maxArray2l);
  periodOscIndices2l= find(periodOscMask2l);
  periodOscProp2l= length(periodOscIndices2l)/numSamples;
  disp([ num2str(targetPeriod) ' +/- ' num2str(deltaPeriod) ' min period proportion: ' ...
num2str(length(periodOscIndices2l)) '/' num2str(numSamples) '= ' num2str(periodOscProp2l)...
 ]);

  periodMaxArray2l= periodOscMask2l.*maxArray2l;
  periodMaxIndices2l= maxIndices2l;
```

```
  periodMaxIndices2l(periodOscMask2l == 0)= 1;
  periodMinArray2l= periodOscMask2l.*minArray2l;
  periodMinIndices2l= minIndices2l;
  periodMinIndices2l(periodOscMask2l == 0)= 1;
  periodDeltaArray2l= periodMaxArray2l - periodMinArray2l;
  [periodDeltaMax2l, periodDeltaMaxIndex2l]= max(periodDeltaArray2l);
  if ~isempty(periodOscIndices2l)
    disp(['Maximum Delta T containing ' num2str(targetPeriod) ' +/- ' num2str(...
deltaPeriod) ' : [' num2str(periodMinArray2l(periodDeltaMaxIndex2l)) ', ' num2str(...
periodMaxArray2l(periodDeltaMaxIndex2l)) '], Delta T= ' num2str(periodDeltaMax2l) ', k= ...
' num2str(periodDeltaMaxIndex2l) ', j= [' num2str(periodMinIndices2l(...
periodDeltaMaxIndex2l)) ',' num2str(periodMaxIndices2l(periodDeltaMaxIndex2l)) '] @' 10 ...
' rho= ' num2str(linearSample.params.rhoSample(periodDeltaMaxIndex2l)) ', kH13H13= ' ...
num2str(linearSample.params.kH13H13Sample(periodDeltaMaxIndex2l)) ', kH7H7= ' num2str(...
linearSample.params.kH7H7Sample(periodDeltaMaxIndex2l)) ', kH7H13= ' num2str(linearSample...
.params.kH7H13Sample(periodDeltaMaxIndex2l)) ', tau_h7= ' num2str(linearSample.params....
tau_h7Sample(periodDeltaMaxIndex2l)) ', bH7= ' num2str(linearSample.params.bH7Sample(...
periodDeltaMaxIndex2l)) ', rH7H7= ' num2str(linearSample.params.rH7H7Sample(...
periodDeltaMaxIndex2l)) ', wH7H7_H7H7= ' num2str(linearSample.params.wH7H7_H7H7Sample(...
periodDeltaMaxIndex2l)) ', rN= ' num2str(linearSample.params.rNSample(...
periodDeltaMaxIndex2l))]);
  else
    disp(['Maximum Delta T containing ' num2str(targetPeriod) ' +/- ' num2str(...
deltaPeriod) ' : NaN' ]);
  end

  periodOscH13TotalSweepIndicesMin2l= periodMinIndices2l(0 < periodMinArray2l);
  periodOscH13TotalSweepIndicesMax2l= periodMaxIndices2l(0 < periodMaxArray2l);
  periodOscH13TotalMinValues2l= nan(min(length(periodOscH13TotalSweepIndicesMin2l), ...
length(periodOscH13TotalSweepIndicesMax2l)),1);
  periodOscH13TotalMaxValues2l= periodOscH13TotalMinValues2l;
  if (length(periodOscH13TotalSweepIndicesMin2l) ~= length(...
periodOscH13TotalSweepIndicesMax2l))
    disp('WARNING: Different length min/max vectors for total control protein.');
  else
    for k= 1:length(periodOscH13TotalSweepIndicesMin2l)
     % Note that the ordering of the max and min period is not guaranteed
      periodOscH13TotalMinValues2l(k)= min(...
nonlinearSample.params.H13TotalMaxSweep(periodOscH13TotalSweepIndicesMin2l(k)), ...
nonlinearSample.params.H13TotalMaxSweep(periodOscH13TotalSweepIndicesMax2l(k)));
      periodOscH13TotalMaxValues2l(k)= max(...
nonlinearSample.params.H13TotalMaxSweep(periodOscH13TotalSweepIndicesMin2l(k)), ...
nonlinearSample.params.H13TotalMaxSweep(periodOscH13TotalSweepIndicesMax2l(k)));
    end
  end
```

```
  halfTargetDeltaIndices2l= ...
periodOscIndices2l(targetDelta/2 <= periodDeltaArray2l(periodOscIndices2l));
  halfTargetDeltaH13TotalSweepIndicesMin2l= periodMinIndices2l(halfTargetDeltaIndices2l);
  halfTargetDeltaH13TotalSweepIndicesMax2l= periodMaxIndices2l(halfTargetDeltaIndices2l);
  halfTargetDeltaH13TotalMinValues2l= nan(min(length(...
halfTargetDeltaH13TotalSweepIndicesMin2l), ...
length(halfTargetDeltaH13TotalSweepIndicesMax2l)),1);
  halfTargetDeltaH13TotalMaxValues2l= halfTargetDeltaH13TotalMinValues2l;
  if (length(halfTargetDeltaH13TotalSweepIndicesMin2l) ~= length(...
halfTargetDeltaH13TotalSweepIndicesMax2l))
    disp('WARNING: Different length min/max vectors for total control protein.');
  else
    for k= 1:length(halfTargetDeltaH13TotalSweepIndicesMin2l)
     % Note that the ordering of the max and min period is not guaranteed
      halfTargetDeltaH13TotalMinValues2l(k)= min(...
nonlinearSample.params.H13TotalMaxSweep(halfTargetDeltaH13TotalSweepIndicesMin2l(k)), ...
nonlinearSample.params.H13TotalMaxSweep(halfTargetDeltaH13TotalSweepIndicesMax2l(k)));
      halfTargetDeltaH13TotalMaxValues2l(k)= max(...
nonlinearSample.params.H13TotalMaxSweep(halfTargetDeltaH13TotalSweepIndicesMin2l(k)), ...
nonlinearSample.params.H13TotalMaxSweep(halfTargetDeltaH13TotalSweepIndicesMax2l(k)));
    end
  end

  periodDeltaCountFuncH2l= @(deltaTMin) nnz(deltaTMin <= periodDeltaArray2l(...
periodOscIndices2l));
  output.linearDecay.twoSiteIndices= periodOscIndices2l(targetDelta <= periodDeltaArray2l...
(periodOscIndices2l))';

  disp([num2str(targetPeriod) ' +/- ' num2str(deltaPeriod) ' min period proportion with...
 Delta T >= ' num2str(targetDelta) ': ' num2str(periodDeltaCountFuncH2l(targetDelta)) '/...
' num2str(numSamples) ' = ' num2str(periodDeltaCountFuncH2l(targetDelta)/numSamples)]);

% Figures for main paper
 % Model selection figure
  figureH= figure(1); clf(figureH); set(figureH, 'Position', [45 305 750 225]);
%    colormap([0.25 0.25 0.25; 0.75 0.75 0.75]); % B&W colors
    axisH= subplot(1,3,1, 'Position', [0.09 0.18 0.22 2/3]);
      bar(axisH, 100*[oscProp1n periodOscProp1n; oscProp1l periodOscProp1l; oscProp2n ...
periodOscProp2n; oscProp2l periodOscProp2l]);
      ylim(axisH, [0 100]);
      set(axisH, 'YTick', 0:20:100);
      set(axisH, 'YMinorTick','On');
      ylabel(axisH, '% of Simulations', 'FontWeight','demi', 'FontSize',12);
      xlim(axisH, [0.5 4.5]);
      xlabel(axisH, 'Model Scenario', 'FontWeight','demi', 'FontSize',12);
      set(axisH, 'XTickLabel', {'I' 'II' 'III' 'IV'});
```

```
      legend(axisH, 'Periodic', [ num2str(targetPeriod) '\pm' num2str(deltaPeriod) '␣min...
.'], 'Location','NorthWest');
      title(axisH, 'Scenario␣Comparison', 'FontWeight','demi', 'FontSize',12);
      set(axisH, 'FontWeight','demi');

    axisH= subplot(1,3,2, 'Position', [0.42 0.18 0.22 2/3]);
      xEdges= 0:2.5:20;
      xMidpoints= xEdges(1:end-1) + 1.25;
      count2n= zeros(length(xMidpoints),1);
      count2l= count2n;
      for k= 1:length(xMidpoints)
        count2n(k)= periodDeltaCountFuncH2n(xEdges(k)) - periodDeltaCountFuncH2n(xEdges(k...
+1));
        count2l(k)= periodDeltaCountFuncH2l(xEdges(k)) - periodDeltaCountFuncH2l(xEdges(k...
+1));
      end
      bar(axisH, xMidpoints, 100/numSamples*[count2n count2l], 'BarLayout','stacked', '...
BarWidth',1);
      ylabel(axisH, '%␣of␣Simulations', 'FontWeight','demi', 'FontSize',12);
      set(axisH, 'YMinorTick','On');
      xlim(axisH, [0 20]);
      set(axisH, 'XTick', 0:2.5:20);
      set(axisH, 'XTickLabel', {'0' '' '5' '' '10' '' '15' '' '20'});
      xlabel(axisH, '{\Delta}T␣(min)', 'FontWeight','demi', 'FontSize',12);
      legend(axisH, 'Scenario␣III', 'Scenario␣IV');
      title(axisH, '{\Delta}T␣Distributions', 'FontWeight','demi', 'FontSize',12);
      set(axisH, 'FontWeight','demi');

     % Tabulations
      disp('␣');
      disp([ 'Model␣Scenario␣III␣Delta␣T␣tabulation␣(out␣of␣' num2str(numSamples) '␣...
samples):']);
      disp([ 'Bin␣edges:␣', mat2str(xEdges) ]);
      disp([ 'Counts␣:␣', mat2str(count2n') ]);
      disp('␣');
      disp([ 'Model␣Scenario␣IV␣Delta␣T␣tabulation␣(out␣of␣' num2str(numSamples) '␣...
samples):' ]);
      disp([ 'Bin␣edges:␣', mat2str(xEdges) ]);
      disp([ 'Counts␣:␣', mat2str(count2l') ]);

    axisH= subplot(1,3,3, 'Position', [0.75 0.18 0.22 2/3]);
      cMap= colormap; % Use same colors as lines in next subplot
      nonlinearMeanPeriods= periodMeans2n(periodDeltaMaxIndex2n,:);
      linearMeanPeriods= periodMeans2l(periodDeltaMaxIndex2l,:);
```

```
      seriesH= plot(axisH, nonlinear␣Sample.params.H13TotalMaxSweep(1:periodMinIndices2n(...
periodDeltaMaxIndex2n)), nonlinearMeanPeriods(1:periodMinIndices2n(periodDeltaMaxIndex2n)...
), '-', linearSample.params.H13TotalMaxSweep(1:periodMinIndices2l(periodDeltaMaxIndex2l))...
, linearMeanPeriods(1:periodMinIndices2l(periodDeltaMaxIndex2l)), '--');
      set(seriesH(1), 'LineWidth',2, 'Color',cMap(1,:));
      set(seriesH(2), 'LineWidth',2, 'Color',cMap(end,:));
      ylabel(axisH, 'Period␣(min)', 'FontWeight','demi', 'FontSize',12);
      set(axisH, 'YMinorTick','On');
      xlabel(axisH, 'Total␣Control␣Protein', 'FontWeight','Demi', 'FontSize',12);
      xlim(axisH, [0 nonlinearSample.params.H13TotalMaxSweep(end)]);
      set(axisH, 'XMinorTick','On');
      title(axisH, '{\Delta}T_{III}^{max}␣and␣{\Delta}T_{IV}^{max}␣', ...
'FontWeight','Demi', 'FontSize',12);
      legend(axisH, 'Scenario␣III', 'Scenario␣IV');
      set(axisH, 'FontWeight','Demi');

 % Coefficient of variability figure
  oscTitle= 'Periodic';
  periodicTitle= [ num2str(targetPeriod) '\pm' num2str(deltaPeriod) '␣min' ];
  halfTargetDeltaTitle= [ '{\Delta}T\geq' num2str(targetDelta/2) '␣min' ];
  targetDeltaTitle= [ '{\Delta}T\geq' num2str(targetDelta) '␣min' ];

  targetDeltaIndices2n= periodDeltaArray2n >= targetDelta;
  halfTargetDeltaIndices2n= periodDeltaArray2n >= targetDelta/2;
  targetDeltaIndices2l= periodDeltaArray2l >= targetDelta;
  halfTargetDeltaIndices2l= periodDeltaArray2l >= targetDelta/2;

  figureH= figure(2); clf(figureH); set(figureH, 'Position', [100 100 722 290]);
  for subPlotIndex= 1:2
    if subPlotIndex == 1 % Scenario III
      scenario= 'III';
      oscIndices= oscIndices2n;
      periodOscIndices= periodOscIndices2n;
      halfTargetDeltaIndices= halfTargetDeltaIndices2n;
      targetDeltaIndices= targetDeltaIndices2n;
    else % Scenario IV
      scenario= 'IV';
      oscIndices= oscIndices2l;
      periodOscIndices= periodOscIndices2l;
      halfTargetDeltaIndices= halfTargetDeltaIndices2l;
      targetDeltaIndices= targetDeltaIndices2l;
    end

    axisH= subplot(1,2,subPlotIndex);
      oscCV= [ ...
std(nonlinearSample.params.rhoSample(oscIndices))/...
```

```
mean(nonlinearSample.params.rhoSample(oscIndices)), ...
std(nonlinearSample.params.tau_h7Sample(oscIndices))/...
mean(nonlinearSample.params.tau_h7Sample(oscIndices)), ...
std(nonlinearSample.params.rH7H7Sample(oscIndices))/...
mean(nonlinearSample.params.rH7H7Sample(oscIndices)), ...
std(nonlinearSample.params.wH7H7_H7H7Sample(oscIndices))/...
mean(nonlinearSample.params.wH7H7_H7H7Sample(oscIndices)), ...
std(nonlinearSample.params.bH7Sample(oscIndices))/...
mean(nonlinearSample.params.bH7Sample(oscIndices)), ...
std(nonlinearSample.params.kH13H13Sample(oscIndices))/...
mean(nonlinearSample.params.kH13H13Sample(oscIndices)), ...
std(nonlinearSample.params.kH7H7Sample(oscIndices))/...
mean(nonlinearSample.params.kH7H7Sample(oscIndices)), ...
std(nonlinearSample.params.kH7H13Sample(oscIndices))/...
mean(nonlinearSample.params.kH7H13Sample(oscIndices)) ];
      periodOscCV= [ ...
std(nonlinearSample.params.rhoSample(periodOscIndices))/...
mean(nonlinearSample.params.rhoSample(periodOscIndices)), ...
std(nonlinearSample.params.tau_h7Sample(periodOscIndices))/...
mean(nonlinearSample.params.tau_h7Sample(periodOscIndices)), ...
std(nonlinearSample.params.rH7H7Sample(periodOscIndices))/...
mean(nonlinearSample.params.rH7H7Sample(periodOscIndices)), ...
std(nonlinearSample.params.wH7H7_H7H7Sample(periodOscIndices))/...
mean(nonlinearSample.params.wH7H7_H7H7Sample(periodOscIndices)), ...
std(nonlinearSample.params.bH7Sample(periodOscIndices))/...
mean(nonlinearSample.params.bH7Sample(periodOscIndices)), ...
std(nonlinearSample.params.kH13H13Sample(periodOscIndices))/...
mean(nonlinearSample.params.kH13H13Sample(periodOscIndices)), ...
std(nonlinearSample.params.kH7H7Sample(periodOscIndices))/...
mean(nonlinearSample.params.kH7H7Sample(periodOscIndices)), ...
std(nonlinearSample.params.kH7H13Sample(periodOscIndices))/...
mean(nonlinearSample.params.kH7H13Sample(periodOscIndices)) ];
      halfTargetDeltaCV= [ ...
std(nonlinearSample.params.rhoSample(halfTargetDeltaIndices))/...
mean(nonlinearSample.params.rhoSample(halfTargetDeltaIndices)), ...
std(nonlinearSample.params.tau_h7Sample(halfTargetDeltaIndices))/...
mean(nonlinearSample.params.tau_h7Sample(halfTargetDeltaIndices)), ...
std(nonlinearSample.params.rH7H7Sample(halfTargetDeltaIndices))/...
mean(nonlinearSample.params.rH7H7Sample(halfTargetDeltaIndices)), ...
std(nonlinearSample.params.wH7H7_H7H7Sample(halfTargetDeltaIndices))/...
mean(nonlinearSample.params.wH7H7_H7H7Sample(halfTargetDeltaIndices)), ...
std(nonlinearSample.params.bH7Sample(halfTargetDeltaIndices))/...
mean(nonlinearSample.params.bH7Sample(halfTargetDeltaIndices)), ...
std(nonlinearSample.params.kH13H13Sample(halfTargetDeltaIndices))/...
mean(nonlinearSample.params.kH13H13Sample(halfTargetDeltaIndices)), ...
std(nonlinearSample.params.kH7H7Sample(halfTargetDeltaIndices))/...
```

```
mean(nonlinearSample.params.kH7H7Sample(halfTargetDeltaIndices)), ...
std(nonlinearSample.params.kH7H13Sample(halfTargetDeltaIndices))/...
mean(nonlinearSample.params.kH7H13Sample(halfTargetDeltaIndices)) ];
      targetDeltaCV= [ ...
std(nonlinearSample.params.rhoSample(targetDeltaIndices))/...
mean(nonlinearSample.params.rhoSample(targetDeltaIndices)), ...
std(nonlinearSample.params.tau_h7Sample(targetDeltaIndices))/...
mean(nonlinearSample.params.tau_h7Sample(targetDeltaIndices)), ...
std(nonlinearSample.params.rH7H7Sample(targetDeltaIndices))/...
mean(nonlinearSample.params.rH7H7Sample(targetDeltaIndices)), ...
std(nonlinearSample.params.wH7H7_H7H7Sample(targetDeltaIndices))/...
mean(nonlinearSample.params.wH7H7_H7H7Sample(targetDeltaIndices)), ...
std(nonlinearSample.params.bH7Sample(targetDeltaIndices))/...
mean(nonlinearSample.params.bH7Sample(targetDeltaIndices)), ...
std(nonlinearSample.params.kH13H13Sample(targetDeltaIndices))/...
mean(nonlinearSample.params.kH13H13Sample(targetDeltaIndices)), ...
std(nonlinearSample.params.kH7H7Sample(targetDeltaIndices))/...
mean(nonlinearSample.params.kH7H7Sample(targetDeltaIndices)), ...
std(nonlinearSample.params.kH7H13Sample(targetDeltaIndices))/...
mean(nonlinearSample.params.kH7H13Sample(targetDeltaIndices)) ];
      indices= 1:8; % Index the eight estimated parameters
      labels= {'\rho', '\tau_c', '\rho_{C:C}', '\omega_{C:C}', '\beta_C', ...
'\kappa_{G:G}', '\kappa_{C:C}', '\kappa_{C:G}'};
      bar(axisH, [oscCV' periodOscCV' halfTargetDeltaCV' targetDeltaCV']);
%       plot(axisH, indices, oscCV, 'kx', indices, periodOscCV, 'kd', ...
%indices, halfTargetDeltaCV, 'ks', indices, targetDeltaCV, 'ko');
      title(axisH, [ 'Parameter␣Sensitivity,␣Scenario␣' scenario ], ...
'FontWeight','demi', 'FontSize',12);
      ylabel(axisH, 'Coefficient␣of␣Variation', 'FontWeight','demi', 'FontSize',12);
      xlim(axisH, [indices(1) - 0.5, indices(end) + 0.5]);
      xlabel(axisH, 'Parameter', 'FontWeight','demi', 'FontSize',12, ...
'Position', [4.5, -0.15, 1]);
      set(axisH, 'XTick',indices)
      set(axisH, 'XTickLabel',''); % Clear XTickLabels
      for index= indices
        text(index, -0.08, labels{index}, 'FontWeight','demi', ...
'FontSize',12, 'HorizontalAlignment','center');
      end
      legend(axisH, {oscTitle, periodicTitle, halfTargetDeltaTitle, ...
targetDeltaTitle}, 'Location','Best');
      set(axisH, 'FontWeight', 'Demi');
  end

 % Histogram plots
  for scenarioIndex= 1:4
    figureH= figure(1 + 2*scenarioIndex);
```

```
clf(figureH);
colormap([0.5 0.5 0.5]);

switch scenarioIndex
  case 1 % Scenario I
    scenario= 'I';
    rows= 1;
    cols= 3;
    oscIndices= oscIndices1n;
    oscH13TotalMinValues= oscH13TotalMinValues1n;
    oscH13TotalMaxValues= oscH13TotalMaxValues1n;
%        periodOscIndices= periodOscIndices1n;
%        periodOscH13TotalMinValues= periodOscH13TotalMinValues1n;
%        periodOscH13TotalMaxValues= periodOscH13TotalMaxValues1n;
%        halfTargetDeltaIndices= halfTargetDeltaIndices1n;
%        targetDeltaIndices= targetDeltaIndices1n;
    set(figureH, 'Position', [0 100 512*3/4 560/4]);
  case 2 % Scenario II
    scenario= 'II';
    rows= 2;
    cols= 3;
    oscIndices= oscIndices1l;
    oscH13TotalMinValues= oscH13TotalMinValues1l;
    oscH13TotalMaxValues= oscH13TotalMaxValues1l;
    periodOscIndices= periodOscIndices1l;
    periodOscH13TotalMinValues= periodOscH13TotalMinValues1l;
    periodOscH13TotalMaxValues= periodOscH13TotalMaxValues1l;
%        halfTargetDeltaIndices= halfTargetDeltaIndices1l;
%        targetDeltaIndices= targetDeltaIndices1l;
    set(figureH, 'Position', [100 100 512*3/4 560/2]);
  case 3 % Scenario III
    scenario= 'III';
    rows= 4;
    cols= 4;
    oscIndices= oscIndices2n;
    oscH13TotalMinValues= oscH13TotalMinValues2n;
    oscH13TotalMaxValues= oscH13TotalMaxValues2n;
    periodOscIndices= periodOscIndices2n;
    periodOscH13TotalMinValues= periodOscH13TotalMinValues2n;
    periodOscH13TotalMaxValues= periodOscH13TotalMaxValues2n;
    halfTargetDeltaIndices= halfTargetDeltaIndices2n;
    halfTargetDeltaH13TotalMinValues= halfTargetDeltaH13TotalMinValues2n;
    halfTargetDeltaH13TotalMaxValues= halfTargetDeltaH13TotalMaxValues2n;
    targetDeltaIndices= targetDeltaIndices2n;
    targetDeltaH13TotalMinValues= targetDeltaH13TotalMinValues2n;
    targetDeltaH13TotalMaxValues= targetDeltaH13TotalMaxValues2n;
```

```
      set(figureH, 'Position', [200 100 512 560]);
    case 4 % Scenario IV
      scenario= 'IV';
      rows= 3;
      cols= 4;
      oscIndices= oscIndices2l;
      oscH13TotalMinValues= oscH13TotalMinValues2l;
      oscH13TotalMaxValues= oscH13TotalMaxValues2l;
      periodOscIndices= periodOscIndices2l;
      periodOscH13TotalMinValues= periodOscH13TotalMinValues2l;
      periodOscH13TotalMaxValues= periodOscH13TotalMaxValues2l;
      halfTargetDeltaIndices= halfTargetDeltaIndices2l;
      halfTargetDeltaH13TotalMinValues= halfTargetDeltaH13TotalMinValues2l;
      halfTargetDeltaH13TotalMaxValues= halfTargetDeltaH13TotalMaxValues2l;
%       targetDeltaIndices= targetDeltaIndices2l;
      set(figureH, 'Position', [300 100 512 560*3/4]);
  end

  k= 1; % Subplot counter
  if rows >= 1
  axisH= subplot(rows,cols,k);
    edges= 1/3:1/3:3;
    bar(edges, histc(nonlinearSample.params.rhoSample(oscIndices), edges), 'histc');
    xlim([0.99/3 3]);
    set(axisH, 'XTick', [0.33 1 2 3]);
    xlabel('\rho');
    title(oscTitle);
    k= k + 1;
  axisH= subplot(rows,cols,k);
    edges= 2:0.5:9;
    bar(edges, histc(nonlinearSample.params.tau_h7Sample(oscIndices), edges), 'histc');
    xlim([2.3 8.1]);
    set(axisH, 'XTick', [2.3 4:1:6 8.1]);
    xlabel('\tau_c');
    title(oscTitle);
    k= k + 1;
  axisH= subplot(rows,cols,k);
    edges= 0:0.1:1;
    bar(edges, histc(nonlinearSample.params.rH7H7Sample(oscIndices), edges), 'histc');
    xlim([0.01 1]);
    set(axisH, 'XTick', [0.01 0.5 1]);
    xlabel('\rho_{C:C}');
    title(oscTitle);
    k= k + 1;
  if cols >= 4
  axisH= subplot(rows,cols,k);
```

```
      edges= 0:10:100;
      bar(edges, histc(nonlinearSample.params.wH7H7_H7H7Sample(oscIndices), edges), '...
histc');
      xlim([1 100]);
      set(axisH, 'XTick', [1 50 100]);
      xlabel('\omega_{C:C}');
      title(oscTitle);
      k= k + 1;
    end
    end
    if rows >= 2
    axisH= subplot(rows,cols,k);
      edges= 1/3:1/3:3;
      bar(edges, histc(nonlinearSample.params.rhoSample(periodOscIndices), edges), '...
histc');
      xlim([0.99/3 3]);
      set(axisH, 'XTick', [0.33 1 2 3]);
      xlabel('\rho');
      title(periodicTitle);
      k= k + 1;
    axisH= subplot(rows,cols,k);
      edges= 2:0.5:9;
      bar(edges, histc(nonlinearSample.params.tau_h7Sample(periodOscIndices), edges), '...
histc');
      xlim([2.3 8.1]);
      set(axisH, 'XTick', [2.3 4:1:6 8.1]);
      xlabel('\tau_c');
      title(periodicTitle);
      k= k + 1;
    axisH= subplot(rows,cols,k);
      edges= 0:0.1:1;
      bar(edges, histc(nonlinearSample.params.rH7H7Sample(periodOscIndices), edges), '...
histc');
      xlim([0.01 1]);
      set(axisH, 'XTick', [0.01 0.5 1]);
      xlabel('\rho_{C:C}');
      title(periodicTitle);
      k= k + 1;
    if cols >= 4
    axisH= subplot(rows,cols,k);
      edges= 0:10:100;
      bar(edges, histc(nonlinearSample.params.wH7H7_H7H7Sample(periodOscIndices), edges),...
 'histc');
      xlim([1 100]);
      set(axisH, 'XTick', [1 50 100]);
      xlabel('\omega_{C:C}');
```

```
      title(periodicTitle);
      k= k + 1;
    end
    end
    if rows >= 3
    axisH= subplot(rows,cols,k);
      edges= 1/3:1/3:3;
      bar(edges, histc(nonlinearSample.params.rhoSample(halfTargetDeltaIndices), edges), ...
'histc');
      xlim([0.99/3 3]);
      set(axisH, 'XTick', [0.33 1 2 3]);
      xlabel('\rho');
      title(halfTargetDeltaTitle);
      k= k + 1;
    axisH= subplot(rows,cols,k);
      edges= 2:0.5:9;
      bar(edges, histc(nonlinearSample.params.tau_h7Sample(halfTargetDeltaIndices), edges...
), 'histc');
      xlim([2.3 8.1]);
      set(axisH, 'XTick', [2.3 4:1:6 8.1]);
      xlabel('\tau_c');
      title(halfTargetDeltaTitle);
      k= k + 1;
    axisH= subplot(rows,cols,k);
      edges= 0:0.1:1;
      bar(edges, histc(nonlinearSample.params.rH7H7Sample(halfTargetDeltaIndices), edges)...
, 'histc');
      xlim([0.01 1]);
      set(axisH, 'XTick', [0.01 0.5 1]);
      xlabel('\rho_{C:C}');
      title(halfTargetDeltaTitle);
      k= k + 1;
    if cols >= 4
    axisH= subplot(rows,cols,k);
      edges= 0:10:100;
      bar(edges, histc(nonlinearSample.params.wH7H7_H7H7Sample(halfTargetDeltaIndices), ...
edges), 'histc');
      xlim([1 100]);
      set(axisH, 'XTick', [1 50 100]);
      xlabel('\omega_{C:C}');
      title(halfTargetDeltaTitle);
      k= k + 1;
    end
    end
    if rows >= 4
    axisH= subplot(rows,cols,k);
```

```
      edges= 1/3:1/3:3;
      bar(edges, histc(nonlinearSample.params.rhoSample(targetDeltaIndices), edges), '...
histc');
      xlim([0.99/3 3]);
      set(axisH, 'XTick', [0.33 1 2 3]);
      xlabel('\rho');
      title(targetDeltaTitle);
      k= k + 1;
    axisH= subplot(rows,cols,k);
      edges= 2:0.5:9;
      bar(edges, histc(nonlinearSample.params.tau_h7Sample(targetDeltaIndices), edges), ...
'histc');
      xlim([2.3 8.1]);
      set(axisH, 'XTick', [2.3 4:1:6 8.1]);
      xlabel('\tau_c');
      title(targetDeltaTitle);
      k= k + 1;
    axisH= subplot(rows,cols,k);
      edges= 0:0.1:1;
      bar(edges, histc(nonlinearSample.params.rH7H7Sample(targetDeltaIndices), edges), '...
histc');
      xlim([0.01 1]);
      set(axisH, 'XTick', [0.01 0.5 1]);
      xlabel('\rho_{C:C}');
      title(targetDeltaTitle);
      k= k + 1;
    if cols >= 4
      axisH= subplot(rows,cols,k);
      edges= 0:10:100;
      bar(edges, histc(nonlinearSample.params.wH7H7_H7H7Sample(targetDeltaIndices), edges...
), 'histc');
      xlim([1 100]);
      set(axisH, 'XTick', [1 50 100]);
      xlabel('\omega_{C:C}');
      title(targetDeltaTitle);
    end
    end

    figureH= figure(2 + 2*scenarioIndex);
    clf(figureH);
    colormap([0.5 0.5 0.5]);
    switch scenarioIndex
      case 1 % Scenario I
        set(figureH, 'Position', [50 100 512 560/4]);
      case 2 % Scenario II
        set(figureH, 'Position', [150 100 512 560/2]);
```

```
      case 3 % Scenario III
        set(figureH, 'Position', [250 100 512 560]);
      case 4 % Scenario IV
        set(figureH, 'Position', [350 100 512 560*3/4]);
    end

    if rows >= 1
    axisH= subplot(rows,4,1);
      edges= 0.2:0.05:0.5;
      bar(edges, histc(nonlinearSample.params.bH7Sample(oscIndices), edges), 'histc');
      xlim([0.2 0.5]);
      set(axisH, 'XTick', [0.2 0.35 0.5]);
      xlabel('\beta_C');
      title(oscTitle);
    axisH= subplot(rows,4,2);
      edges= 0:50:1000;
      bar(edges, histc(nonlinearSample.params.kH13H13Sample(oscIndices), edges), 'histc')...
;
      xlim([10 1000]);
      set(axisH, 'XTick', [10 500 1000]);
      xlabel('\kappa_{G:G}');
      title(oscTitle);
    axisH= subplot(rows,4,3);
      edges= 0:50:1000;
      bar(edges, histc(nonlinearSample.params.kH7H7Sample(oscIndices), edges), 'histc');
      xlim([10 1000]);
      set(axisH, 'XTick', [10 500 1000]);
      xlabel('\kappa_{C:C}');
      title(oscTitle);
    axisH= subplot(rows,4,4);
      edges= 0:50:1000;
      bar(edges, histc(nonlinearSample.params.kH7H13Sample(oscIndices), edges), 'histc');
      xlim([10 1000]);
      set(axisH, 'XTick', [10 500 1000]);
      xlabel('\kappa_{C:G}');
      title(oscTitle);
    end
    if rows >= 2
    axisH= subplot(rows,4,5);
      edges= 0.2:0.05:0.5;
      bar(edges, histc(nonlinearSample.params.bH7Sample(periodOscIndices), edges), '...
histc');
      xlim([0.2 0.5]);
      set(axisH, 'XTick', [0.2 0.35 0.5]);
      xlabel('\beta_C');
      title(periodicTitle);
```

```
    axisH= subplot(rows,4,6);
      edges= 0:50:1000;
      bar(edges, histc(nonlinearSample.params.kH13H13Sample(periodOscIndices), edges), '...
histc');
      xlim([10 1000]);
      set(axisH, 'XTick', [10 500 1000]);
      xlabel('\kappa_{G:G}');
      title(periodicTitle);
    axisH= subplot(rows,4,7);
      edges= 0:50:1000;
      bar(edges, histc(nonlinearSample.params.kH7H7Sample(periodOscIndices), edges), '...
histc');
      xlim([10 1000]);
      set(axisH, 'XTick', [10 500 1000]);
      xlabel('\kappa_{C:C}');
      title(periodicTitle);
    axisH= subplot(rows,4,8);
      edges= 0:50:1000;
      bar(edges, histc(nonlinearSample.params.kH7H13Sample(periodOscIndices), edges), '...
histc');
      xlim([10 1000]);
      set(axisH, 'XTick', [10 500 1000]);
      xlabel('\kappa_{C:G}');
      title(periodicTitle);
    end
    if rows >= 3
    axisH= subplot(rows,4,9);
      edges= 0.2:0.05:0.5;
      bar(edges, histc(nonlinearSample.params.bH7Sample(halfTargetDeltaIndices), edges), ...
'histc');
      xlim([0.2 0.5]);
      set(axisH, 'XTick', [0.2 0.35 0.5]);
      xlabel('\beta_C');
      title(halfTargetDeltaTitle);
    axisH= subplot(rows,4,10);
      edges= 0:50:1000;
      bar(edges, histc(nonlinearSample.params.kH13H13Sample(halfTargetDeltaIndices), ...
edges), 'histc');
      xlim([10 1000]);
      set(axisH, 'XTick', [10 500 1000]);
      xlabel('\kappa_{G:G}');
      title(halfTargetDeltaTitle);
    axisH= subplot(rows,4,11);
      edges= 0:50:1000;
      bar(edges, histc(nonlinearSample.params.kH7H7Sample(halfTargetDeltaIndices), edges)...
, 'histc');
```

```
      xlim([10 1000]);
      set(axisH, 'XTick', [10 500 1000]);
      xlabel('\kappa_{C:C}');
      title(halfTargetDeltaTitle);
    axisH= subplot(rows,4,12);
      edges= 0:50:1000;
      bar(edges, histc(nonlinearSample.params.kH7H13Sample(halfTargetDeltaIndices), edges...
), 'histc');
      xlim([10 1000]);
      set(axisH, 'XTick', [10 500 1000]);
      xlabel('\kappa_{C:G}');
      title(halfTargetDeltaTitle);
    end
    if rows >= 4
    axisH= subplot(rows,4,13);
      edges= 0.2:0.05:0.5;
      bar(edges, histc(nonlinearSample.params.bH7Sample(targetDeltaIndices), edges), '...
histc');
      xlim([0.2 0.5]);
      set(axisH, 'XTick', [0.2 0.35 0.5]);
      xlabel('\beta_C');
      title(targetDeltaTitle);
    axisH= subplot(rows,4,14);
      edges= 0:50:1000;
      bar(edges, histc(nonlinearSample.params.kH13H13Sample(targetDeltaIndices), edges), ...
'histc');
      xlim([10 1000]);
      set(axisH, 'XTick', [10 500 1000]);
      xlabel('\kappa_{G:G}');
      title(targetDeltaTitle);
    axisH= subplot(rows,4,15);
      edges= 0:50:1000;
      bar(edges, histc(nonlinearSample.params.kH7H7Sample(targetDeltaIndices), edges), '...
histc');
      xlim([10 1000]);
      set(axisH, 'XTick', [10 500 1000]);
      xlabel('\kappa_{C:C}');
      title(targetDeltaTitle);
    axisH= subplot(rows,4,16);
      edges= 0:50:1000;
      bar(edges, histc(nonlinearSample.params.kH7H13Sample(targetDeltaIndices), edges), ...
'histc');
      xlim([10 1000]);
      set(axisH, 'XTick', [10 500 1000]);
      xlabel('\kappa_{C:G}');
      title(targetDeltaTitle);
```

```
end

figureH= figure(10 + scenarioIndex);
clf(figureH);
colormap([0.5 0.5 0.5]);
switch scenarioIndex
  case 1 % Scenario I
    set(figureH, 'Position', [75 100 512 560/4]);
  case 2 % Scenario II
    set(figureH, 'Position', [175 100 512 560/2]);
  case 3 % Scenario III
    set(figureH, 'Position', [275 100 512 560]);
  case 4 % Scenario IV
    set(figureH, 'Position', [375 100 512 560*3/4]);
end
edges= 0:5:2500;

if rows >= 1
axisH= subplot(rows,2,1);
  bar(edges, histc(oscH13TotalMinValues, edges), 'histc');
  xlim([0 2500]);
  set(axisH, 'XTick', [0 500 1000 1500 2000 2500]);
  xlabel('$\widehat{G}^{\rm␣min}$', 'Interpreter','latex');
  title(oscTitle);
axisH= subplot(rows,2,2);
  bar(edges, histc(oscH13TotalMaxValues, edges), 'histc');
  xlim([0 2500]);
  set(axisH, 'XTick', [0 500 1000 1500 2000 2500]);
  xlabel('$\widehat{G}^{\rm␣max}$', 'Interpreter','latex');
  title(oscTitle);
end
if rows >= 2
axisH= subplot(rows,2,3);
  bar(edges, histc(periodOscH13TotalMinValues, edges), 'histc');
  xlim([0 2500]);
  set(axisH, 'XTick', [0 500 1000 1500 2000 2500]);
  xlabel('$\widehat{G}^{\rm␣min}$', 'Interpreter','latex');
  title(periodicTitle);
axisH= subplot(rows,2,4);
  bar(edges, histc(periodOscH13TotalMaxValues, edges), 'histc');
  xlim([0 2500]);
  set(axisH, 'XTick', [0 500 1000 1500 2000 2500]);
  xlabel('$\widehat{G}^{\rm␣max}$', 'Interpreter','latex');
  title(periodicTitle);
end
if rows >= 3
```

```
    axisH= subplot(rows,2,5);
      bar(edges, histc(halfTargetDeltaH13TotalMinValues, edges), 'histc');
      xlim([0 2500]);
      set(axisH, 'XTick', [0 500 1000 1500 2000 2500]);
      xlabel('$\widehat{G}^{\rm min}$', 'Interpreter','latex');
      title(halfTargetDeltaTitle);
    axisH= subplot(rows,2,6);
      bar(edges, histc(halfTargetDeltaH13TotalMaxValues, edges), 'histc');
      xlim([0 2500]);
      set(axisH, 'XTick', [0 500 1000 1500 2000 2500]);
      xlabel('$\widehat{G}^{\rm max}$', 'Interpreter','latex');
      title(halfTargetDeltaTitle);
    end
    if rows >= 4
    axisH= subplot(rows,2,7);
      bar(edges, histc(targetDeltaH13TotalMinValues, edges), 'histc');
      xlim([0 2500]);
      set(axisH, 'XTick', [0 500 1000 1500 2000 2500]);
      xlabel('$\widehat{G}^{\rm min}$', 'Interpreter','latex');
      title(targetDeltaTitle);
    axisH= subplot(rows,2,8);
      bar(edges, histc(targetDeltaH13TotalMaxValues, edges), 'histc');
      xlim([0 2500]);
      set(axisH, 'XTick', [0 500 1000 1500 2000 2500]);
      xlabel('$\widehat{G}^{\rm max}$', 'Interpreter','latex');
      title(targetDeltaTitle);
    end
  end
end
```

1.3. Plotting Code.

1.3.1. `plotSolution.m`. A simple plotting function called by `somitogenesis.m`.

```
function plotSolution(sol)

% Usage:
%  plotSolution(sol)
%
% Plots various time series for the first cell only

  indices= [sol.params.H7TotalIndices(1); sol.params.h7Indices(1)];
  if sol.params.numGenes == 2
    indices= [indices; sol.params.DIndices(1); sol.params.dIndices(1)];
  end
  startTimeIndex= find(sol.x >= sol.params.timeInterval(1), 1);
  if isempty(startTimeIndex)
```

```
    startTimeIndex= 1;
  end
  maxY= max(max(sol.y(indices, startTimeIndex:end)));

  figure(1);
    axis1= subplot(3,1,1);
      series1= plot(axis1, sol.x, sol.y(indices,:), 'LineWidth',2);
      ylim(axis1, [0, 1.1*maxY]);
      set(axis1, 'FontWeight','demi');
      xlabel('time␣(min)', 'FontSize',12);
      ylabel('Copy␣#', 'FontSize',12);
      if sol.params.couplingBool
        title([num2str(sol.params.numCells, '%d'), '␣Coupled␣Cell(s)␣--␣Cell␣1'], '...
FontSize',14);
      else
        title([num2str(sol.params.numCells, '%d'), '␣Uncoupled␣Cell(s)␣--␣Cell␣1'], '...
FontSize',14);
      end
      if sol.params.numGenes == 1
        legend(series1(end-1:end), ['$H_{\widehat{7}}=␣' num2str(sol.statistics.medians...
(1), '%.1f') '␣\pm' num2str(sol.statistics.amplitudes(1)/2, '%.1f') ';␣' num2str(sol....
statistics.periods(1), '%.1f') '$␣min'], ['$h_7=␣' num2str(sol.statistics.medians(2), '...
%.1f') '␣\pm' num2str(sol.statistics.amplitudes(2)/2, '%.1f') ';␣' num2str(sol.statistics...
.periods(2), '%.1f') '$␣min']);
        set(legend(axis1), 'Location','West', 'Interpreter','latex');
      else % sol.params.numGenes == 2
        legend(series1(end-3:end), ['$H_{\widehat{7}}=␣' num2str(sol.statistics.medians...
(1), '%.1f') '␣\pm' num2str(sol.statistics.amplitudes(1)/2, '%.1f') ';␣' num2str(sol....
statistics.periods(1), '%.1f') '$␣min'], ['$h_7=␣' num2str(sol.statistics.medians(2), '...
%.1f') '␣\pm' num2str(sol.statistics.amplitudes(2)/2, '%.1f') ';␣' num2str(sol.statistics...
.periods(2), '%.1f') '$␣min'], ['$D=␣' num2str(sol.statistics.medians(3), '%.1f') '␣\pm' ...
num2str(sol.statistics.amplitudes(3)/2, '%.1f') ';␣' num2str(sol.statistics.periods(3), ...
'%.1f') '$␣min'], ['$d=␣' num2str(sol.statistics.medians(4), '%.1f') '␣\pm' num2str(sol....
statistics.amplitudes(4)/2, '%.1f') ';␣' num2str(sol.statistics.periods(4), '%.1f') '$␣...
min']);
        set(legend(axis1), 'Location','West', 'Orientation','vertical', 'Interpreter','...
latex');
      end
      xLimits= xlim; % Used in plots below

    axis2= subplot(3,1,2);
      area(axis2, sol.x, [ sol.output.H7H13(1,:)', sol.output.H7H7(1,:)', sol.output.H7...
(1,:)' ]);
      xlim(axis2, xLimits);
      ylim(axis2, [0, 1.1*maxY]);
      set(axis2, 'FontWeight','demi');
```

```
      xlabel('time␣(min)', 'FontSize',12);
      ylabel('Copy␣#', 'FontSize',12);
      title('Her7␣Distribution␣--␣Cell␣1', 'FontSize',14);
      legend('$H_{7:13}$','$H_{7:7}$','$H_7$');
      set(legend(axis2), 'Location','West', 'Interpreter','latex');

    axis3= subplot(3,1,3);
      area(axis3, sol.x, [ sol.output.H7H13(1,:)', sol.output.H13H13(1,:)', sol.output....
H13(1,:)' ]);
      xlim(axis3, xLimits);
      set(axis3, 'FontWeight','demi');
      xlabel('time␣(min)', 'FontSize',12);
      ylabel('Copy␣#', 'FontSize',12);
      title('Her13.2␣Distribution␣--␣Cell␣1', 'FontSize',14);
      legend('$H_{7:13}$','$H_{13:13}$','$H_{13}$');
      set(legend(axis3), 'Location','NorthEast', 'Interpreter','latex');
end
```

1.3.2. `animate.m`. A simple animation function for 1D lines of cells.

```
function animation= animate(sol, tStartPlot, tEndPlot, tDelta, lateralCellNum)

% Usage:
%  animation= animate(sol, tStartPlot, tEndPlot, tDelta, lateralCellNumber)
%
% A QT movie can be recorded by uncommenting the calls to MakeQTMovie()

  if nargin < 5
    lateralCellNum= 1;
  else
    lateralCellNum= max(min(lateralCellNum, sol.params.cellArraySize(2)), 1);
  end
  yStartIndex= (lateralCellNum - 1)*sol.params.cellArraySize(1) + 1;
  yEndIndex= lateralCellNum*sol.params.cellArraySize(1);
  tStartPlot= max([sol.x(1), tStartPlot]); % Domain check
  tStartIndex= find((sol.x(1:end-1) <= tStartPlot) & (sol.x(2:end) > tStartPlot));
  tEndPlot= min([sol.x(end), tEndPlot]); % Domain check
  tEndIndex= find((sol.x(1:end-1) < tEndPlot) & (sol.x(2:end) >= tEndPlot));
  tGrid= tStartPlot : tDelta : tEndPlot; % Assumes tStartPlot < tEndPlot
%{
  for k= 1:sol.params.numCells
    maturityIndices= find(sol.params.somitesInPSM*sol.params.tailbudPeriod + ...
sol.params.birthTimes(k) <= sol.x);
    if ~isempty(maturityIndices)
      for n= (k-1)*sol.params.equationsPerCell+1:k*sol.params.equationsPerCell
        sol.y(n, maturityIndices(1):end)= sol.y(n, maturityIndices(1));
      end
```

```
    end
  end
%}
  figureH= figure(4); clf(figureH);
  position= get(figureH, 'Position');
  set(figureH, 'Position',[position(1) position(2) 800 400]);
 % Red -> unborn, Green -> born, Grey - mature
  colormap([1 0 0; 0 1 0; 0.5 0.5 0.5]);
 % Find maximum values across all cells for all time beyond start time
  max1p= max(1, max(max(sol.y(sol.params.H7TotalIndices(yStartIndex:yEndIndex), ...
tStartIndex:tEndIndex))));
  max1m= max(1, max(max(sol.y(sol.params.h7Indices(yStartIndex:yEndIndex), ...
tStartIndex:tEndIndex))));
  switch sol.params.numGenes
    case 1
      axisH1p= subplot(1,2,1); % Her1 Protein
      axisH1m= subplot(1,2,2); % Her1 mRNA
    case 2
      axisH1p= subplot(2,2,1); % Her1 Protein
      axisH1m= subplot(2,2,2); % Her1 mRNA
      axisH2p= subplot(2,2,3); % Delta Protein
      axisH2m= subplot(2,2,4); % Delta mRNA
      max2p= max(1, max(max(sol.y(sol.params.DIndices(yStartIndex:yEndIndex), ...
tStartIndex:tEndIndex))));
      max2m= max(1, max(max(sol.y(sol.params.dIndices(yStartIndex:yEndIndex),...
tStartIndex:tEndIndex))));
    otherwise
      error([ 'sol.params.numGenes == ' num2str(sol.params.numGenes) ...
' not supported.' ]);
  end

 % Stop button setup
  figurePosition= get(figureH, 'Position');
  buttonPosition= get(0,'DefaultUicontrolPosition');
  buttonPosition(1:2)= [figurePosition(3)/2 figurePosition(4)] - 25;
  buttonH= uicontrol('Style','pushbutton', 'String','Stop', ...
'Position',buttonPosition, 'Callback',@StopButtonCallback, 'Tag','stop');
  ud.stop= 0;
  set(figureH, 'UserData',ud);
%{
  MakeQTMovie('start', 'movie.mov');
  MakeQTMovie('quality', 0.75);
  MakeQTMovie('framerate', 6);
%}
  for frame= 1 : length(tGrid)
```

```
   % Update button position and check for click
    figurePosition= get(figureH, 'Position');
    buttonPosition(1:2)= [figurePosition(3)/2 figurePosition(4)] - 25;
    set(buttonH, 'Position',buttonPosition);
    ud= get(figureH,'UserData');
    if ud.stop
      break;
    end

   % Calculate solution on time grid
    y= deval(sol, tGrid(frame));

  % Calculate (un)born switches
    s= ones(sol.params.cellArraySize(1), 1); % Default: 1 <=> Unborn color index
    s(sol.params.birthTimes(yStartIndex:yEndIndex) <= tGrid(frame))= 2; % 2 <=> Born ...
color index
%    s(sol.params.somitesInPSM*sol.params.tailbudPeriod + ...
%sol.params.birthTimes <= tGrid(frame))= 3; % 3 <=> Mature color index

% WIP: Do stacked bar plots to show dimer distributions (single bar bug in Matlab)
%    [H13, H7, H13H13, H7H7, H7H13]= calcConcs(...);
%    barH1p= bar(axisH1p, 1:sol.params.numCells, ...
%[H1H13, H1H1, y(1:sol.params.equationsPerCell:end) - H113 - H11], 'stacked');
%    axesH1p(1)= axisH1p;
%    axesH1p(2)= axes('Position',get(axesH1p(1),'Position'), ...
%'XAxisLocation','top', 'YAxisLocation','right');
%    plot(axesH1p(2), 1:sol.params.numCells, H13);

    gradientFuncH= str2func(sol.params.gradientFunc);

   % Her7 & Her13 plots
    [axesH1p, barH1p, seriesH1p]= ...
plotyy(axisH1p, 1:sol.params.numCells, ...
y(sol.params.H7TotalIndices(yStartIndex:yEndIndex)), ...
1:sol.params.cellArraySize(1), gradientFuncH(tGrid(frame) - ...
sol.params.birthTimes(yStartIndex:yEndIndex), sol.params.H13TotalMax, ...
sol.params.H13TotalMin, sol.params.H13TotalHalfLife), 'bar', 'plot');
    set(seriesH1p, 'LineWidth',2);
    childrenH1p= get(barH1p, 'children');
    set(childrenH1p, 'facevertexcdata', s); % Set color of bars
    set(childrenH1p, 'CDataMapping','direct');
    ylim(axesH1p(1), [0, 1.1*max1p]);
    ylabel(axesH1p(1), '$\mathbf{\widehat{C}}$␣{\bf␣protein␣(copy␣\#)}', ...
'Interpreter','latex', 'FontWeight','demi', 'FontSize',12);
    set(axesH1p(1), 'YTickMode', 'auto');
    ylim(axesH1p(2), [0, 1.1*max(1, max(sol.params.H13TotalMax(:)))]);
```

```
    ylabel(axesH1p(2), '$\mathbf{\widehat{G}}$ {\bf protein (copy \#)}', ...
'Interpreter','latex', 'FontWeight','demi', 'FontSize',12);
    set(axesH1p(2), 'YTickMode', 'auto');
    xlim(axesH1p(1), [0, sol.params.cellArraySize(1)+1]);
    xlim(axesH1p(2), [0, sol.params.cellArraySize(1)+1]);
    set(axesH1p, 'FontWeight','demi');
    title(axisH1p, ['time= ' num2str(tGrid(frame), '%1.1f') ' min'], ...
'FontWeight','demi', 'FontSize',14);
    position= get(axisH1p, 'Position');
    position= [0.1 position(2) 0.3 0.325];
    set(axisH1p, 'Position',position);

    [axesH1m, barH1m, seriesH1m]= ...
plotyy(axisH1m, 1:sol.params.cellArraySize(1), ...
y(sol.params.h7Indices(yStartIndex:yEndIndex)), ...
1:sol.params.cellArraySize(1), gradientFuncH(tGrid(frame) - ...
sol.params.birthTimes(yStartIndex:yEndIndex), sol.params.H13TotalMax, ...
sol.params.H13TotalMin, sol.params.H13TotalHalfLife), 'bar', 'plot');
%    [axesH1m, barH1m, seriesH1m]= ...
%plotyy(axisH1m, 1:sol.params.numCells, y(3:sol.params.equationsPerCell:end)/6.022/(4/3*...
pi*(4^3-(2.5)^3))*1e4, ...
%1:sol.params.numCells, y(1:sol.params.equationsPerCell:end)/6.022/(4/3*pi*(2.5)^3)*1e4, ...
'bar', 'plot');
    set(seriesH1m, 'LineWidth',2);
    childrenH1m= get(barH1m, 'children');
    set(childrenH1m, 'facevertexcdata', s); % Set color of bars
    set(childrenH1m, 'CDataMapping','direct');
%    ylim(axesH1m(1), [0, 1.1*max1m/6.022/(4/3*pi*(4^3-(2.5)^3))*1e4]);
    ylim(axesH1m(1), [0, 1.1*max1m]);
%    ylabel(axesH1m(1), 'Cytosolic her7 mRNA conc. (pM)', 'FontWeight','demi', 'FontSize...
',12);
    ylabel(axesH1m(1), '$\mathbf{c}$ {\bf mRNA (copy \#)}', ...
'Interpreter','latex', 'FontWeight','demi', 'FontSize',12);
    set(axesH1m(1), 'YTickMode', 'auto');
%    ylim(axesH1m(2), [0, 1.1*max(1, sol.params.H13TotalMax/6.022/(4/3*pi*((2.5)^3))*1e4)...
]);
    ylim(axesH1m(2), [0, 1.1*max(1, max(sol.params.H13TotalMax(:)))]);
%    ylabel(axesH1m(2), 'Total nuclear Her13.2 conc. (pM)', 'FontWeight','demi', '...
FontSize',12);
    ylabel(axesH1m(2), '$\mathbf{\widehat{G}}$ {\bf protein (copy \#)}', ...
'Interpreter','latex', 'FontWeight','demi', 'FontSize',12);
    set(axesH1m(2), 'YTickMode', 'auto');
    xlim(axesH1m(1), [0, sol.params.cellArraySize(1)+1]);
    xlim(axesH1m(2), [0, sol.params.cellArraySize(1)+1]);
    set(axesH1m(1), 'XTickMode', 'auto');
    set(axesH1m(2), 'XTickMode', 'auto');
```

```
    set(axesH1m, 'FontWeight','demi');
    title(axisH1m, ['time=␣' num2str(tGrid(frame), '%1.1f') '␣min'], ...
'FontWeight','demi', 'FontSize',14);
%    title(axisH1m, ['Two Binding-Site Model; t= ' num2str(tGrid(frame), '%1.1f') ' min...
'], ...
%'FontWeight','demi', 'FontSize',14);
    position= get(axisH1m, 'Position');
    position= [0.6 position(2) 0.3 0.325];
    set(axisH1m, 'Position',position);

    if sol.params.numGenes == 2 % Delta & Her13 plots
      [axesH2p, barH2p, seriesH2p]= ...
plotyy(axisH2p, 1:sol.params.cellArraySize(1), ...
y(sol.params.DIndices(yStartIndex:yEndIndex)), ...
1:sol.params.cellArraySize(1), gradientFuncH(tGrid(frame) - ...
sol.params.birthTimes(yStartIndex:yEndIndex), sol.params.H13TotalMax, ...
sol.params.H13TotalMin, sol.params.H13TotalHalfLife), 'bar', 'plot');
      set(seriesH2p, 'LineWidth',2);
      childrenH2p= get(barH2p, 'children');
      set(childrenH2p, 'facevertexcdata', s); % Set color of bars
      set(childrenH2p, 'CDataMapping','direct');
      ylim(axesH2p(1), [0, 1.1*max2p]);
      ylabel(axesH2p(1), '$\mathbf{S}$␣{\bf␣protein␣(copy␣\#)}', ...
'Interpreter','latex', 'FontWeight','demi', 'FontSize',12);
      set(axesH2p(1), 'YTickMode', 'auto');
      ylim(axesH2p(2), [0, 1.1*max(1, max(sol.params.H13TotalMax(:)))]);
      ylabel(axesH2p(2), '$\mathbf{\widehat{G}}$␣{\bf␣protein␣(copy␣\#)}', ...
'Interpreter','latex', 'FontWeight','demi', 'FontSize',12);
      set(axesH2p(2), 'YTickMode', 'auto');
      xlim(axesH2p(1), [0, sol.params.cellArraySize(1)+1]);
      xlim(axesH2p(2), [0, sol.params.cellArraySize(1)+1]);
      xlabel(axesH2p(1), 'Cell␣#␣(1␣is␣anterior)', ...
'FontWeight','demi', 'FontSize',12);
      set(axesH2p, 'FontWeight','demi');
      position= get(axisH2p, 'Position');
      position= [0.1 position(2) 0.3 0.325];
      set(axisH2p, 'Position',position);

      [axesH2m, barH2m, seriesH2m]= ...
plotyy(axisH2m, 1:sol.params.cellArraySize(1), ...
y(sol.params.dIndices(yStartIndex:yEndIndex)), ...
1:sol.params.cellArraySize(1), gradientFuncH(tGrid(frame) - ...
sol.params.birthTimes(yStartIndex:yEndIndex), sol.params.H13TotalMax, ...
sol.params.H13TotalMin, sol.params.H13TotalHalfLife), 'bar', 'plot');
      set(seriesH2m, 'LineWidth',2);
      childrenH2m= get(barH2m, 'children');
```

```
      set(childrenH2m, 'facevertexcdata', s); % Set color of bars
      set(childrenH2m, 'CDataMapping','direct');
      ylim(axesH2m(1), [0, 1.1*max2m]);
      ylabel(axesH2m(1), '$\mathbf{s}$␣{\bf␣mRNA␣(copy␣\#)}', ...
'Interpreter','latex', 'FontWeight','demi', 'FontSize',12);
      set(axesH2m(1), 'YTickMode', 'auto');
      ylim(axesH2m(2), [0, 1.1*max(1, max(sol.params.H13TotalMax(:)))]);
      ylabel(axesH2m(2), '$\mathbf{\widehat{G}}$␣{\bf␣Protein␣(copy␣\#)}', ...
'Interpreter','latex', 'FontWeight','demi', 'FontSize',12);
      set(axesH2m(2), 'YTickMode', 'auto');
      xlim(axesH2m(1), [0, sol.params.cellArraySize(1)+1]);
      xlim(axesH2m(2), [0, sol.params.cellArraySize(1)+1]);
      xlabel(axesH2m(1), 'Cell␣#␣(1␣is␣anterior)', ...
'FontWeight','demi', 'FontSize',12);
      set(axesH2m, 'FontWeight','demi');
      position= get(axisH2m, 'Position');
      position= [0.6 position(2) 0.3 0.325];
      set(axisH2m, 'Position',position);
    else
      xlabel(axesH1p(1), 'Cell␣#␣(1␣is␣anterior)', ...
'FontWeight','demi', 'FontSize',12);
      xlabel(axesH1m(1), 'Cell␣#␣(1␣is␣anterior)', ...
'FontWeight','demi', 'FontSize',12);
    end

    animation(frame)= getframe;
%     MakeQTMovie('addfigure');
  end

%  MakeQTMovie('finish');

 % Nested Functions

  function StopButtonCallback(src, eventdata)
    ud= get(gcbf, 'UserData');
    ud.stop= 1;
    set(gcbf, 'UserData',ud);
  end  % StopButtonCallback

end
```

1.3.3. `animateContour.m`. A simple animation function for 2D rectangular arrays of cells.

```
function animation= animateContour(sol, tStartPlot, tEndPlot, tDelta)
%
% Usage:
%  animation= animateContour(sol, tStartPlot, tEndPlot, tDelta)
```

```
%
% A QT movie can be recorded by uncommenting the calls to MakeQTMovie()

  tStartPlot= max([sol.x(1), tStartPlot]); % Domain check
  tStartIndex= find((sol.x(1:end-1) <= tStartPlot) & (sol.x(2:end) > tStartPlot));
  tEndPlot= min([sol.x(end), tEndPlot]); % Domain check
  tEndIndex= find((sol.x(1:end-1) < tEndPlot) & (sol.x(2:end) >= tEndPlot));
  tGrid= tStartPlot : tDelta : tEndPlot; % Assumes tStartPlot <= tEndPlot

  figureH= figure(4); clf(figureH);
    colormap(flipdim(colormap('gray'),1)); % Applies to all axes in figure

 % Find maximum values across all cells for all time beyond start time
  max1p= max(1, max(max(sol.y(sol.params.H7TotalIndices,tStartIndex:tEndIndex))));
  max1m= max(1, max(max(sol.y(sol.params.h7Indices,tStartIndex:tEndIndex))));
  switch sol.params.numGenes
    case 1
      axisH1p= subplot(1,2,1); % Her1 protein
      axisH1m= subplot(1,2,2); % Her1 mRNA
    case 2
      axisH1p= subplot(2,2,1); % Her1 protein
      axisH1m= subplot(2,2,2); % Her1 mRNA
      axisH2p= subplot(2,2,3); % DeltaC protein
      axisH2m= subplot(2,2,4); % DeltaC mRNA
     % Find additional maximum values
      max2p= max(1, max(max(sol.y(sol.params.DIndices,tStartIndex:tEndIndex))));
      max2m= max(1, max(max(sol.y(sol.params.dIndices,tStartIndex:tEndIndex))));
    otherwise
      error(['sol.params.numGenes␣==␣' num2str(sol.params.numGenes) ...
'␣not␣supported.']);
  end

 % Stop button setup
  figurePosition= get(figureH, 'Position');
  buttonPosition= get(0,'DefaultUicontrolPosition');
  buttonPosition(1:2)= [figurePosition(3)/2 figurePosition(4) - 25];
  buttonH= uicontrol('Style','pushbutton', 'String','Stop', ...
'Position',buttonPosition, 'Callback',@StopButtonCallback, 'Tag','stop');
  ud.stop= 0;
  set(figureH, 'UserData',ud);
%{
  MakeQTMovie('start', 'movie.mov');
  MakeQTMovie('quality', 0.75);
  MakeQTMovie('framerate', 6);
%}
  for frame= 1 : length(tGrid)
```

```
  % Update button position and check for click
   figurePosition= get(figureH, 'Position');
   buttonPosition(1:2)= [figurePosition(3)/2 figurePosition(4) - 25];
   set(buttonH, 'Position',buttonPosition);
   ud= get(figureH,'UserData');
   if ud.stop
     break;
   end

  % Calculate solution on time grid
   y= deval(sol, tGrid(frame));

  % Her7 & Her13 plots
    imagesc(reshape(y(sol.params.H7TotalIndices)/max1p, ...
sol.params.cellArraySize(1), sol.params.cellArraySize(2))', 'Parent',axisH1p);
    xlabel(axisH1p, 'Axial␣Cell␣#␣(1␣is␣anterior)', 'FontWeight','demi', ...
'FontSize',12);
    xlim(axisH1p, [0 sol.params.cellArraySize(1)+1]);
    ylabel(axisH1p, 'Lateral␣Cell␣#', 'FontWeight','demi', 'FontSize',12);
    set(axisH1p, 'YTick',0:sol.params.cellArraySize(2))
    title(axisH1p, ['\bf␣$\mathbf{\widehat{C}}$␣\textsf{Protein,␣time=␣' ...
num2str(tGrid(frame), '%1.1f') '␣min}'], 'Interpreter','latex', ...
'FontWeight','demi', 'FontSize',16);
    set(axisH1p, 'Clim', [0 1]);

    imagesc(reshape(y(sol.params.h7Indices)/max1m, ...
sol.params.cellArraySize(1), sol.params.cellArraySize(2))', 'Parent',axisH1m);
    xlim(axisH1m, [0 sol.params.cellArraySize(1)+1]);
    xlabel(axisH1m, 'Axial␣Cell␣#␣(1␣is␣anterior)', 'FontWeight','demi', 'FontSize',12)
    ylabel(axisH1m, 'Lateral␣Cell␣#', 'FontWeight','demi', 'FontSize',12)
    set(axisH1m, 'YTick',0:sol.params.cellArraySize(2))
    title(axisH1m, ['\bf␣$\mathbf{c}$␣\textsf{mRNA,␣time=␣' ...
num2str(tGrid(frame), '%1.1f') '␣min}'], 'Interpreter','latex', ...
'FontWeight','demi', 'FontSize',16);
    set(axisH1m, 'Clim', [0 1]);

    if sol.params.numGenes == 2 % Delta & Her13 plots
      imagesc(reshape(y(sol.params.DIndices)/max2p, ...
sol.params.cellArraySize(1), sol.params.cellArraySize(2))', 'Parent',axisH2p);
      xlim(axisH2p, [0 sol.params.cellArraySize(1)+1]);
      xlabel(axisH2p, 'Axial␣Cell␣#␣(1␣is␣anterior)', 'FontWeight','demi', 'FontSize',12)
      ylabel(axisH2p, 'Lateral␣Cell␣#', 'FontWeight','demi', 'FontSize',12)
      set(axisH2p, 'YTick',0:sol.params.cellArraySize(2))
      title(axisH2p, ['\bf␣$\mathbf{S}$␣\textsf{Protein,␣time=␣' ...
num2str(tGrid(frame), '%1.1f') '␣min}'], 'Interpreter','latex', ...
```

```
'FontWeight','demi', 'FontSize',16);
      set(axisH2p, 'Clim', [0 1]);

      imagesc(reshape(y(sol.params.dIndices)/max2m, ...
sol.params.cellArraySize(1), sol.params.cellArraySize(2))', 'Parent',axisH2m);
      xlim(axisH2m, [0 sol.params.cellArraySize(1)+1]);
      xlabel(axisH2m, 'Axial␣Cell␣#␣(1␣is␣anterior)', 'FontWeight','demi', 'FontSize',12)
      ylabel(axisH2m, 'Lateral␣Cell␣#', 'FontWeight','demi', 'FontSize',12)
      set(axisH2m, 'YTick',0:sol.params.cellArraySize(2))
      title(axisH2m, ['\bf␣$\mathbf{s}$␣\textsf{mRNA,␣time=␣' ...
num2str(tGrid(frame), '%1.1f') '␣min}'], 'Interpreter','latex', ...
'FontWeight','demi', 'FontSize',16);
      set(axisH2m, 'Clim', [0 1]);
    end

    animation(frame)= getframe;
%     MakeQTMovie('addfigure');
  end

%  MakeQTMovie('finish');

 % Nested Functions

  function StopButtonCallback(src, eventdata)
    ud= get(gcbf, 'UserData');
    ud.stop= 1;
    set(gcbf, 'UserData',ud);
  end  % StopButtonCallback

end
```

1.3.4. `MakeQTMovie.m`. A third-party function for converting animations into QuickTime movies.

```
function MakeQTMovie(cmd,arg, arg2)
% function MakeQTMovie(cmd, arg, arg2)
% Create a QuickTime movie from a bunch of figures (and an optional sound).
%
% Syntax: MakeQTMovie cmd [arg]
% The following commands are supported:
%       addfigure - Add snapshot of current figure to movie
%       addaxes - Add snapshot of current axes to movie
%       addmatrix data - Add a matrix to movie (convert to jpeg with imwrite)
%       addmatrixsc data - Add a matrix to movie (convert to jpeg with imwrite)
%               (automatically scales image data)
%       addsound data [sr] - Add sound to movie (only monaural for now)
%               (third argument is the sound's sample rate.)
%       cleanup - Remove the temporary files
```

```
%       demo - Create a demonstration movie
%       finish - Finish movie, write out QT file
%       framerate fps - Set movies frame rate [Default is 10 fps]
%       quality # - Set JPEG quality (between 0 and 1)
%       size [# #] - Set plot size to [width height]
%       start filename - Start creating a movie with this name
% The start command must be called first to provide a movie name.
% The finish command must be called last to write out the movie
% data. All other commands can be called in any order.  Only one
% movie can be created at a time.
%
% This code is published as Interval Technical Report #1999-066
% The latest copy can be found at
%       http://web.interval.com/papers/1999-066/
% (c) Copyright Malcolm Slaney, Interval Research, March 1999.

% This is experimental software and is being provided to Licensee
% 'AS IS.'  Although the software has been tested on Macintosh, SGI,
% Linux, and Windows machines, Interval makes no warranties relating
% to the software's performance on these or any other platforms.
%
% Disclaimer
% THIS SOFTWARE IS BEING PROVIDED TO YOU 'AS IS.'  INTERVAL MAKES
% NO EXPRESS, IMPLIED OR STATUTORY WARRANTY OF ANY KIND FOR THE
% SOFTWARE INCLUDING, BUT NOT LIMITED TO, ANY WARRANTY OF
% PERFORMANCE, MERCHANTABILITY OR FITNESS FOR A PARTICULAR PURPOSE.
% IN NO EVENT WILL INTERVAL BE LIABLE TO LICENSEE OR ANY THIRD
% PARTY FOR ANY DAMAGES, INCLUDING LOST PROFITS OR OTHER INCIDENTAL
% OR CONSEQUENTIAL DAMAGES, EVEN IF INTERVAL HAS BEEN ADVISED OF
% THE POSSIBLITY THEREOF.
%
%   This software program is owned by Interval Research
% Corporation, but may be used, reproduced, modified and
% distributed by Licensee.  Licensee agrees that any copies of the
% software program will contain the same proprietary notices and
% warranty disclaimers which appear in this software program.

% This program uses the Matlab imwrite routine to convert each image
% frame into JPEG.  After first reserving 8 bytes for a header that points
% to the movie description, all the compressed images and the sound are
% added to the movie file.  When the 'finish' method is called then the
% first 8 bytes of the header are rewritten to indicate the size of the
% movie data, and then the movie header ('moov structure') is written
% to the output file.
%
% This routine creates files according to the QuickTime file format as
```

```
% described in the appendix of
%       "Quicktime (Inside MacIntosh)," Apple Computer Incorporated,
%       Addison-Wesley Pub Co; ISBN: 0201622017, April 1993.
% I appreciate help that I received from Lee Fyock (MathWorks) and Aaron
% Hertzmann (Interval) in debugging and testing this work.

% Changes:
% July 5, 1999 - Removed stss atom since it upset PC version of QuickTime
% November 11, 1999 - Fixed quality bug in addmatrix.  Added addmatrixsc.
% March 7, 2000 - by Jordan Rosenthal (jr@ece.gatech.edu), Added truecolor
%    capability when running in Matlab 5.3 changed some help comments, fixed
%    some bugs, vectorized some code.
% April 7, 2000 - by Malcolm.  Cleaned up axis/figure code and fixed(?) SGI
%    playback problems.  Added user data atom to give version information.
%    Fixed sound format problems.
% April 10, 2000 - by Malcolm. Fixed problem with SGI (at least) and B&W
%    addmatrix.

if nargin < 1
        fprintf('Syntax: MakeQTMovie cmd [arg]\n')
        fprintf('The following commands are supported:\n');
        fprintf('        addfigure - Add snapshot of current figure to movie\n')
        fprintf('        addaxes - Add snapshot of current axes to movie\n')
        fprintf('        addmatrix data - Add a matrix to movie ');
                        fprintf('(convert to jpeg)\n')
        fprintf('        addmatrixsc data - Add a matrix to movie ');
                        fprintf('(scale and convert to jpeg)\n')
        fprintf('        addsound data - Add sound samples ');
                        fprintf('(with optional rate)\n')
        fprintf('        demo - Show this program in action\n');
        fprintf('        finish - Finish movie, write out QT file\n');
        fprintf('        framerate # - Set movie frame rate ');
                        fprintf('(default is 10fps)\n');
        fprintf('        quality # - Set JPEG quality (between 0 and 1)\n');
        fprintf('        size [# #] - Set plot size to [width height]\n');
        fprintf('        start filename - Start making a movie with ');
                        fprintf('this name\n');
        return;
end

global MakeQTMovieStatus
MakeDefaultQTMovieStatus;                % Needed first time, ignored otherwise

switch lower(cmd)
case {'addframe','addplot','addfigure','addaxes'}
        switch lower(cmd)
```

```
        case {'addframe','addfigure'}
                hObj = gcf;             % Add the entire figure (with all axes)
        otherwise
                hObj = gca;             % Add what's inside the current axis
        end
        frame = getframe(hObj);
        [I,map] = frame2im(frame);
        if ImageSizeChanged(size(I)) > 0
                return;
        end
        if isempty(map)
                                        % RGB image
                imwrite(I,MakeQTMovieStatus.imageTmp, 'jpg', 'Quality', ...
                 MakeQTMovieStatus.spatialQual*100);
        else
                                        % Indexed image
                writejpg_map(MakeQTMovieStatus.imageTmp, I, map);
        end
        [pos, len] = AddFileToMovie;
        n = MakeQTMovieStatus.frameNumber + 1;
        MakeQTMovieStatus.frameNumber = n;
        MakeQTMovieStatus.frameStarts(n) = pos;
        MakeQTMovieStatus.frameLengths(n) = len;

%% Allow images to be added by doing:
%%      MakeQTMovie('addimage', '/path/to/file.jpg');
%% This case adapted from addmatrix.  Thanks to
%% Stephen Eglen <stephen@cogsci.ed.ac.uk> for this idea.
case 'addimage'
        if nargin < 2
                fprintf('MakeQTMovie error: Need to specify a filename with ');
                fprintf('the image command.\n');
                return;
        end

        %% Check to see that the image is the correct size.  Do
        %% this by reading in the image and then checking its size.
        %% tim - temporary image.
        tim = imread(arg); tim_size = size(tim);

        fprintf('Image %s size %d %d\n', arg, tim_size(1), tim_size(2));
        if ImageSizeChanged(tim_size) > 0
                return;
        end
        [pos, len] = AddFileToMovie(arg);
        n = MakeQTMovieStatus.frameNumber + 1;
```

```
        MakeQTMovieStatus.frameNumber = n;
        MakeQTMovieStatus.frameStarts(n) = pos;
        MakeQTMovieStatus.frameLengths(n) = len;

case 'addmatrix'
        if nargin < 2
                fprintf('MakeQTMovie error: Need to specify a matrix with ');
                fprintf('the addmatrix command.\n');
                return;
        end
        if ImageSizeChanged(size(arg)) > 0
                return;
        end
                                % Work around a bug, at least on the
                                % SGIs, which causes JPEGs to be
                                % written which can't be read with the
                                % SGI QT.  Turn the B&W image into a
                                % color matrix.
        if ndims(arg) < 3
                arg(:,:,2) = arg;
                arg(:,:,3) = arg(:,:,1);
        end
        imwrite(arg, MakeQTMovieStatus.imageTmp, 'jpg', 'Quality', ...
                MakeQTMovieStatus.spatialQual*100);
        [pos, len] = AddFileToMovie;
        n = MakeQTMovieStatus.frameNumber + 1;
        MakeQTMovieStatus.frameNumber = n;
        MakeQTMovieStatus.frameStarts(n) = pos;
        MakeQTMovieStatus.frameLengths(n) = len;

case 'addmatrixsc'
        if nargin < 2
                fprintf('MakeQTMovie error: Need to specify a matrix with ');
                fprintf('the addmatrix command.\n');
                return;
        end
        if ImageSizeChanged(size(arg)) > 0
                return;
        end
        arg = arg - min(min(arg));
        arg = arg / max(max(arg));
                                % Work around a bug, at least on the
                                % SGIs, which causes JPEGs to be
                                % written which can't be read with the
                                % SGI QT.  Turn the B&W image into a
                                % color matrix.
```

```
        if ndims(arg) < 3
                arg(:,:,2) = arg;
                arg(:,:,3) = arg(:,:,1);
        end
        imwrite(arg, MakeQTMovieStatus.imageTmp, 'jpg', 'Quality', ...
                MakeQTMovieStatus.spatialQual*100);
        [pos, len] = AddFileToMovie;
        n = MakeQTMovieStatus.frameNumber + 1;
        MakeQTMovieStatus.frameNumber = n;
        MakeQTMovieStatus.frameStarts(n) = pos;
        MakeQTMovieStatus.frameLengths(n) = len;

case 'addsound'
        if nargin < 2
                fprintf('MakeQTMovie␣error:␣Need␣to␣specify␣a␣sound␣array␣');
                fprintf('with␣the␣addsound␣command.\n');
                return;
        end
                                        % Do stereo someday???
        OpenMovieFile
        MakeQTMovieStatus.soundLength = length(arg);
        arg = round(arg/max(max(abs(arg)))*32765);
        negs = find(arg<0);
        arg(negs) = arg(negs) + 65536;

        sound = mb16(arg);
        MakeQTMovieStatus.soundStart = ftell(MakeQTMovieStatus.movieFp);
        MakeQTMovieStatus.soundLen = length(sound);
        fwrite(MakeQTMovieStatus.movieFp, sound, 'uchar');
        if nargin < 3
                arg2 = 22050;
        end
        MakeQTMovieStatus.soundRate = arg2;

case 'cleanup'
        if isstruct(MakeQTMovieStatus)
%     if isfield(MakeQTMovieStatus, 'movieFp')
                if ~isempty(MakeQTMovieStatus.movieFp)
                        fclose(MakeQTMovieStatus.movieFp);
                        MakeQTMovieStatus.movieFp = [];
       end
%     end
%     if isfield(MakeQTMovieStatus, 'imageTmp')
                if ~isempty(MakeQTMovieStatus.imageTmp) & ...
                   exist(MakeQTMovieStatus.imageTmp,'file') > 0
                        delete(MakeQTMovieStatus.imageTmp);
```

```
                                MakeQTMovieStatus.imageTmp = [];
      end
%                 end
        end
        MakeQTMovieStatus = [];

case 'debug'
        fprintf('Current␣Movie␣Data:\n');
        fprintf('␣␣␣␣%d␣frames␣at␣%d␣fps\n', MakeQTMovieStatus.frameNumber, ...
                                        MakeQTMovieStatus.frameRate);
        starts = MakeQTMovieStatus.frameStarts;
        if length(starts) > 10, starts = starts(1:10);, end;
        lens = MakeQTMovieStatus.frameLengths;
        if length(lens) > 10, lens = lens(1:10);, end;
        fprintf('␣␣␣␣␣␣␣␣␣␣Start:␣%6d␣␣␣␣␣␣Size:␣%6d\n', [starts; lens]);
        fprintf('␣␣␣␣Movie␣Image␣Size:␣%dx%d\n', ...
                MakeQTMovieStatus.imageSize(2), ...);
                MakeQTMovieStatus.imageSize(1));
        if length(MakeQTMovieStatus.soundStart) > 0
                fprintf('␣␣␣␣Sound:␣%d␣samples␣at␣%d␣Hz␣sampling␣rate␣', ...
                        MakeQTMovieStatus.soundLength, ...
                        MakeQTMovieStatus.soundRate);
                fprintf('at␣%d.\n', MakeQTMovieStatus.soundStart);
        else
                fprintf('␣␣␣␣Sound:␣No␣sound␣track\n');
        end
        fprintf('␣␣␣␣Temporary␣files␣for␣images:␣%s\n', ...
                MakeQTMovieStatus.imageTmp);
        fprintf('␣␣␣␣Final␣movie␣name:␣%s\n', MakeQTMovieStatus.movieName);
        fprintf('␣␣␣␣Compression␣Quality:␣%g\n', ...
                MakeQTMovieStatus.spatialQual);


case 'demo'
        clf
        fps = 10;
        movieLength = 10;
        sr = 22050;
        fn = 'test.mov';
        fprintf('Creating␣the␣movie␣%s.\n', fn);
        MakeQTMovie('start',fn);
        MakeQTMovie('size', [160 120]);
        MakeQTMovie('quality', 1.0);
        theSound = [];
        for i=1:movieLength
                plot(sin((1:100)/4+i));
```

```
            MakeQTMovie('addaxes');
            theSound = [theSound sin(440/sr*2*pi*(2^(i/12))*(1:sr/fps))];
        end
        MakeQTMovie('framerate', fps);
        MakeQTMovie('addsound', theSound, sr);
        MakeQTMovie('finish');

case {'finish','close'}
        AddQTHeader;
        MakeQTMovie('cleanup')                  % Remove temporary files

case 'framerate'
        if nargin < 2
                fprintf('MakeQTMovie␣error:␣Need␣to␣specify␣the␣');
                fprintf('frames/second␣with␣the␣framerate␣command.\n');
                return;
        end
        MakeQTMovieStatus.frameRate = arg;

case 'help'
        MakeQTMovie                             % To get help message.

case 'size'
                                                % Size is off by one on the
                                                % Mac.
        if nargin < 2
                fprintf('MakeQTMovie␣error:␣Need␣to␣specify␣a␣vector␣with␣');
                fprintf('the␣size␣command.\n');
                return;
        end
        if length(arg) ~= 2
                error('MakeQTMovie:␣Error,␣must␣supply␣2␣element␣size.');
        end
        oldUnits = get(gcf,'units');
        set(gcf,'units','pixels');
        cursize = get(gcf, 'position');
        cursize(3) = arg(1);
        cursize(4) = arg(2);
        set(gcf, 'position', cursize);
        set(gcf,'units',oldUnits);

case 'start'
        if nargin < 2
                fprintf('MakeQTMovie␣error:␣Need␣to␣specify␣a␣file␣name␣');
                fprintf('with␣start␣command.\n');
                return;
```

```
        end
        MakeQTMovie('cleanup');
        MakeDefaultQTMovieStatus;
        MakeQTMovieStatus.movieName = arg;

case 'test'
        clf
        MakeQTMovieStatus = [];
        MakeQTMovie('start','test.mov');
        MakeQTMovie('size', [320 240]);
        MakeQTMovie('quality', 1.0);
        subplot(2,2,1);
        for i=1:10
                plot(sin((1:100)/4+i));
                MakeQTMovie('addfigure');
        end
        MakeQTMovie('framerate', 10);
        MakeQTMovie('addsound', sin(1:5000), 22050);
        MakeQTMovie('debug');
        MakeQTMovie('finish');

case 'quality'
        if nargin < 2
                fprintf('MakeQTMovie error: Need to specify a quality ');
                fprintf('(between 0-1) with the quality command.\n');
                return;
        end
        MakeQTMovieStatus.spatialQual = arg;

otherwise
        fprintf('MakeQTMovie: Unknown method %s.\n', cmd);
end

%%%%%%%%%%%%%%%  MakeDefaultQTMovieStatus %%%%%%%%%%%%%%%%%
% Make the default movie status structure.
function MakeDefaultQTMovieStatus
global MakeQTMovieStatus
if isempty(MakeQTMovieStatus)
   MakeQTMovieStatus = struct(...
      'frameRate', 10, ...       % frames per second
      'frameStarts', [], ...   % Starting byte position
      'frameLengths', [], ...
      'timeScale', 10, ...       % How much faster does time run?
      'soundRate', 22050, ... % Sound Sample Rate
      'soundStart', [], ...      % Starting byte position
      'soundLength', 0, ...
```

```
        'soundChannels', 1, ...    % Number of channels
        'frameNumber', 0, ...
        'movieFp', [], ...                 % File pointer
        'imageTmp', tempname, ...
        'movieName', 'output.mov', ...
        'imageSize', [0 0], ...
        'trackNumber', 0, ...
        'timeScaleExpansion', 100, ...
        'spatialQual', 1.0);       % Between 0.0 and 1.0
end


%%%%%%%%%%%%%%  ImageSizeChanged %%%%%%%%%%%%%%%%
% Check to see if the image size has changed.  This m-file can't
% deal with that, so we'll return an error.
function err = ImageSizeChanged(newsize)
global MakeQTMovieStatus

newsize = newsize(1:2);                    % Don't care about RGB info, if present
oldsize= MakeQTMovieStatus.imageSize;
err = 0;

if sum(oldsize) == 0
        MakeQTMovieStatus.imageSize = newsize;
else
        if sum(newsize ~= oldsize) > 0
                fprintf('MakeQTMovie Error: New image size');
                fprintf('(%dx%d) doesn''t match old size (%dx%d)\n', ...
                        newsize(1), newsize(2), oldsize(1), oldsize(2));
                fprintf('   Can''t add this image to the movie.\n');
                err = 1;
        end
end

%%%%%%%%%%%%%%  AddFileToMovie %%%%%%%%%%%%%%%%
% OK, we've saved out an image file.  Now add it to the end of the movie
% file we are creating.
% We'll copy the JPEG file in 16kbyte chunks to the end of the movie file.
% Keep track of the start and end byte position in the file so we can put
% the right information into the QT header.
function [pos, len] = AddFileToMovie(imageTmp)
global MakeQTMovieStatus
OpenMovieFile
if nargin < 1
        imageTmp = MakeQTMovieStatus.imageTmp;
end
```

```
fp = fopen(imageTmp, 'rb');
if fp < 0
        error('Could␣not␣reopen␣QT␣image␣temporary␣file.');
end

len = 0;
pos = ftell(MakeQTMovieStatus.movieFp);
while 1
        data = fread(fp, 1024*16, 'uchar');
        if isempty(data)
                break;
        end
        cnt = fwrite(MakeQTMovieStatus.movieFp, data, 'uchar');
        len = len + cnt;
end
fclose(fp);

%%%%%%%%%%%%  AddQTHeader %%%%%%%%%%%%%%
% Go back and write the atom information that allows
% QuickTime to skip the image and sound data and find
% its movie description information.
function AddQTHeader()
global MakeQTMovieStatus

pos = ftell(MakeQTMovieStatus.movieFp);
header = moov_atom;
cnt = fwrite(MakeQTMovieStatus.movieFp, header, 'uchar');
fseek(MakeQTMovieStatus.movieFp, 0, -1);
cnt = fwrite(MakeQTMovieStatus.movieFp, mb32(pos), 'uchar');
fclose(MakeQTMovieStatus.movieFp);
MakeQTMovieStatus.movieFp = [];

%%%%%%%%%%%%  OpenMovieFile %%%%%%%%%%%%%%
% Open a new movie file.  Write out the initial QT header.  We'll fill in
% the correct length later.
function OpenMovieFile
global MakeQTMovieStatus
if isempty(MakeQTMovieStatus.movieFp)
        fp = fopen(MakeQTMovieStatus.movieName, 'wb');
        if fp < 0
                error('Could␣not␣open␣QT␣movie␣output␣file.');
        end
        MakeQTMovieStatus.movieFp = fp;
        cnt = fwrite(fp, [mb32(0) mbstring('mdat')], 'uchar');
end
```

```
%%%%%%%%%%%%%  writejpg_map %%%%%%%%%%%%%%%
% Like the imwrite routine, but first pass the image data through the indicated
% RGB map.
function writejpg_map(name,I,map)
global MakeQTMovieStatus

[y,x] = size(I);

% Force values to be valid indexes.  This fixes a bug that occasionally
% occurs in frame2im in Matlab 5.2 which incorrectly produces values of I
% equal to zero.
I = max(1,min(I,size(map,1)));

rgb = zeros(y, x, 3);
t = zeros(y,x);
t(:) = map(I(:),1)*255; rgb(:,:,1) = t;
t(:) = map(I(:),2)*255; rgb(:,:,2) = t;
t(:) = map(I(:),3)*255; rgb(:,:,3) = t;

imwrite(uint8(rgb),name,'jpeg','Quality',MakeQTMovieStatus.spatialQual*100);

%%%%%%%%%%%%%  SetAtomSize %%%%%%%%%%%%%%%
% Fill in the size of the atom
function y=SetAtomSize(x)
y = x;
y(1:4) = mb32(length(x));

%%%%%%%%%%%%%  mb32 %%%%%%%%%%%%%%%
% Make a vector from a 32 bit integer
function y = mb32(x)
if size(x,1) > size(x,2)
        x = x';
end

y = [bitand(bitshift(x,-24),255); ...
     bitand(bitshift(x,-16),255); ...
     bitand(bitshift(x, -8),255); ...
     bitand(x,              255)];
y = y(:)';

%%%%%%%%%%%%%  mb16 %%%%%%%%%%%%%%%
% Make a vector from a 16 bit integer
function y = mb16(x)
if size(x,1) > size(x,2)
        x = x';
end
```

```
y = [bitand(bitshift(x, -8),255); ...
     bitand(x,               255)];
y = y(:)';

%%%%%%%%%%%%%  mb8 %%%%%%%%%%%%%%%
% Make a vector from a 8 bit integer
function y = mb8(x)
if size(x,1) > size(x,2)
        x = x';
end

y = [bitand(x,               255)];
y = y(:)';

%
% The following routines all create atoms necessary
% to describe a QuickTime Movie. The basic idea is to
% fill in the necessary data, all converted to 8 bit
% characters, then fix it up later with SetAtomSize so
% that it has the correct header.  (This is easier than
% counting by hand.)

%%%%%%%%%%%%%  mbstring %%%%%%%%%%%%%%%
% Make a vector from a character string
function y = mbstring(s)
y = double(s);


%%%%%%%%%%%%%  dinf_atom %%%%%%%%%%%%%%%
function y = dinf_atom()
y = SetAtomSize([mb32(0) mbstring('dinf') dref_atom]);

%%%%%%%%%%%%%  dref_atom %%%%%%%%%%%%%%%
function y = dref_atom()
y = SetAtomSize([mb32(0) mbstring('dref') mb32(0) mb32(1) ...
                mb32(12) mbstring('alis') mb32(1)]);

%%%%%%%%%%%%%  edts_atom %%%%%%%%%%%%%%%
function y = edts_atom(add_sound_p)
global MakeQTMovieStatus
fixed1 = bitshift(1,16);                        % Fixed point 1
if add_sound_p > 0
        duration = MakeQTMovieStatus.soundLength / ...
                   MakeQTMovieStatus.soundRate * ...
                   MakeQTMovieStatus.timeScale;
```

```
else
        duration = MakeQTMovieStatus.frameNumber / ...
                        MakeQTMovieStatus.frameRate * ...
                        MakeQTMovieStatus.timeScale;
end
duration = ceil(duration);

y = [mb32(0) ...                                    % Atom Size
     mbstring('edts') ...                           % Atom Name
     SetAtomSize([mb32(0) ...                       % Atom Size
                  mbstring('elst') ...              % Atom Name
                  mb32(0) ...                       % Version/Flags
                  mb32(1) ...                       % Number of entries
                  mb32(duration) ...                % Length of this track
                  mb32(0) ...                       % Time
                  mb32(fixed1)])];                  % Rate
y = SetAtomSize(y);

%%%%%%%%%%%%%%  hdlr_atom %%%%%%%%%%%%%%%%
function y = hdlr_atom(component_type, sub_type)
if strcmp(sub_type, 'vide')
        type_string = 'Apple Video Media Handler';
elseif strcmp(sub_type, 'alis')
        type_string = 'Apple Alias Data Handler';
elseif strcmp(sub_type, 'soun')
        type_string = 'Apple Sound Media Handler';
end

y = [mb32(0) ...                                    % Atom Size
     mbstring('hdlr') ...                           % Atom Name
     mb32(0) ...                                    % Version and Flags
     mbstring(component_type) ...                   % Component Name
     mbstring(sub_type) ...                         % Sub Type Name
     mbstring('appl') ...                           % Component manufacturer
     mb32(0) ...                                    % Component flags
     mb32(0) ...                                    % Component flag mask
     mb8(length(type_string)) ...                   % Type Name byte count
     mbstring(type_string)];                        % Type Name
y = SetAtomSize(y);

%%%%%%%%%%%%%%  mdhd_atom %%%%%%%%%%%%%%%%
function y = mdhd_atom(add_sound_p)
global MakeQTMovieStatus

if add_sound_p
        data = [mb32(MakeQTMovieStatus.soundRate)  ...
```

```
                mb32(MakeQTMovieStatus.soundLength)];
else
        data = [mb32(MakeQTMovieStatus.frameRate * ...
                        MakeQTMovieStatus.timeScaleExpansion)  ...
                mb32(MakeQTMovieStatus.frameNumber * ...
                        MakeQTMovieStatus.timeScaleExpansion)];
end

y = [mb32(0) mbstring('mdhd') ...                   % Atom Header
     mb32(0) ...
     mb32(round(now*3600*24)) ...                   % Creation time
     mb32(round(now*3600*24)) ...                   % Modification time
     data ...
     mb16(0) mb16(0)];
y = SetAtomSize(y);

%%%%%%%%%%%%  mdia_atom %%%%%%%%%%%%%%
function y = mdia_atom(add_sound_p)
global MakeQTMovieStatus

if add_sound_p
        hdlr = hdlr_atom('mhlr', 'soun');
else
        hdlr = hdlr_atom('mhlr', 'vide');
end

y = [mb32(0) mbstring('mdia') ...                   % Atom Header
     mdhd_atom(add_sound_p) ...
     hdlr ...                                       % Handler Atom
     minf_atom(add_sound_p)];
y = SetAtomSize(y);


%%%%%%%%%%%%  minf_atom %%%%%%%%%%%%%%
function y = minf_atom(add_sound_p)
global MakeQTMovieStatus

if add_sound_p
        data = smhd_atom;
else
        data = vmhd_atom;
end

y = [mb32(0) mbstring('minf') ...                   % Atom Header
     data ...
     hdlr_atom('dhlr','alis') ...
```

```
     dinf_atom ...
     stbl_atom(add_sound_p)];
y = SetAtomSize(y);

%%%%%%%%%%%%  moov_atom %%%%%%%%%%%%%%
function y=moov_atom
global MakeQTMovieStatus
MakeQTMovieStatus.timeScale = MakeQTMovieStatus.frameRate * ...
                                MakeQTMovieStatus.timeScaleExpansion;

if MakeQTMovieStatus.soundLength > 0
        sound = trak_atom(1);
else
        sound = [];
end

y = [mb32(0) mbstring('moov') ...
     mvhd_atom udat_atom sound trak_atom(0) ];
y = SetAtomSize(y);

%%%%%%%%%%%%  mvhd_atom %%%%%%%%%%%%%%
function y=mvhd_atom
global MakeQTMovieStatus

fixed1 = bitshift(1,16);                        % Fixed point 1
frac1 = bitshift(1,30);                         % Fractional 1
if length(MakeQTMovieStatus.soundStart) > 0
        NumberOfTracks = 2;
else
        NumberOfTracks = 1;
end

                                        % Need to make sure its longer
                                        % of movie and sound lengths
MovieDuration = max(MakeQTMovieStatus.frameNumber / ...
                        MakeQTMovieStatus.frameRate, ...
                    MakeQTMovieStatus.soundLength / ...
                        MakeQTMovieStatus.soundRate);
MovieDuration = ceil(MovieDuration * MakeQTMovieStatus.timeScale);

y = [mb32(0) ...                        % Size
     mbstring('mvhd') ...               % Movie Data
     mb32(0) ...                        % Version and Flags
     mb32(0) ...                        % Creation Time (unknown)
     mb32(0) ...                        % Modification Time (unknown)
     mb32(MakeQTMovieStatus.timeScale) ...      % Movie's Time Scale
```

```
    mb32(MovieDuration) ...            % Movie Duration
    mb32(fixed1) ...                   % Preferred Rate
    mb16(255) ...                      % Preferred Volume
    mb16(0) ...                        % Fill
    mb32(0) ...                        % Fill
    mb32(0) ...                        % Fill
    mb32(fixed1) mb32(0) mb32(0) ...   % Transformation matrix (identity)
    mb32(0) mb32(fixed1) mb32(0) ...
    mb32(0) mb32(0) mb32(frac1) ...
    mb32(0) ...                        % Preview Time
    mb32(0) ...                        % Preview Duration
    mb32(0) ...                        % Poster Time
    mb32(0) ...                        % Selection Time
    mb32(0) ...                        % Selection Duration
    mb32(0) ...                        % Current Time
    mb32(NumberOfTracks)];             % Video and/or Sound?

y = SetAtomSize(y);

%%%%%%%%%%%%  raw_image_description %%%%%%%%%%%%%
function y = raw_image_description()
global MakeQTMovieStatus

fixed1 = bitshift(1,16);                      % Fixed point 1
codec = [12 'Photo - JPEG                    '];

y = [mb32(0) mbstring('jpeg') ...             % Atom Header
    mb32(0) mb16(0) mb16(0) mb16(0) mb16(1) ...
    mbstring('appl') ...
    mb32(1023) ...                            % Temporal Quality (perfect)
    mb32(floor(1023*MakeQTMovieStatus.spatialQual)) ...
    mb16(MakeQTMovieStatus.imageSize(2)) ...
    mb16(MakeQTMovieStatus.imageSize(1)) ...
    mb32(fixed1 * 72) mb32(fixed1 * 72) ...
    mb32(0) ...
    mb16(0) ...
    mbstring(codec) ...
    mb16(24) mb16(65535)];
y = SetAtomSize(y);


%%%%%%%%%%%%  raw_sound_description %%%%%%%%%%%%%
function y = raw_sound_description()
global MakeQTMovieStatus
y = [mb32(0) mbstring('twos') ...             % Atom Header
    mb32(0) mb16(0) mb16(0) mb16(0) mb16(0) ...
```

```
      mb32(0) ...
      mb16(MakeQTMovieStatus.soundChannels) ...
      mb16(16) ...                                % 16 bits per sample
      mb16(0) mb16(0) ...
      mb32(round(MakeQTMovieStatus.soundRate*65536))];
y = SetAtomSize(y);


%%%%%%%%%%%%% smhd_atom %%%%%%%%%%%%%%%
function y = smhd_atom()
y = SetAtomSize([mb32(0) mbstring('smhd') mb32(0) mb16(0) mb16(0)]);

%%%%%%%%%%%%% stbl_atom %%%%%%%%%%%%%%%
% Removed the stss atom since it seems to upset the PC version of QT
% and it is empty so it doesn't add anything.
% Malcolm - July 5, 1999
function y = stbl_atom(add_sound_p)
y = [mb32(0) mbstring('stbl') ...                 % Atom Header
      stsd_atom(add_sound_p) ...
      stts_atom(add_sound_p) ...
      stsc_atom(add_sound_p) ...
      stsz_atom(add_sound_p) ...
      stco_atom(add_sound_p)];
y = SetAtomSize(y);

%%%%%%%%%%%%% stco_atom %%%%%%%%%%%%%%%
function y = stco_atom(add_sound_p)
global MakeQTMovieStatus
if add_sound_p
        y = [mb32(0) mbstring('stco') mb32(0) mb32(1) ...
             mb32(MakeQTMovieStatus.soundStart)];
else
        y = [mb32(0) mbstring('stco') mb32(0) ...
             mb32(MakeQTMovieStatus.frameNumber) ...
             mb32(MakeQTMovieStatus.frameStarts)];
end
y = SetAtomSize(y);

%%%%%%%%%%%%% stsc_atom %%%%%%%%%%%%%%%
function y = stsc_atom(add_sound_p)
global MakeQTMovieStatus
if add_sound_p
        samplesperchunk = MakeQTMovieStatus.soundLength;
else
        samplesperchunk = 1;
end
```

```
y = [mb32(0) mbstring('stsc') mb32(0) mb32(1)   ...
     mb32(1) mb32(samplesperchunk) mb32(1)];
y = SetAtomSize(y);

%%%%%%%%%%%%%  stsd_atom %%%%%%%%%%%%%%%
function y = stsd_atom(add_sound_p)
if add_sound_p
        desc = raw_sound_description;
else
        desc = raw_image_description;
end

y = [mb32(0) mbstring('stsd') mb32(0) mb32(1) desc];
y = SetAtomSize(y);

%%%%%%%%%%%%%  stss_atom %%%%%%%%%%%%%%%
function y = stss_atom()
y = SetAtomSize([mb32(0) mbstring('stss') mb32(0) mb32(0)]);

%%%%%%%%%%%%%  stsz_atom %%%%%%%%%%%%%%%
function y = stsz_atom(add_sound_p)
global MakeQTMovieStatus
if add_sound_p
        y = [mb32(0) mbstring('stsz') mb32(0) mb32(2) ...
             mb32(MakeQTMovieStatus.soundLength)];
else
        y = [mb32(0) mbstring('stsz') mb32(0) mb32(0) ...
             mb32(MakeQTMovieStatus.frameNumber) ...
             mb32(MakeQTMovieStatus.frameLengths)];
end
y = SetAtomSize(y);

%%%%%%%%%%%%%  stts_atom %%%%%%%%%%%%%%%
function y = stts_atom(add_sound_p)
global MakeQTMovieStatus
if add_sound_p
        count_duration = [mb32(MakeQTMovieStatus.soundLength) mb32(1)];
else
        count_duration = [mb32(MakeQTMovieStatus.frameNumber) ...
                mb32(MakeQTMovieStatus.timeScaleExpansion)];
end

y = SetAtomSize([mb32(0) mbstring('stts') mb32(0) mb32(1) count_duration]);

%%%%%%%%%%%%%  trak_atom %%%%%%%%%%%%%%%
```

```
function y = trak_atom(add_sound_p)
global MakeQTMovieStatus

y = [mb32(0) mbstring('trak') ...                % Atom Header
       tkhd_atom(add_sound_p) ...                % Track header
       edts_atom(add_sound_p) ...                % Edit List
       mdia_atom(add_sound_p)];
y = SetAtomSize(y);

%%%%%%%%%%%%%%  tkhd_atom %%%%%%%%%%%%%%%
function y = tkhd_atom(add_sound_p)
global MakeQTMovieStatus

fixed1 = bitshift(1,16);                         % Fixed point 1
frac1 = bitshift(1,30);                          % Fractional 1 (CHECK THIS)

if add_sound_p > 0
       duration = MakeQTMovieStatus.soundLength / ...
                  MakeQTMovieStatus.soundRate * ...
                  MakeQTMovieStatus.timeScale;
else
       duration = MakeQTMovieStatus.frameNumber / ...
                  MakeQTMovieStatus.frameRate * ...
                  MakeQTMovieStatus.timeScale;
end
duration = ceil(duration);

y = [mb32(0) mbstring('tkhd') ...        % Atom Header
    mb32(15) ...                         % Version and flags
    mb32(round(now*3600*24)) ...         % Creation time
    mb32(round(now*3600*24)) ...         % Modification time
    mb32(MakeQTMovieStatus.trackNumber) ...
    mb32(0) ...
    mb32(duration) ...                   % Track duration
    mb32(0) mb32(0) ...                  % Offset and priority
    mb16(0) mb16(0) mb16(255) mb16(0) ...      % Layer, Group, Volume, fill
    mb32(fixed1) mb32(0) mb32(0) ...     % Transformation matrix (identity)
    mb32(0) mb32(fixed1) mb32(0) ...
    mb32(0) mb32(0) mb32(frac1)];

if add_sound_p
       y = [y mb32(0) mb32(0)];          % Zeros for sound
else
       y = [y mb32(fliplr(MakeQTMovieStatus.imageSize)*fixed1)];
end
y= SetAtomSize(y);
```

```
MakeQTMovieStatus.trackNumber = MakeQTMovieStatus.trackNumber + 1;

%%%%%%%%%%%%%  udat_atom %%%%%%%%%%%%%%%
function y = udat_atom()
atfmt = [64 double('fmt')];
atday = [64 double('day')];

VersionString = 'Matlab␣MakeQTMovie␣version␣April␣7,␣2000';

y = [mb32(0) mbstring('udta') ...
        SetAtomSize([mb32(0) atfmt mbstring(['Created␣' VersionString])]) ...
        SetAtomSize([mb32(0) atday '␣␣' date])];
y = SetAtomSize(y);


%%%%%%%%%%%%%  vmhd_atom %%%%%%%%%%%%%%%
function y = vmhd_atom()

y = SetAtomSize([mb32(0) mbstring('vmhd') mb32(0) ...
    mb16(64) ...                          % Graphics Mode
    mb16(0) mb16(0) mb16(0)]);            % Op Color
```
